# ASB2 inhibits lipid accumulation to promote ILC1 homeostatic fitness and anti-tumor immunity in the mouse liver

**DOI:** 10.1038/s41467-026-75517-4

**Published:** 2026-07-17

**Authors:** Boqun Bao, Xianwei Wang, Yawen Chen, Xiaodong Zheng, Yongyan Chen, Haoyu Sun, Guoliang Cui, Rui Sun, Zhigang Tian, Hui Peng

**Affiliations:** 1 State Key Laboratory of Immune Response and Immunotherapy, the Institute of Immunology, Biomedical Sciences and Health Laboratory of Anhui Province, Center for Advanced Interdisciplinary Science and Biomedicine of IHM, School of Basic Medical Sciences, Division of Life Sciences and Medicine, University of Science and Technology of China, Hefei, China; 2 Department of Immunology, School of Basic Medical Sciences, Fudan University, Shanghai, China; 3 Department of Medical Oncology, Fudan University Shanghai Cancer Center, Shanghai, China; 4 Department of Oncology, Shanghai Medical College, Fudan University, Shanghai, China

**Keywords:** Metastasis, Lipids, Innate lymphoid cells

## Abstract

Type 1 innate lymphoid cells (ILC1) are abundant in the adult liver and are pivotal for immune surveillance and modulation, but the regulation of their maintenance and functionality remains underexplored. Here, we re-analyze published single-cell RNA- sequencing data and find increased expression of *Asb2* in ILC1s from adult mouse livers. Conditional ablation of *Asb2* in NKp46^+^ cells, depleting ASB2 in ILC1s and NK cells, in mice impairs ILC1 survival and reduces adult liver ILC1 numbers. Proteomics and bulk RNA-sequencing reveal enriched lipid metabolism pathways in ASB2-deficient ILC1s, concomitant with increased lipid storage. Importantly, pharmacological inhibition of lipid synthesis prevents the apoptosis of ASB2-deficient ILC1s in vitro. In a mouse model of colorectal cancer liver metastasis, we find increased ILC1 lipid storage, and conditional *Asb2* deficiency in ILC1 cells exacerbates liver metastasis progression. Conversely, inhibiting lipid accumulation in wild-type colorectal cancer-bearing mice prolongs animal survival, potentially via promoting ILC1-mediated anti-tumor immunity. Thus, our study uncovers ASB2-regulated lipid metabolism as a gatekeeper for ILC1 homeostatic fitness and tumor surveillance, highlighting potential ILC1-based therapeutic strategies against liver tumors.

## Abstract (web summary)

Type 1 innate lymphoid cells (ILC1s) are integral for immunity, but how liver ILC1s are regulated is incompletely understood. Here, the authors find ASB2 to limit detrimental ILC1 lipid accumulation in adult mouse livers, thereby contributing to ILC1 homeostasis and to anti-tumor immunity in a liver metastasis model.

## Introduction

Innate lymphoid cells (ILC) are heterogeneous populations that are grouped into conventional NK cells (cNK), ILC1s, ILC2s, ILC3s, and lymphoid tissue inducer (LTi) cells, based on their transcriptional requirement and functional specialization^1–3^. Within the ILC family, ILC1s and cNK cells are categorized as group 1 ILCs, based on their capability of producing interferon-gamma (IFN-γ) and T-bet expression, and both cell types play important roles in early anti-viral and anti-tumor immunity^4–7^. Despite such similarities, mouse ILC1s can be further distinguished from cNK cells by their expression of the transcription factor Hobit and surface markers CD49a, CD200R1, and CXCR6^1,5,8–10^. Furthermore, ILC1s exhibit a more restricted tissue distribution compared to cNK cells, with a notable enrichment in specific tissues such as the liver^11,12^. Although the liver functions as an essential metabolic organ and plays important roles in glycogenesis and lipogenesis^13,14^, it remains unknown whether and how metabolic pathways influence liver ILC1 maintenance and function.

Dynamic reprogramming of cellular metabolic networks critically orchestrates the functional specialization of ILCs. Recent studies demonstrate that increased intracellular lipid content driven by IL-2/IL-15 enhances cNK cell activity^15^. Conversely, excessive lipid accumulation in cNK cells suppresses their effector function in pathological contexts, such as the tumor microenvironment and obesity^16–19^. In patients with high-grade serous ovarian carcinoma (HGSOC), excessive polar lipids in the ascites reduce lipid droplet content and disrupt the lipid metabolic homeostasis of cNK cells, impairing their cytotoxic function^20^. Beyond cNK cells, lipid metabolism also regulates the function of ILC2s^21^. Moreover, acute exposure to a high-fat diet impairs ILC3 functions and gut homeostasis^22^. Despite such progress, the regulation of ILC1s by lipid metabolism remains poorly explored.

Ankyrin repeat-containing protein with a suppressor of cytokine signaling box-2 (ASB2) serves as a substrate-recognition subunit within E3 ubiquitin ligase complexes, facilitating the specific targeting of proteins for ubiquitination and subsequent proteasomal degradation^23^. ASB2 regulates actin cytoskeleton remodeling by mediating the degradation of filamins, which is critical for hematopoietic differentiation and retinoic acid-induced growth inhibition in myeloid leukemia cells^24,25^. Recent research unveils that ASB2 regulates the migration of dendritic cells (DC) and T helper 2 (Th2) cells^26,27^, while the regulatory role of ASB2 in ILCs remains unclear.

In this study, we explore the regulatory role of ASB2 in mouse liver ILC1s by analyzing published single-cell RNA-sequencing (scRNA-seq) data and investigating conditional *Asb2* knockout mice alongside a mouse model of colorectal cancer liver metastasis. We demonstrate that, as a highly expressed molecule in mouse liver ILC1s, ASB2 sustains ILC1 survival and anti-tumor activities. By employing a multi-omics approach integrating proteomics and bulk RNA-seq, and by utilizing pharmacological interventions, we show that ASB2 prevents ILC1 apoptosis through inhibiting lipid accumulation. These findings uncover a critical metabolic axis for mouse ILC1 homeostasis and provide insights for therapeutic strategies against liver tumors.

## Results

### ASB2 is highly expressed in adult mouse liver ILC1s

**Fig. 1:**
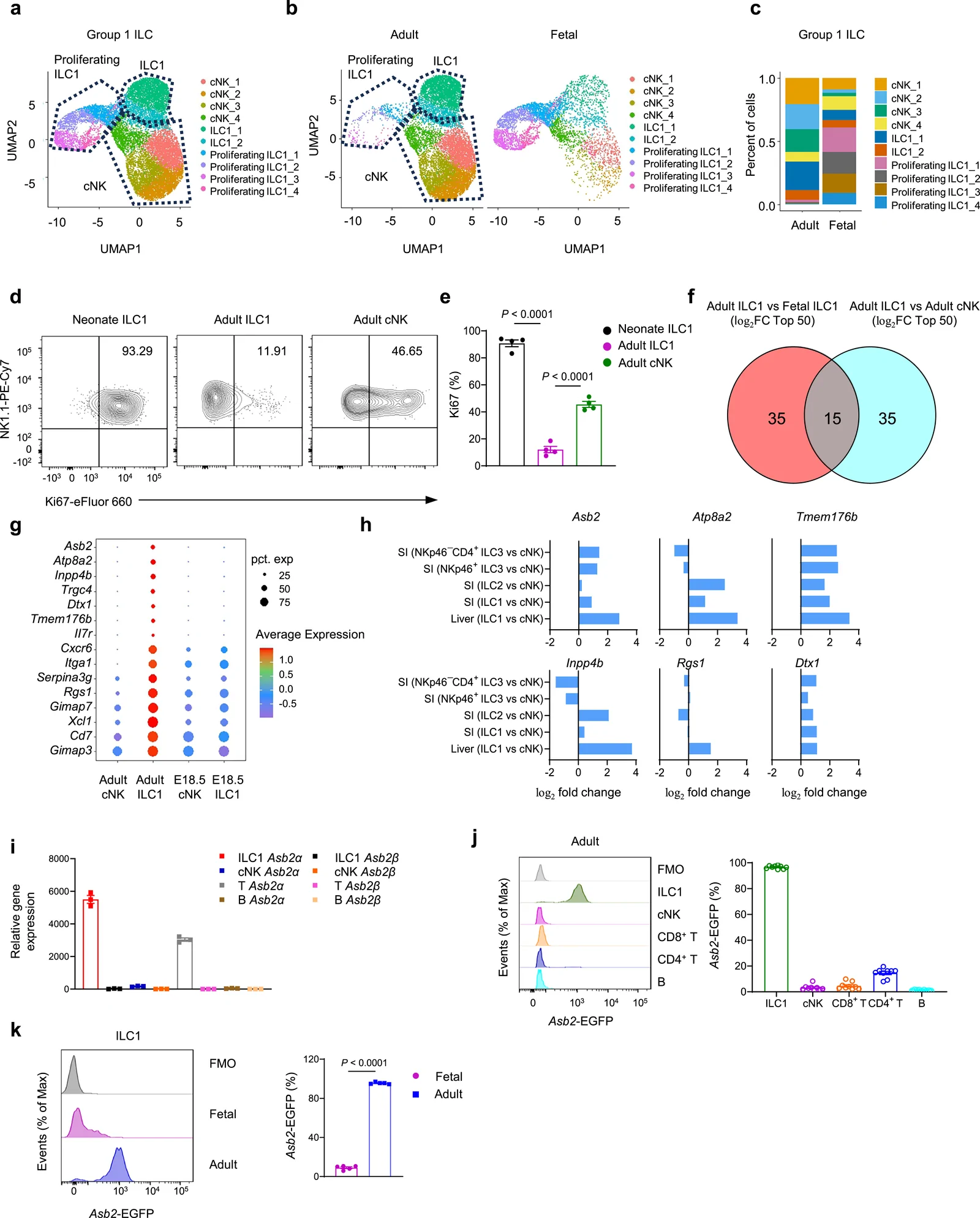
Adult mouse liver ILC1s express high levels of ASB2. **a–c** Integrated (**a**) and separated UMAP plots (**b**) and cluster frequency (**c**) of group 1 ILCs from fetal and adult liver. Raw data were extracted from GSE202475. **d, e** Representative flow cytometry contour plots (**d**) and percentages (**e**) of Ki67 expression in neonatal ILC1s (*n* = 4; sex undetermined), adult ILC1s (*n* = 4; male), and adult cNK cells (*n* = 4; male). **f** Overlap of top 50 differentially expressed genes (DEG) in adult ILC1s versus cNK cells and adult ILC1s versus fetal ILC1s. **g** Dot plot of the top 15 most discriminatory genes for each group. The color scale represents average expression, and the dot size indicates the fraction of cells expressing the indicated gene. **h** The log_2_ fold changes of indicated genes in ILC1s, ILC2s, and ILC3s from indicated tissues after normalization to cNK cells. Raw data were extracted from GSE37448. **i** Quantitative PCR analysis of *Asb2α* and *Asb2β* mRNA expression in liver ILC1s, cNK, T and B cells from C57BL/6 mice (*n* = 3 per group; male). Gene expression is normalized to that of the housekeeping gene *β-actin* and is presented relative to *Asb2β* in B cells. **j** Histogram (left) and frequencies (right) of *Asb2*-EGFP expression on liver lymphocytes from adult *Asb2*^EGFP^ mice (*n* = 9 per group; male). **k** Histogram (left) and frequencies (right) of *Asb2*-EGFP expression on liver ILC1s from fetal (*n* = 5; sex undetermined) and adult (*n* = 5; female) *Asb2*^EGFP^ mice. Data are from one representative experiment of two (**i**, **k**) or three (**d**, **e**, **j**) independent experiments. Samples were compared using one-way ANOVA (**e**) or two-tailed unpaired Student’s *t* tests (**k**) and were presented as means ± SEM. Group 1 ILC: group 1 innate lymphoid cell; ILC1: type 1 innate lymphoid cell; cNK: conventional natural killer cell; ILC2: type 2 innate lymphoid cell; ILC3: type 3 innate lymphoid cell; FMO: fluorescence minus one.

ILC1s emerge during fetal life and stably persist as enriched populations in the adult liver^10,28,29^. However, ILC1s show heterogeneity in subset composition, which dynamically changes during ontogeny^7,28^. To explore molecular mechanisms underlying specific regulation of adult mouse ILC1 waves, we re-analyzed published scRNA-seq data of fetal and adult mouse liver ILC1s (GSE202475). Group 1 ILCs could be subdivided into ten clusters. By analyzing the characteristic markers of cNK cells and ILC1s, we could identify 4 clusters of cNK cells expressing *Itga2* (CD49b) and *Eomes*, and 6 clusters of ILC1s expressing *Itga1* (CD49a), *Cd200r1*, and *Cxcr6*, among which 4 ILC1 clusters expressed high levels of *Mki67*, termed as proliferating ILC1s (Fig. 1a, Supplementary Fig. 1a). Compared with adult liver, fetal liver mainly harbored proliferating ILC1s, suggesting significant difference between fetal and adult ILC1s (Fig. 1b, c). In line with this, flow cytometric analysis of Ki67 staining further revealed that adult mouse liver ILC1s displayed substantially lower proliferative rate than neonatal ILC1s, as well as adult cNK cells (Fig. 1d, e). This observation, together with hepatic enrichment of ILC1s, suggests that their maintenance likely relies on non-proliferative mechanisms. By integrated comparison of the top 50 up-regulated genes in adult liver ILC1s versus fetal liver ILC1s, or adult cNK cells, we identified 15 up-regulated genes with adult ILC1-enriched expression (Fig. 1f, g, Supplementary Fig. 1b). Among them, *Il7r, Rgs1*, and *Inpp4b* have been identified as characteristic genes or regulators of tissue-resident ILC1s^30–34^. While *Asb2* exhibited liver ILC1-restricted up-regulation after normalization to cNK cells, *Atp8a2, Tmem176b* and *Dtx1* were either up-regulated in other ILCs (ILC2/ILC3) or uniformly expressed across pan-tissue ILC1s^35^ (GSE37448) (Fig. 1h). The *Asb2* gene encodes two transcripts, namely *Asb2a* and *Asb2b*^25,26,36^. RT-qPCR analysis revealed that mouse liver ILC1s predominantly expressed the *Asb2a* isoform, which exhibited intermediate expression in T cells and low levels in liver cNK and B cells; in contrast, *Asb2β* was negligibly expressed across all examined cell types (Fig. 1i). Then, we generated an *Asb2*^EGFP^ reporter mouse line, which cannot distinguish the expression of these two ASB2 isoforms. Flow cytometric analysis of these mice further confirmed that *Asb2* was expressed in adult liver ILC1s, with negligible expression in early-life ILC1s or adult cNK cells as well as adaptive lymphocytes (Fig. 1j, k, Supplementary Figs. 1c, 2a). Using *Asb2*^EGFP^ mice, we also evaluated *Asb2* expression in various immune cells across tissues. We found that *Asb2* was highly expressed by most tissue-resident ILC subsets (Supplementary Figs. 1c, 2b, 3a) and natural killer T (NKT) cells (Supplementary Figs. 1c, 2a) in multiple tissues. However, *Asb2* expression was relatively low in mucosal-associated invariant T cells (MAIT), CD4⁺ T cells and CD8⁺ T cells in the liver (Fig. 1j, Supplementary Figs. 1c, 2a) and rarely detected in circulating lymphocytes in the lung (Supplementary Figs. 1c, 3a) and spleen (Supplementary Figs. 1c, 3b). ASB2 is abundantly expressed in intestinal cNK cells (Supplementary Figs. 1c, 2b), in contrast to its absence in cNK cells from other tissues, likely due to the unique tissue microenvironment. Taken together, these results indicate that ASB2 is highly expressed by adult mouse liver ILC1s.

### ASB2 supports the maintenance of adult mouse liver ILC1s

**Fig. 2:**
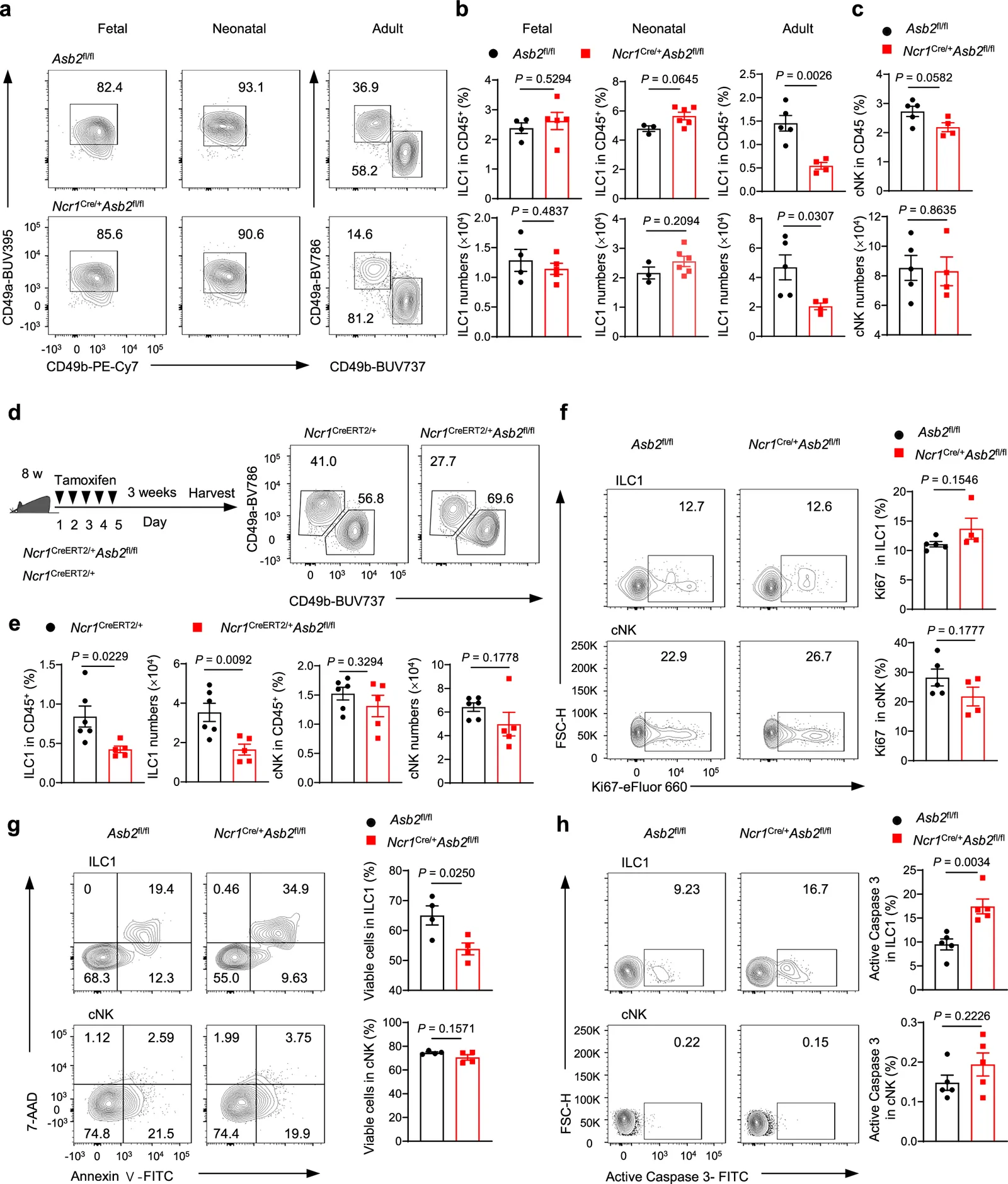
ASB2 deficiency causes a reduced adult mouse liver ILC1 pool size. **a** Representative flow cytometry plots of liver ILC1s from fetal, neonatal and adult *Ncr1*^Cre/+^*Asb2*^fl/fl^ and *Asb2*^fl/fl^ mice. **b** Frequencies (up) and absolute numbers (down) of liver ILC1s from fetal (*Asb2*^fl/fl^: *n* = 4; *Ncr1*^Cre/+^*Asb2*^fl/fl^: *n* = 5; sex undetermined), neonatal (*Asb2*^fl/fl^: *n* = 3; *Ncr1*^Cre/+^*Asb2*^fl/fl^: *n* = 6; sex undetermined) and adult (*Asb2*^fl/fl^: *n* = 5; *Ncr1*^Cre/+^*Asb2*^fl/fl^: *n* = 4; female) *Ncr1*^Cre/+^*Asb2*^fl/fl^ and *Asb2*^fl/fl^ mice. **c** Frequencies and absolute numbers of liver cNK cells from adult *Ncr1*^Cre/+^*Asb2*^fl/fl^ (*n* = 4; female) and *Asb2*^fl/fl^ mice (*n* = 5; female). **d**, **e** Representative plots (**d**) and percentages and absolute numbers (**e**) of liver ILC1s and cNK cells from *Ncr1*^CreERT2/+^ (*n* = 6; male) and *Ncr1*^CreERT2/+^*Asb2*^fl/fl^ (*n* = 5; male) mice after intraperitoneal injection (i.p.) treatment with tamoxifen. **f** Representative plots and proportions of Ki67^+^ among liver ILC1s and cNK from adult *Ncr1*^Cre/+^*Asb2*^fl/fl^ (*n* = 4; female) and *Asb2*^fl/fl^ (*n* = 5; female) mice. **g** Representative plots and proportions of viable (Annexin Ⅴ^−^7-AAD^−^) cells among liver ILC1s and cNK cells from adult *Ncr1*^Cre/+^*Asb2*^fl/fl^ and *Asb2*^fl/fl^ mice (*n* = 4 per group; male)**. h** Representative plots and proportions of active caspase 3^+^ cells among liver ILC1s and cNK from adult *Ncr1*^Cre/+^*Asb2*^fl/fl^ and *Asb2*^fl/fl^ mice (*n* = 5 per group; male**)**. Data are from one representative experiment of two (**a**, **b**, **d, e, h **) or three (**c**, **f**, **g**) independent experiments. Samples were all compared using two-tailed unpaired Student’s *t*-tests and were presented as means ± SEM. ILC1: type 1 innate lymphoid cell; cNK: conventional natural killer cell.

To explore the functions of ASB2 in mouse ILC1s, we generated *Asb2*^fl/fl^ *-*conditional knockout mice (*Ncr1*^Cre/+^*Asb2*^fl/fl^), which had cell-intrinsic deletion of *Asb2* in ILC1s and cNK cells. The loss of *Asb2* was validated at the mRNA level in liver NK1.1^+^NKp46^+^ cells, a mixture of cNK cells and ILC1s, from *Ncr1*^Cre/+^*Asb2*^fl/fl^ mice (Supplementary Fig. 4a). We found that adult liver ILC1s declined significantly in ASB2-deficient mice relative to either *Asb2*^fl/fl^ or *Ncr1*^Cre/+^ controls (Fig. 2a, b, Supplementary Fig. 5a), whereas fetal and neonatal ILC1s were not affected (Fig. 2a, b). Meanwhile, the frequency and number of cNK cells in the liver and spleen of adult *Ncr1*^Cre/+^*Asb2*^fl/fl^ mice remained unchanged (Fig. 2c, Supplementary Figs. 4b, 5a, b). To further confirm the roles of ASB2 in regulating adult mouse ILC1s, we also obtained *Ncr1*^CreERT2/+^*Asb2*^fl/fl^ mice, which enable the temporal control of *Asb2* deletion by tamoxifen injection. Consistently, tamoxifen-induced ablation of *Asb2* in 8-week-old mice also resulted in decreased liver ILC1s (Fig. 2d, e). In *Vav*^Cre/+^*Asb2*^fl/fl^ mice, the proportion and absolute number of liver ILC1s were also markedly decreased (Supplementary Fig. 4c). Other lymphocyte populations, including ILC2s, ILC3s, T cells and NKT cells, exhibited comparable frequency and cell numbers between *Vav*^Cre/+^*Asb2*^fl/fl^ and *Asb2*^fl/fl^ mice (Supplementary Fig. 4d), indicative of ASB2 as a specific regulator for adult mouse liver ILC1s.

We then sought to investigate how ASB2 regulated adult mouse liver ILC1 maintenance. *Asb2* was barely expressed in CD45^+^Lin^−^ mixed progenitors and PD-1^+^ ILC precursors (ILCP), suggesting that ASB2 may exert limited influence on early development of ILC1s (Supplementary Fig. 4e, f). We examined expression of the proliferation marker Ki67, and found that it remained unchanged in ASB2-deficient liver ILC1s (Fig. 2f). Following in vitro culture, the proportion of viable ILC1s from *Ncr1*^Cre/+^*Asb2*^fl/fl^ mice was decreased (Fig. 2g, Supplementary Fig. 5c), and the active caspase 3 expression was significantly higher than that from *Asb2*^fl/fl^ mice (Fig. 2h). Collectively, these results demonstrate that ASB2 specifically supports the homeostatic fitness of adult mouse ILC1s by maintaining their survival.

### ASB2 inhibits lipid storage in mouse ILC1s

To further explore the downstream mechanism regulated by ASB2 that may control the maintenance of mouse liver ILC1s, we performed bulk RNA-seq of sorted ILC1s from *Ncr1*^Cre/+^*Asb2*^fl/fl^ mice and *Asb2*^fl/fl^ mice, respectively. We identified 211 up-regulated genes and 150 down-regulated genes in ASB2-deficient ILC1s compared with *Asb2*^fl/fl^ counterparts (Supplementary Fig. 6a, Supplementary Data 1). Gene ontology (GO) enrichment analysis showed that these up-regulated genes were enriched in the apoptotic signaling pathway, which is consistent with the increased susceptibility of ASB2-deficient ILC1s to death (Supplementary Fig. 6b, Supplementary Data 2). Specifically, *Asb2* deficiency led to the enrichment of genes related to regulation of lipid localization, lipid metabolic process, and lipid homeostasis (Supplementary Fig. 6b, Supplementary Data 2). Gene-set enrichment analysis consistently revealed that ASB2-deficient ILC1s were more enriched in genes involved in lipid storage, lipid biosynthetic process, and lipid transport (Supplementary Fig. 6c). These results suggest that ASB2 might be involved in reprogramming lipid metabolism of liver ILC1s.

**Fig. 3:**
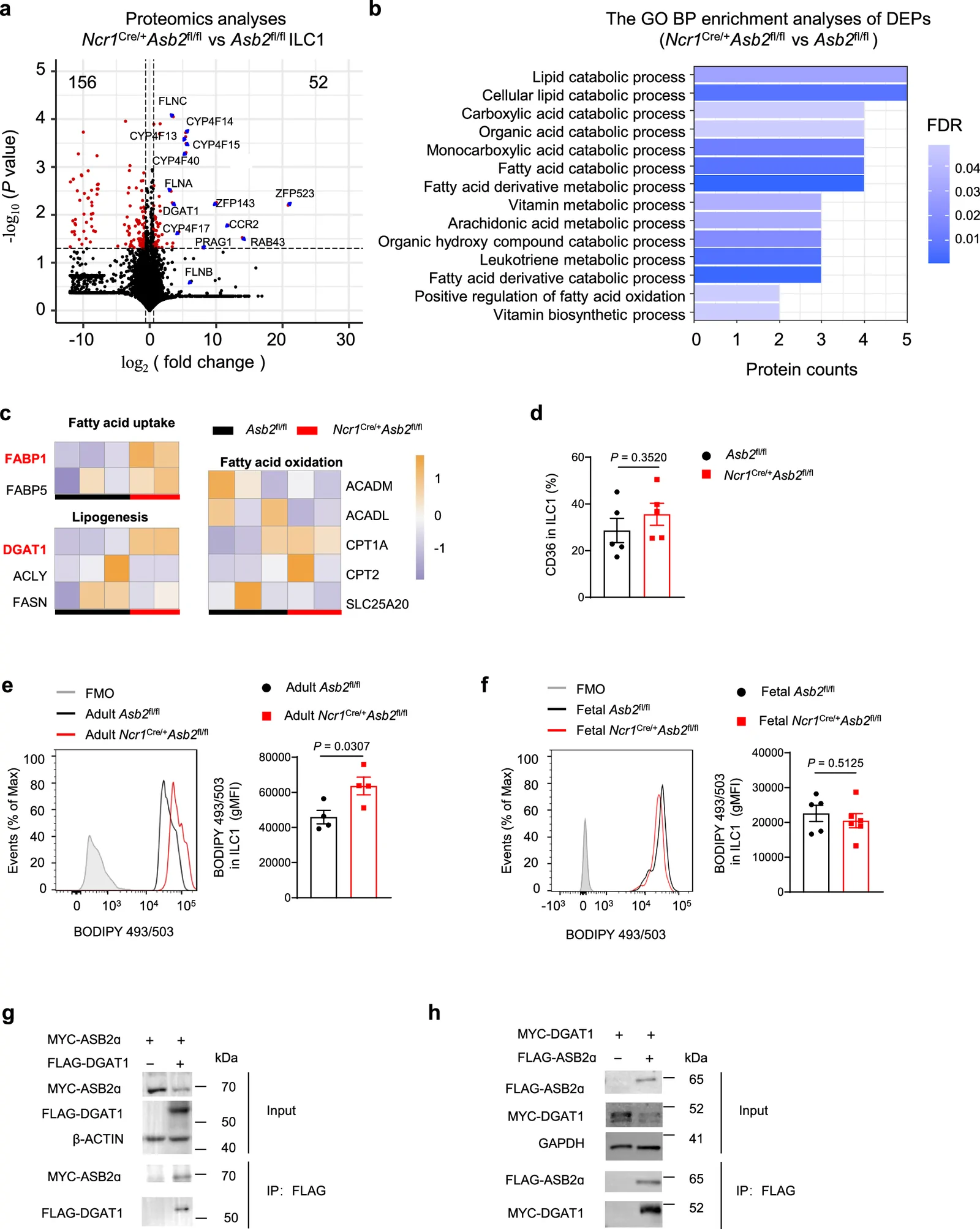
ASB2 deficiency drives lipid storage in mouse ILC1s. **a–c** Proteomics was performed on ILC1s (CD45^+^CD3^−^CD19^−^NK1.1^+^NKp46^+^CD49b^−^CD49a^+^) isolated from livers in *Ncr1*^Cre/+^*Asb2*^fl/fl^ or *Asb2*^fl/fl^ mice (each group pooled from 24 mice of both sexes). **a** Volcano plot showing differentially expressed proteins (DEP) between ILC1s from *Ncr1*^Cre/+^*Asb2*^fl/fl^ and *Asb2*^fl/fl^ mice. Lines indicate a 1.5-fold difference between ILC1 cells from *Ncr1*^Cre/+^*Asb2*^fl/fl^ and *Asb2*^fl/fl^ mice (*p* < 0.05). **b** Gene ontology (GO) enrichment (biological processes) analysis showing the enriched pathways of up-regulated DEPs. **c** Heatmap showing protein expression levels related to fatty acid uptake, fatty acid oxidation and lipogenesis. **d** The expression of CD36 by liver ILC1s from *Ncr1*^Cre/+^*Asb2*^fl/fl^ and *Asb2*^fl/fl^ mice (*n* = 5 per group; female). **e** Representative histograms (left) and gMFI of BODIPY 493/503 staining (right) in liver ILC1s from adult *Ncr1*^Cre/+^*Asb2*^fl/fl^ and *Asb2*^fl/fl^ mice (*n* = 4 per group; male). **f** Representative histograms (left) and expression of BODIPY 493/503 staining (right) in liver ILC1s from fetal *Ncr1*^Cre/+^*Asb2*^fl/fl^ (*n* = 6; sex undetermined) and *Asb2*^fl/fl^ mice (*n* = 5; sex undetermined). **g** IP and immunoblot analysis of the interaction between Myc-tagged ASB2ɑ and Flag-tagged DGAT1. The input is whole-cell lysates. **h** IP and immunoblot analysis of the interaction between Myc-tagged DGAT1 and Flag-tagged ASB2ɑ. The input is whole-cell lysates. Data are from one representative experiment of two (**d**, **f**, **h**) or three (**e**, **g**) independent experiments. Samples were compared using two-tailed unpaired Student’s *t* tests (**a**, **d–f**) and were presented as means ± SEM. ILC1: type 1 innate lymphoid cell; DEP: differentially expressed proteins; FMO: fluorescence minus one.

ASB2 is the specificity subunit of Cullin 5-RING E3 ubiquitin ligase complexes, and its role in mediating target protein degradation has been established^37^. We performed label-free proteomic analysis on sorted ILC1s from *Ncr1*^Cre/+^*Asb2*^fl/fl^ and *Asb2*^fl/fl^ mice, and identified 208 differentially expressed proteins (DEP), with 52 DEPs up-regulated and 156 down-regulated proteins in ASB2-deficient ILC1s (Fig. 3a, Supplementary Data 3). FLNA and FLNC, known substrates of ASB2^24,37^, were significantly up-regulated in ASB2-deficient ILC1s (Fig. 3a, Supplementary Data 3). Consistent with the RNA-seq results, GO enrichment analysis of the up-regulated proteins revealed fatty acid metabolic-related proteins were enriched in ASB2-deficient ILC1s (Fig. 3b, Supplementary Data 4). Collectively, both proteomics and RNA-seq analysis identified ASB2 as a key molecule in regulating lipid metabolism of mouse liver ILC1s. To explore how ASB2 regulates lipid metabolism of liver ILC1s, we analyzed the major enzymes involved in fatty acid uptake, fatty acid oxidation and lipogenesis in the proteomics dataset. FABP1, a liver-specific fatty acid-binding protein (FABP) that is critical for fatty acid uptake^38^, and DGAT1 (Diacylglycerol O-Acyltransferase 1), a key enzyme in triglyceride synthesis that facilitates lipid storage in cells^39^, were significantly up-regulated in ASB2-deficient ILC1s (Fig. 3c). Furthermore, no significant changes in CD36 expression, a key receptor for lipid uptake, were observed in ASB2-deficient ILC1s via flow cytometry (Fig. 3d). Thus, ASB2 may be involved in regulating lipid transport and accumulation in mouse liver ILC1s. Consistently, we observed significantly increased total neutral lipid content in adult liver ILC1s of *Ncr1*^Cre/+^*Asb2*^fl/fl^ mice, but not in cNK cells, as evidenced by BODIPY 493/503 staining (Fig. 3e, Supplementary Fig. 5d). By contrast, neutral lipid levels in fetal liver ILC1s were not altered between *Ncr1*^Cre/+^*Asb2*^fl/fl^ and *Asb2*^fl/fl^ mice (Fig. 3f). To identify the lipid species responsible for lipid accumulation in mouse ASB2-deficient ILC1s, we performed flow cytometry using specific probes to detect distinct lipid species. In fixed cells, we observed a significant decrease in intracellular C12 fatty acid levels, a moderate increase in cholesterol levels, and no alteration in C16 fatty acids levels, alongside an increase in BODIPY 493/503 staining, in ASB2-deficient ILC1s (Supplementary Fig. 6d). Given ASB2 deficiency-induced DGAT1 upregulation (Fig. 3c), these results suggest that fatty acids in ASB2-deficient ILC1s are likely consumed for enhanced DGAT1-mediated triglyceride synthesis (Supplementary Fig. 6d). Furthermore, live cell uptake assays showed no difference of C12 and C16 levels between control and ASB2-deficient ILC1s, indicating that ASB2 does not affect the uptake of free fatty acid in mouse ILC1s (Supplementary Fig. 6e). To further explore the regulatory relationship between ASB2 and DGAT1, we then performed co-immunoprecipitation (Co-IP) assays. The results showed that ASB2α and DGAT1 could be co-precipitated from cell lysates, indicating that ASB2α physically associates with DGAT1 (Fig. 3g, h). Taken together, these findings demonstrate that ASB2 associates with DGAT1 and inhibits lipid accumulation in adult liver ILC1s.

### Excessive lipid accumulation driven by ASB2 deficiency dampens mouse ILC1 survival

**Fig. 4:**
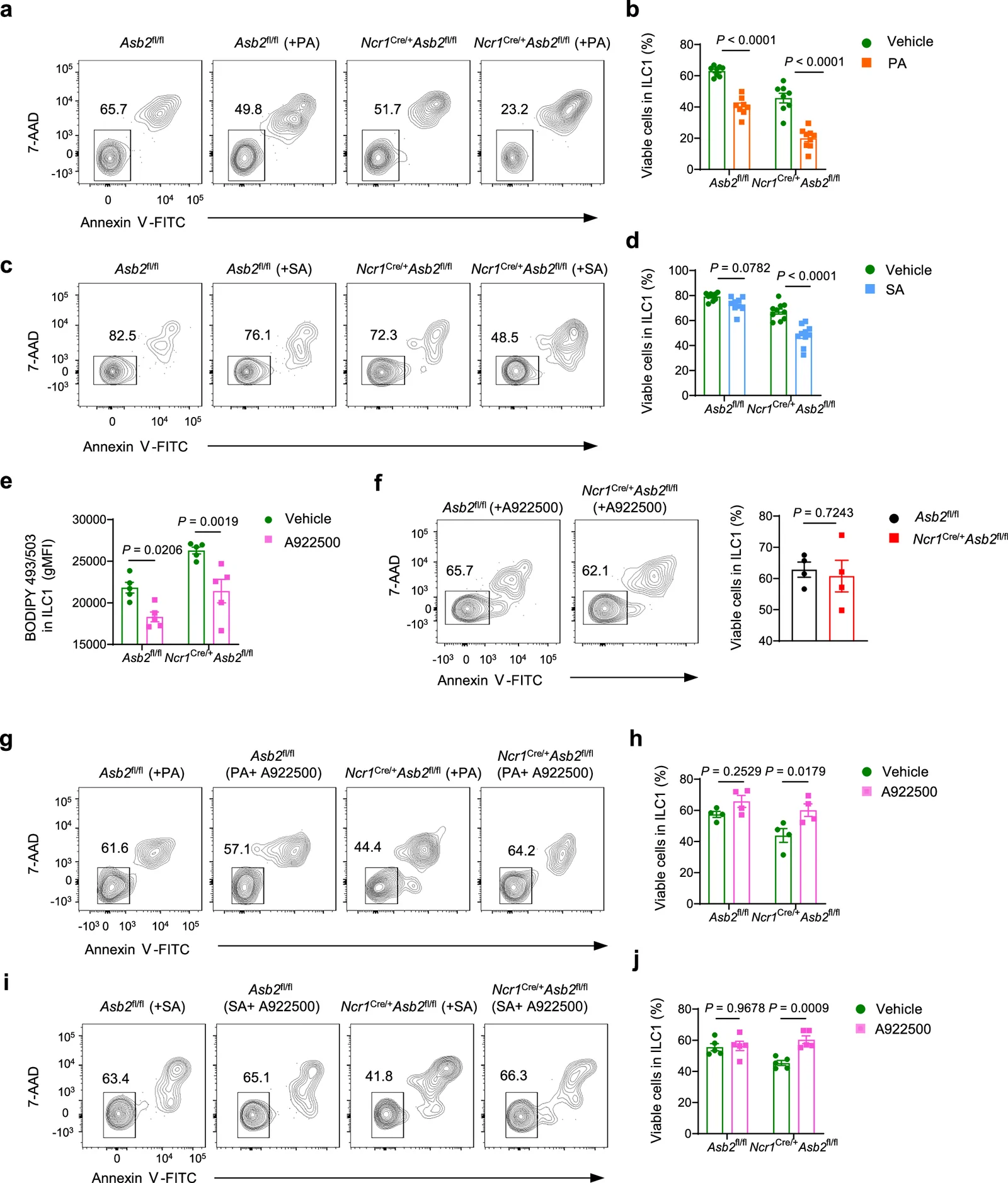
Lipid synthesis inhibition prevents the apoptosis of mouse ASB2-deficient ILC1s. **a**, **b** Representative flow cytometry plots (**a**) and percentages (**b**) of viable cells (Annexin Ⅴ^−^7-AAD^−^) of ILC1s that were cultured with 100 μM PA for 24 h (*Asb2*^fl/fl^ + Vehicle: *n* = 8; *Asb2*^fl/fl^ + PA: *n* = 8; *Ncr1*^Cre/+^*Asb2*^fl/fl^ + Vehicle: *n* = 8; *Ncr1*^Cre/+^*Asb2*^fl/fl^ + PA: *n* = 9; pooled from two independent experiment with one using female mice and one using male mice). **c**, **d** Representative flow cytometry contour plots (**c**) and percentages (**d**) of viable cells in ILC1s that were cultured with 50 μM SA for 24 h (*Asb2*^fl/fl^ + Vehicle: *n* = 9; *Asb2*^fl/fl^ + SA: *n* = 9; *Ncr1*^Cre/+^*Asb2*^fl/fl^ + Vehicle: *n* = 10; *Ncr1*^Cre/+^*Asb2*^fl/fl^ + SA: *n* = 10; male). **e** gMFI of BODIPY-493/503 staining in ILC1s from adult *Ncr1*^Cre/+^*Asb2*^fl/fl^ and *Asb2*^fl/fl^ mice, cultured with or without 3 μM A922500 for 24 h (*n* = 5 per group; female). **f** Representative flow cytometry contour plots (left) and percentages (right) of viable cells in ILC1s that were cultured with A922500 (3 μM) for 24 h (*n* = 4 per group; male). **g**, **h** Representative flow cytometry plots (**g**) and percentages (**h**) of viable ILC1 with 100 μM PA treatment and 3 μM A922500 for 24 h (*n* = 4 per group; male). **i, j** Representative flow cytometry plots (**i**) and percentages (**j**) of viable ILC1 with 50 μM SA treatment and 3 μM A922500 for 24 h (*n* = 5 per group; male). Data are from one representative experiment of two (**e**, **g**, **h**) or three (**f**, **i**, **j**) independent experiments or are pooled from two independent experiments (**a–d**). Samples were compared using two-tailed unpaired Student’s *t* test (**f**) or two-way ANOVA (**b**, **d**, **e**, **h**, **j**) and were presented as means ± SEM. ILC1: type 1 innate lymphoid cell; PA: palmitic acid; SA: steric acid.

Prior studies have reported that excessive lipid accumulation in tissues induces cellular damage, dysfunction, and apoptosis^40,41^. To investigate whether excessive lipid accumulation in ILC1s impairs their survival, we treated mouse ILC1s with stearic acid (SA) or palmitic acid (PA) for 24 h in vitro to induce intracellular lipid accumulation. Compared to untreated ILC1s, PA treatment inhibited the survival of liver ILC1s, and this inhibitory effect was more pronounced in ASB2-deficient ILC1s than in control ILC1s (Fig. 4a, b). Similarly, ASB2-deficient ILC1s were more susceptible to cell death induced by SA treatment than the control counterparts (Fig. 4c, d). Subsequently, we explored whether suppressing lipid overloading could reverse the survival impairment of mouse ASB2*-*deficient ILC1s. Considering that DGAT1, a key enzyme in lipogenesis^39^, is significantly up-regulated in ASB2*-*deficient ILC1s, we treated ILC1s with A922500, a specific inhibitor of DGAT1. A922500 treatment significantly reduced lipid content in both normal and ASB2-deficient ILC1s (Fig. 4e). A922500 treatment also enhanced the survival rate of ASB2-deficient ILC1s, achieving comparable survival levels to *Asb2*^fl/fl^ ILC1s (Fig. 4f). Moreover, under conditions of SA or PA treatment, A922500 treatment markedly enhanced the survival of ASB2-deficient ILC1s relative to untreated controls (Fig. 4g–j). Thus, excessive lipid accumulation caused by ASB2 deficiency induces the survival defect of mouse liver ILC1s.

### ASB2 enhances the anti-tumor activity of mouse liver ILC1s

**Fig. 5:**
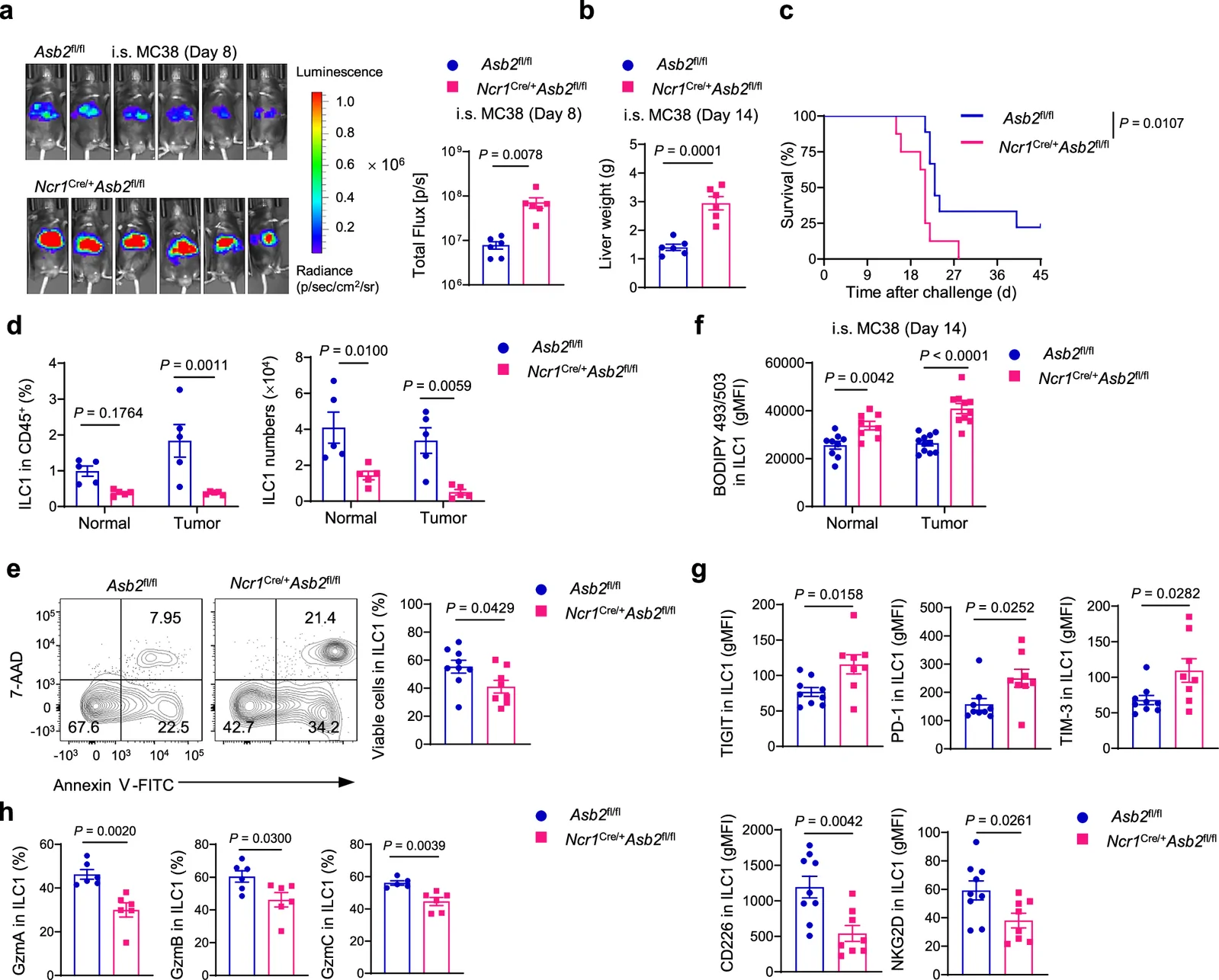
ASB2 deficiency compromises the anti-tumor function of ILC1s in mouse tumor models. **a** In vivo bioluminescence imaging (left) of CRC liver metastases and quantification (right) of the radiance values on day 8 (*n* = 6 per group; male). **b** Quantification of liver weights on day 14 (*n* = 6 per group; male). **c** Survival of *Ncr1*^Cre/+^*Asb2*^fl/fl^ (*n* = 8; male) and *Asb2*^fl/fl^ (*n* = 9; male) mice after intrasplenic injection (i.s.) of 2 × 10^5^ MC38-luc cells. **d** Frequencies and counts of ILC1s of liver ILC1s from *Ncr1*^Cre/+^*Asb2*^fl/fl^ and *Asb2*^fl/fl^ mice on day 14 after i.s. of 2 × 10^5^ MC38-luc cells (*n* = 5 per group; male). **e** Viable (Annexin Ⅴ^−^7-AAD^−^) liver ILC1s from *Ncr1*^Cre/+^*Asb2*^fl/fl^ and *Asb2*^fl/fl^ mice on day 14 after i.s. of 2 × 10^5^ MC38-luc cells (*Asb2*^fl/fl^: *n* = 9; *Ncr1*^Cre/+^*Asb2*^fl/fl^: *n* = 8; pooled from two independent experiments, with one using female mice and one using male mice). **f** BODIPY 493/503 staining of liver ILC1s from *Ncr1*^Cre/+^*Asb2*^fl/fl^ and *Asb2*^fl/fl^ mice on day 14 after i.s. of 2 × 10^5^ MC38-luc cells (*Asb2*^fl/fl^ + Normal: *n* = 9; *Ncr1*^Cre/+^*Asb2*^fl/fl^ + Normal: *n* = 8; *Asb2*^fl/fl^ + Tumor: *n* = 11; *Ncr1*^Cre/+^*Asb2*^fl/fl^ + Tumor: *n* = 10; pooled from two independent experiments with one using female mice and one using male mice). **g** Activating receptor and inhibitory receptor of liver ILC1s from *Asb2*^fl/fl^ and *Ncr1*^Cre/+^*Asb2*^fl/fl^ mice on day 14 after i.s. of 2 × 10^5^ MC38 cells (*Asb2*^fl/fl^: *n* = 9; *Ncr1*^Cre/+^*Asb2*^fl/fl^: *n* = 8; pooled from two independent experiments, with one using female mice and one using male mice). **h** GzmA (*n* = 6 per group; male), GzmB (*n* = 6 per group; male) and GzmC (*Asb2*^fl/fl^: *n* = 5; *Ncr1*^Cre/+^*Asb2*^fl/fl^: *n* = 6; male) expression of liver ILC1s from *Asb2*^fl/fl^ and *Ncr1*^Cre/+^*Asb2*^fl/fl^ mice on day 14 after i.s. of 2 × 10^5^ MC38 cells. Data are from one representative experiment of two (**c**, **h**) or three (**a**, **b**, **d**) independent experiments. Samples were compared using two-tailed unpaired Student’s *t* tests (**a**, **b**, **e**, **g**, **h**), the log-rank (Mantel-Cox) test (**c**) or two-way ANOVA (**d**, **f**) and were presented as means ± SEM. ILC1: type 1 innate lymphoid cell; GzmA/B/C: granzyme A/B/C; i.s.: intrasplenic injection; CRC: colorectal cancer.

Emerging evidence indicates that ILC1s play a role in immune surveillance against liver metastases^42,43^. Given that lipid content markedly accumulates in the tumor microenvironment during tumorigenesis and progression^41,44^, we wonder whether ASB2 participates in ILC1-mediated anti-tumor immunity. Using a mouse model of liver colorectal cancer liver metastases via intrasplenic injection of luciferase-expressing MC38 (MC38-luc) cells, we observed a significant increase in liver tumor burden in *Ncr1*^Cre/+^*Asb2*^fl/fl^ mice, as quantified by radiance values and liver weight (Fig. 5a, b). Meanwhile, compared to *Asb2*^fl/fl^ mice, *Ncr1*^Cre/+^*Asb2*^fl/fl^ mice showed significantly reduced survival (Fig. 5c). Flow cytometric analysis revealed that ASB2 deficiency resulted in a more pronounced decrease in the frequency and absolute numbers of liver ILC1s in tumor-bearing mice (Fig. 5d). Consistent with these results, the percentages of viable ILC1s (AnnexinⅤ^−^7-AAD^−^ ILC1s) were reduced in tumor-bearing *Ncr1*^Cre/+^*Asb2*^fl/fl^ mice (Fig. 5e), which was accompanied with increased lipid accumulation of ILC1s, as compared to the *Asb2*^fl/fl^ counterparts (Fig. 5f). Importantly, liver ILC1s in these tumor-bearing *Ncr1*^Cre/+^*Asb2*^fl/fl^ mice expressed higher levels of inhibitory receptors PD-1, TIGIT, and TIM-3, but lower levels of activating receptors CD226 and NKG2D, compared with their counterparts in *Asb2*^fl/fl^ mice (Fig. 5g). The expression of granzyme A (GzmA), granzyme B (GzmB), granzyme C (GzmC) in tumor-infiltrating ILC1s was also decreased in *Ncr1*^Cre/+^*Asb2*^fl/fl^ mice (Fig. 5h). However, these changes were not observed in non-tumor-bearing counterparts (Supplementary Fig. 7a, b). These results indicate that ASB2 inhibits tumor growth by promoting the anti-tumor activity of ILC1s in mouse models.

To investigate the clinical relevance, we analyzed *ASB2* expression in human normal tissues and tumors using GEPIA3. In multiple tumor types, *ASB2* expression was significantly downregulated, such as uterine carcinosarcoma (UCS), bladder urothelial carcinoma (BLCA), colon adenocarcinoma (COAD) (Supplementary Fig. 8a). Moreover, we analyzed the correlation between *ASB2* expression and the expression of ILC1 signature genes (*NCAM1*, *NCR1*, *GZMK*, *RGS1*, *ZNF683*, *ITGA1*) in normal liver, liver hepatocellular carcinoma (LIHC) peritumor, and LIHC tumor tissues, and found that *ASB2* expression was positively correlated with human ILC1 abundance (Supplementary Fig. 8b). LIHC patients with high *ASB2* expression exhibited a higher survival rate compared to those with low *ASB2* expression (Supplementary Fig. 8c). Thus, these results suggest that ASB2 expression is associated with antitumor activity of human ILC1s.

### Inhibition of lipid accumulation promotes ILC1-mediated anti-tumor immunity in mouse tumor models

**Fig. 6:**
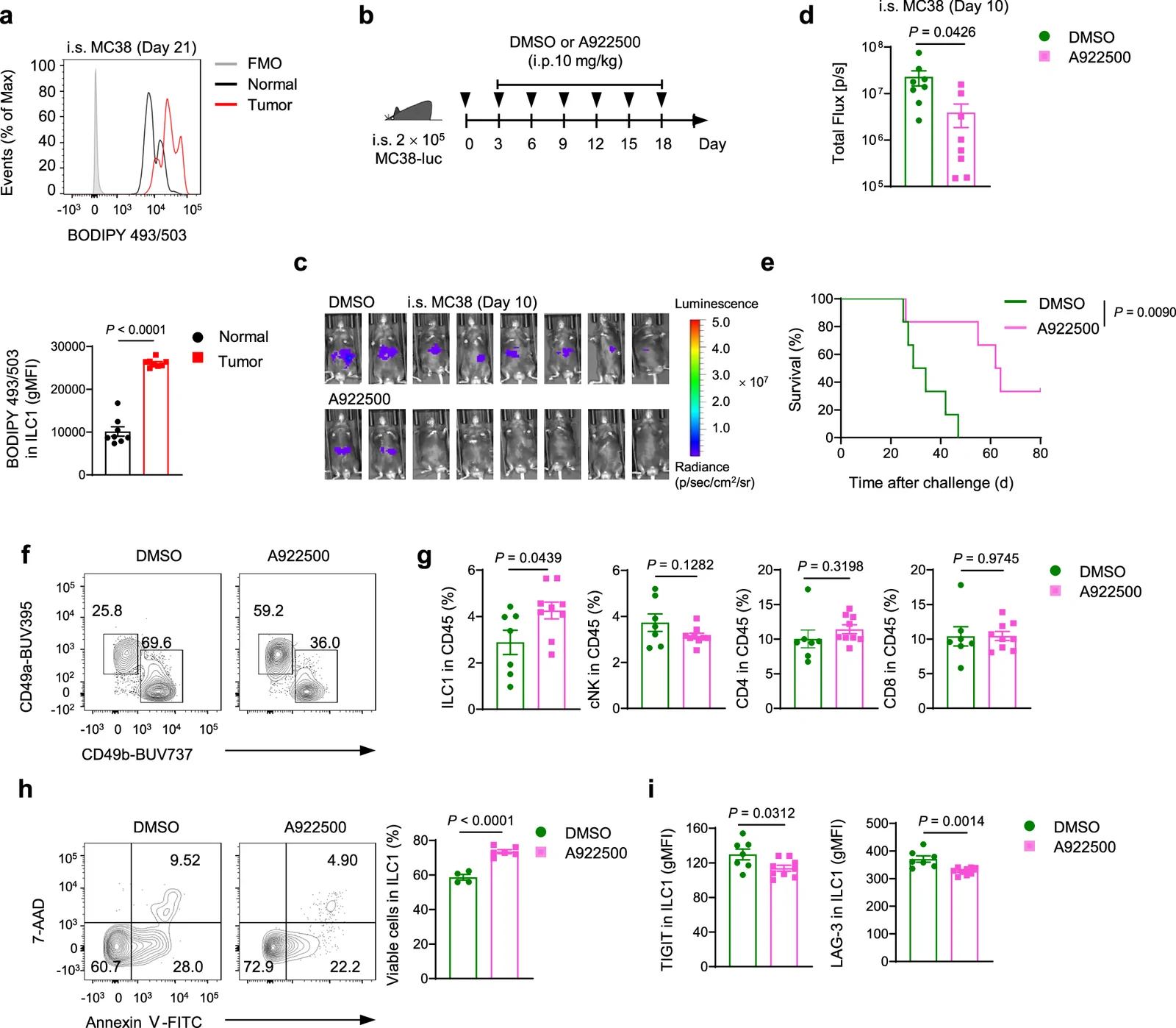
A922500 treatment relieves the progression of mouse liver metastases. **a** Flow cytometry analysis (up) and quantification (down) of BODIPY 493/503 expression in liver tumor-infiltrating ILC1s from C57BL/6 mice on day 21 after intrasplenic injection (i.s.) of 5 × 10^5^ MC38-luc cells (*n* = 8 per group; male). **b** C57BL/6 mice were intraperitoneally injected with DMSO or A922500 (10 mg/kg) every three days after initial intrasplenic injection of 2 × 10^5^ MC38-luc cells. **c** In vivo bioluminescence imaging of tumor metastases on day 10 in (**b**) (*n* = 8 per group; male). **d** Quantification of the radiance values of tumor metastases on day 10 in (**b**) (*n* = 8 per group; male). **e** Survival of C57BL/6 mice in (**b**) (*n* = 6 per group; male). **f**, **g** Representative plots (**f**) and percentages (**g**) of liver tumor-infiltrating ILC1s, cNK, CD4^+^ and CD8^+^ T cells on day 18 from C57BL/6 mice with intrasplenic injection of 2 × 10^5^ MC38-luc cells and A922500 treatment (DMSO: *n* = 7; A922500: *n* = 9; male). (**h**) Expression of viable cells (DMSO: *n* = 4; A922500: *n* = 6; male) in liver tumor-infiltrating ILC1s on day 18 from C57BL/6 mice with intrasplenic injection of 2 × 10^5^ MC38-luc cells and A922500 treatment. **i** Expression of inhibitory receptors (DMSO: *n* = 7; A922500: *n* = 9; male) in liver tumor-infiltrating ILC1s on day 18 from C57BL/6 mice with intrasplenic injection of 2 × 10^5^ MC38-luc cells and A922500 treatment. The data are all from one representative experiment of two independent experiments. Samples were compared using two-tailed unpaired Student’s *t*-tests (**a**, **d**, **g–i**) or the log-rank (Mantel-Cox) test (**e**) and were presented as means ± SEM. FMO: fluorescence minus one; ILC1: type 1 innate lymphoid cell; cNK: conventional natural killer cell; i.s.: intrasplenic injection; i.p.: intraperitoneal injection.

Compared to non-tumor-bearing mice, liver ILC1s exhibited significant lipid accumulation in mice bearing liver metastatic tumors (Fig. 6a). Considering that excessive lipid accumulation in ILC1s might impair their anti-tumor capabilities, we treated MC38-bearing mice with the DGAT1 inhibitor A922500 (Fig. 6b). DGAT1 inhibition obviously suppressed tumor growth in the liver metastasis model, as evidenced by the reduced radiance values of MC38-luc cells and prolonged survival (Fig. 6c–e). We further explored the direct effect of A922500 on the growth of MC38-luc tumors. At the dosage applied in the treatment assays involving C57BL/6 mice, A922500 did not affect tumor growth in immunodeficient NCG mice, a strain lacking ILC1s and other lymphocytes. These results demonstrate that low-dose A922500 does not directly inhibit MC38-luc tumor growth (Supplementary Fig. 9a, b). We next investigated whether inhibition of lipid accumulation in tumors could enhance ILC1-mediated anti-tumor immunity, and found that the frequency of liver ILC1s was increased in A922500-treated mice, while cNK, CD4^+^ T, and CD8^+^ T cells remained unchanged (Fig. 6f, g). Consistently, the percentages of viable ILC1s were also increased in A922500-treated tumor-bearing mice (Fig. 6h). In addition, A922500 treatment also led to lower expression of the inhibitory receptors LAG-3 and TIGIT on liver ILC1s from tumor-bearing mice (Fig. 6i). Collectively, these findings indicate that inhibition of lipid accumulation augments the anti-tumor function of mouse ILC1s, highlighting the promise of ILC1-based tumor immunotherapy.

## Discussion

Group 1 ILCs consist of cNK cells and ILC1s, both of which confer anti-virus and anti-tumor immunity in multiple tissues^5–7,42,43,45^. While cNK cells have been extensively studied, the metabolic regulation of ILC1 maintenance and functionality remains poorly understood. We find that ASB2 deficiency in group 1 ILCs in mice decreases the liver ILC1 pool size and increases ILC1 apoptosis, whereas cNK cells remain unaffected. ASB2 promotes ILC1 survival by maintaining lipid metabolic balance, and its deficiency induces lipid accumulation in ILC1s, which dampens their anti-tumor immunity. Notably, suppression of this lipid accumulation can restore ILC1-driven anti-tumor immune responses, resulting in prolonged survival of tumor-bearing mice. Thus, ASB2 is critical for maintaining mouse ILC1 homeostasis and anti-tumor function through curtailing lipid accumulation.

ASB2 has been reported to be enriched in ILCs from diverse tissues^5,11,46^. Here, our study found high expression of ASB2 in adult liver ILC1s, but not in adult cNK cells and neonatal ILC1s, under homeostatic conditions. In line with this, *Asb2* ablation in NKp46^+^ cells selectively reduced adult ILC1 numbers without affecting homeostasis of adult cNK cells and early-life ILC1s. Mechanistically, our proteomics data showed that the expression level of DGAT1 was significantly up-regulated in mouse ASB2-deficient ILC1s. Co-IP assays further verified a physical association between ASB2 and DGAT1. DGAT1 is a critical enzyme in triglyceride synthesis and lipid droplet biogenesis. Using A922500, the inhibitor of DGAT1, could reverse ILC1 survival defects caused by ASB2-deficiency. These findings indicate that ASB2 regulates mouse ILC1s' survival through a mechanism involving DGAT1. However, it is important to acknowledge the limitations of the mechanistic studies presented here. It remains unclear whether the upregulation of DGAT1 following *Asb2* deficiency is directly mediated by ASB2 or occurs through an indirect transcriptional or post-translational mechanism. Particularly, whether this effect depends on the putative ubiquitin ligase activity of ASB2 requires further investigation. Moreover, detailed molecular interaction analyses in bona fide ILC1s remain technically challenging at present. Therefore, the precise regulatory mechanisms between ASB2 and DGAT1 warrant further investigation in future studies.

Recent studies have revealed critical roles of lipid metabolism in cNK cell function. Optimal effector functions of human cNK cells require a baseline level of neutral lipids and active lipid metabolism^15^. Furthermore, ascites from patients with HGSOC reduced the normal levels of lipid droplets in cNK cells and impaired their anti-tumor functions^20^. Paradoxically, however, elevated levels of lipids can be detrimental to the function of cNK cells. In the context of tumor or obesity, excessive lipid accumulation in cNK cells impairs their activation and anti-tumor functions^16–19^. These reports suggest that lipid metabolism acts as a double-edged sword that regulates cNK cell homeostasis and functions. Our proteomics and transcriptomic analysis demonstrated that ASB2 deficiency significantly up-regulated lipid metabolic pathways in mouse ILC1s. Using BODIPY493/503 staining, we found pronounced neutral lipid accumulation in ILC1s of *Ncr1*^Cre/*+*^*Asb2*^fl/fl^ mice. Although our pharmacological perturbation experiments using A922500 indicate that excessive lipid accumulation compromises ILC1 viability, it is crucial to acknowledge the nature of lipids as essential cellular nutrients. We postulate that insufficient lipid levels may also be detrimental to ILC1s, and there is a potentially beneficial zone where balanced lipid homeostasis optimizes ILC1 fitness. Future studies should quantitatively define this optimal lipid threshold through metabolic profiling coupled with functional assays, which may reveal strategies to fine-tune lipid metabolism in ILC1s.

Filamin proteins are well-established substrates targeted for ubiquitination and degradation by ASB2. Mounting evidence has demonstrated the role of the ASB2-filamin axis in regulating cytoskeleton remodeling, cell adhesion, and cell migration in various immune cell types^26,27,47^. For instance, ASB2 has been reported to regulate the migration of DCs, Th2 and NK cells by modulating filamin levels^26,27,48^. Consistently, our study revealed that ASB2 deficiency altered the expression of genes associated with the negative regulation of the cell adhesion pathway, and upregulated the expression of Filamin A (FLNA) and Filamin C (FLNC). While the present work focused on ASB2 in governing ILC1 lipid metabolism via DGAT1, whether ASB2 directly alters ILC1 migration warrants further investigation.

Aberrant lipid enrichment is a hallmark of the tumor microenvironment (TME), creating a lipid-laden niche that fuels immunosuppression. Lipids in the TME impair NK and T cell function by reducing the secretion of apoptosis-inducing enzymes and cytokines^19,49^. They also suppress DCs activity, promote myeloid-derived suppressor cell (MDSC) infiltration, and enhance Treg cell activity, all of which hinder anti-tumor immunity^50–52^. Within the TME, we also observed lipid overload in ILC1s. This lipogenic burden was exacerbated in *Ncr1*^Cre/*+*^*Asb2*^fl/fl^ mice, concomitant with survival and functional defects of ILC1s, as well as accelerated tumor progression. These findings suggest ASB2 as a guardian against TME-induced lipotoxicity, preserving ILC1-mediated tumor surveillance in mouse tumor models. However, a potential limitation of our study is that *Ncr1*^Cre/+^-mediated deletion of *Asb2* may also affect cNK cells. Although cNK cells lack *Asb2* expression at steady state, the TME might drive their differentiation towards an ILC1-like or tissue-resident program^53^. Therefore, we cannot completely exclude the contribution of cNK cells to the observed phenotypes in tumor models. Nonetheless, our data strongly support a critical role of ASB2 in ILC1s in protecting against TME lipotoxicity and preserving anti-tumor immunity. Inhibiting lipogenesis with A922500 significantly delayed tumor progression and prolonged the survival of MC38 tumor-bearing mice. Given the broad suppression of immune cells by TME lipids, this treatment likely exerts pleiotropic effects beyond enhancing ILC1 activities by reversing lipid overload-mediated immunosuppression of multi-lineage immune cells.

Collectively, our study unveils that ASB2 preserves mouse liver ILC1 fitness through balancing lipid metabolism. Importantly, ASB2 promotes ILC1-mediated anti-tumor immunity in a mouse model of liver metastasis, and targeting lipid accumulation can unleash ILC1 immunosurveillance in the TME, thus providing a strategy for liver tumor therapy.

## Methods

### Mice and cell lines

C57BL/6 mice (Cat. NO. N000013) and NCG mice (Cat. NO. T001475) were purchased from GemPharmatech (Nanjing, China). *Ncr1*^Cre/+^ mice were generously provided by Eric Vivier (Aix Marseille Université, Marseille, France)^54,55^. *Vav*^Cre/+^ mice were generously provided by Zhongjun Dong (Tsing Hua University, Beijing, China)^43,56^. *Ncr1*^CreERT2/+^ mice (Cat. NO. NM-KI-200004) were obtained from Shanghai Model Organisms Center (Shanghai, China). *Asb2*^fl/fl^ mice (Cat. NO. S-CKO-12815) were purchased from Cyagen (Suzhou, China). The *Asb2* gene is located on the mouse chromosome 12. Ten exons are identified, with the ATG start codon in exon 2 and the TAA stop codon in exon 10. Exon 4 is selected as the conditional knockout region. The region contains a 167 bp coding sequence. Deletion of this region results in the loss of function of the mouse *Asb2* gene. *Asb2*^EGFP^ mice were generated by the Laboratory Animal Center of the University of Science and Technology of China (USTC). *Asb2α* and *Asb2β* share no common N-terminus but have a conserved C-terminus, and the EGFP knock-in strategy was designed to target the C-terminus of *Asb2*, immediately upstream of the stop codon TAA. For proteomic and RNA-seq profiling, ILC1s were purified from a pool of multiple adult mice, including both females and males. For other experiments of adult mice, age- (6–14 weeks old) and sex-matched mice were used within each individual experiment to minimize biological variation, and independent replicate experiments were performed using either male or female mice independently. Sex was not distinguished in E17.5 embryonic mice and neonatal mice. This study primarily focuses on the regulatory mechanism of ASB2 in mouse liver ILC1s. To date, no published studies have reported sex bias of liver ILC1s; therefore, we did not conduct sex-based comparative analyses. All mice were housed in a specific-pathogen-free (SPF) facility, adhering to the experimental animal care standards set by the University of Science and Technology of China. All mice were housed in animal facilities on a 12 h dark/light cycle with an ambient temperature of 20–26 °C and relative humidity of 40–70%. *Asb2*^fl/fl^ mice and *Ncr1*^Cre/+^*Asb2*^fl/fl^ mice or *Vav*^Cre/+^*Asb2*^fl/fl^ were co-housed. *Ncr1*^CreERT2/+^ and *Asb2*^fl/fl^*Ncr1*^CreERT2/+^ mice were bred separately. *Ncr1*^Cre/+^ and *Ncr1*^Cre/+^*Asb2*^fl/fl^ mice were bred separately. All of the mouse experiments were approved by the Ethics Committee of the University of Science and Technology of China, and the protocol number is USTCACUC26070122077. The MC38 cell line was kindly provided by Prof Yang-xin Fu (Tsing Hua University, Beijing, China)^57^, and the HEK-293T cell line was obtained from the Cell Bank of the Type Culture Collection of the Chinese Academy of Sciences (Shanghai, China). All cell lines were tested mycoplasma negative.

### Cell isolation

Liver tissues were homogenized by passing through a 200-gauge mesh, and liver MNCs were purified via Percoll (Cytiva, Cat. NO. 17089109) density gradient centrifugation (40% and 70%). Splenocytes were isolated by pressing excised spleens through a 200-gauge mesh, followed by red blood cell lysis with red cell lysis buffer (Solarbio, Cat. NO. R1010). Lung tissues were minced into small fragments and enzymatically digested in RPMI 1640 medium (VivaCell, Cat. NO.C3010-0500) containing 0.1% collagenase I (Sigma, Cat. NO. C0130) at 37 °C for 40 min with constant agitation. The digested suspension was then centrifuged over a Percoll gradient for cell purification. The mesentery of the small intestine was carefully excised and gently rinsed to remove luminal contents. The intestines were cut into pieces and incubated in 20 ml Extraction buffer supplemented with 5% FBS (Gibco, Cat. NO. 10091-148), 5 mM EDTA (Sangon Biotech, Cat. NO. B540625), and 15 mM HEPES and rotated at 200 rpm for 30 min at 37 °C twice. The intestine was washed, minced, and digested with DMEM (VivaCell, Cat. NO. C3113-0500) containing 10% FBS, 0.1% collagenase IV (Sigma, Cat. NO. C5138) and 0.25 mg/ml DNase I (Sigma, Cat. NO. D5025) with shaking at 37 °C for 1 h. The supernatants were filtered through a 200-gauge steel mesh, and IELs were collected after Percoll gradient centrifugation.

### Colorectal cancer liver metastases model and A922500 treatment

To induce liver metastases, mice were injected intrasplenically with 2 × 10^5^ or 5 × 10^5^ MC38-luc cells, followed by splenectomy 4 min after injections. Mice were euthanized 14 days after the initial injection or investigated for survival. For A922500 (MedChemExpress, Cat. NO. HY-10038) treatment, C57BL/6 mice were randomly assigned to a control group and a treatment group and received intraperitoneal injection five or six times with 250 μg (10 mg/kg) A922500 or DMSO (Solarbio, Cat. NO. D8371) control once every 3 days from day 3. For the metastatic MC38-luc model, we monitored the health status of mice daily. The criteria for early endpoints included weight loss > 20%, lethargy, hunched posture and abdominal distension. Euthanasia was performed by carbon dioxide inhalation immediately upon reaching any of these criteria.

### Antibody staining and flow cytometry

The isolated cells were incubated with rat serum at 4 °C for 30 min to block Fc receptors, followed by staining with fluorescently labeled mAbs (Supplementary Table 1) against surface molecules. For intracellular cytokine staining, cells were stimulated with PMA (30 ng/mL, MilliporeSigma, Cat. NO. P1585), Ionomycin (1 µg/mL, MilliporeSigma, Cat. NO. I0634-5 mg) for 4 h, and monensin (2 µg/mL, MilliporeSigma, Cat. NO. M5273-500mg) was added at the beginning of the stimulation procedure. CD107a was stained during stimulation. After surface staining, the cells were fixed, permeabilized (Invitrogen, Cat. NO. 00-5523-00), and stained with mAbs against intracellular molecules. For neutral lipid staining, cells were stained using BODIPY 493/503 (500 ng/mL, Thermo Fisher, Cat. NO. D3922) at 37 °C for 15–20 min. All data were collected using a flow cytometer (X20, LSRFortessa; BD Biosciences) and analyzed with FlowJo software (TreeStar).

### In vivo treatment

*Ncr1*^CreERT2/+^*Asb2*^fl/fl^ and *Ncr1*^CreERT2/+^mice were intraperitoneally injected with tamoxifen (2 mg, i.p.; MedChemExpress, Cat. NO. 10540-29-1) dissolved in corn oil (200 μL, Solarbio, Cat. NO. C7030) for five days and flow cytometry analysis was performed after 21 days.

### In vitro cell culture

Liver MNC suspensions were all cultured in 1640 RPMI supplemented with 10% FBS, 100 U/mL penicillin, 0.1 mg/mL streptomycin (Beyotime, Cat. NO. C0222), 50 μM *β*-mercaptoethanol (Gibco, Cat. NO. 21985-023) and 10 ng/mL mouse interleukin-15 (IL-15; Peprotech, Cat. NO. 210-15-10 μg), moreover 100 μM PA (Shanghai Siduorui Biotechnology, Cat. NO. PA01)/ 50 μM SA (Shanghai Siduorui Biotechnology, Cat. NO. SA01)/ 3 μM A922500 were added according to experimental design for 24 h. The cells were then subjected to cell viability assay or lipid quantification.

### Lipid quantification

Cells were washed twice with PBS and incubated in serum-free PBS for 1 h at 37°C in a 5% CO_2_ incubator to induce mild starvation before lipid content detection. For measuring uptake of fatty acids, cells were incubated in PBS containing 0.5 μg/mL BODIPY 500/510 C1, C12 (ThermoFisher, Cat. NO. D3823), or PBS containing 0.05 μg/mL BODIPY FL C16 (ThermoFisher, Cat. NO. D3821). For neutral lipid staining, cells were performed using 0.5 μg/mL BODIPY 493/503 at 37 °C for 15-20 min. For the detection of intracellular lipid content, cultured cells were fixed, permeabilized, and stained with 0.5 μg/mL BODIPY 500/510 C1, C12, 0.05 μg/mL BODIPY FL C16, 0.5 μg/mL BODIPY 493/503 and 10 μM NBD Cholesterol (ThermoFisher, Cat. NO. N1148) at 37 °C for 15–20 min.

### scRNA-seq cell clustering

The fetal and adult liver group 1 ILCs scRNA-seq datasets (GSE202475) were downloaded from online websites^28^ (https://www.ncbi.nlm.nih.gov/geo/query/acc.cgi?acc=GSE202475). The liver group 1 ILCs dataset was preprocessed for quality control and removal of outlier cells according to the following criteria: feature RNA between 750 and 7000 and mitochondrial counts of <7%. Group 1 ILCs were clustered by the “Seurat” plugin of R 4.3 (R Institute for Statistical Computing, Vienna, Austria). Uniform Manifold Approximation and Projection (UMAP) dimensional reduction was generated based on selected principal components. Selected genes are displayed based on their mean expression.

### RT-qPCR

Total RNA from liver ILC1s and cNK cells was extracted using TRIzol (ThermoFisher, Cat. NO. 15596018). Specifically, the samples were homogenized in TRIzol reagent, followed by the addition of chloroform. After centrifugation, the homogenate was separated into three distinct phases: a clear upper aqueous phase containing RNA, an intermediate interphase, and a pink lower organic phase containing DNA and proteins. RNA was then precipitated from the upper aqueous phase using isopropanol. RNA was applied to generate cDNA using M-MLV Reverse Transcriptase for qPCR according to the protocol (Thermo Fisher Scientific, Cat. NO. 2382833). qPCR was performed using SYBR Green *Pro Taq* HS premix (Accurate Biology, Cat. NO. AG11702) in a LightCycler 96 (Roche). Gene expression was analyzed using the 2^-ΔΔCT^ method. All transcriptional levels were normalized to the housekeeping gene *β-actin*, and data are presented as fold changes relative to control. Primers were synthesized by Sangon Biotech (Shanghai, China) and the sequences are listed in Supplementary Table 2.

### Co-IP assay

Mouse *Asb2α*, *Asb2β* and *Dgat1* genes were synthesized by GENEWIZ (Suzhou, China) and cloned into pcDNA3.1-MYC and pcDNA3.1-Flag vectors. pcDNA3.1 vectors were kindly provided by Prof Shu Zhu (University of Science and Technology of China, Hefei, China)^58^. For exogenous co-immunoprecipitation assays, HEK 293 T cells were seeded in 6-well plates and transfected with the indicated plasmids using polyethylenimine. After 24 h of incubation, cells were harvested and lysed in ice-cold IP lysis buffer supplemented with a complete protease inhibitor cocktail (MedChemExpress, Cat. NO. HY-K0010) and phenylmethylsulfonyl fluoride (BOSTER, Cat. NO. AR1192). Whole-cell extracts were then incubated and immunoprecipitated with Flag-M2 monoclonal antibody–conjugated agarose beads (Sigma, Cat. NO. A2220). The immunoprecipitates were finally analyzed by immunoblotting. Antibody information is listed in Supplementary Table 3. Uncropped and unprocessed scans of the blots are in the Source Data file.

### RNA-seq and transcriptomic analysis

Liver ILC1s (CD45^+^NK1.1^+^CD3^−^CD19^−^CD49a^+^CD49b^−^) from *Asb2*^fl/fl^ and *Asb2*^fl/fl^*Ncr1*^Cre/+^ mice were sorted for bulk RNA-seq, and all cells were dissolved in the reagent TRIzol Reagent. UID RNA-seq experiment and high-throughput sequencing and data analysis were conducted by Seqhealth Technology Co., LTD (Wuhan, China).

Raw sequencing data were filtered by Trimmomatic (version 0.36), low-quality reads were discarded, and the reads contaminated with adapter sequences were trimmed. Clean Reads were further treated with in-house scripts to eliminate duplication bias introduced in library preparation and sequencing. In brief, clean reads were clustered according to the UMI sequences, in which reads with the same UMI sequence were grouped into the same cluster. Reads in the same cluster were compared to each other by pairwise alignment, and then reads with sequence identity over 95% were extracted to a new sub-cluster. After all sub-clusters were generated, multiple sequence alignment was performed to get one consensus sequence for each sub-cluster. After these steps, any errors and biases introduced by PCR amplification or sequencing were eliminated. The de-duplicated consensus sequences were used for standard RNA-seq analysis. They were mapped to the reference genome of the mouse using STAR software (version 2.5.3a) with default parameters. Reads mapped to the exon regions of each gene were counted by feature Counts (Subread-1.5.1; Bioconductor), and then RPKM was calculated. Differentially expressed genes (DEGs) between groups were identified using the DESeq2 package (version 4.3.1). A significance threshold of *p* < 0.05 and a fold-change cutoff of 1.5 were applied to determine statistically significant gene expression differences. Gene ontology analysis was performed using the Gene Ontology database with up-regulated DEGs as input, with a P-value cutoff of 0.05 to judge statistically significant enrichment (version 4.3.1).

RNA-seq for liver ILC1s isolated from *Asb2*^fl/fl^ and *Asb2* conditional knockout have been uploaded to the Genome Sequence Archive CRA030972.

### Microarray data analysis

The liver CD49b^−^ ILC1, liver CD49b^+^ cNK, small intestine ILC1, small intestine ILC2, small intestine NKp46^−^CD4^+^ ILC3, small intestine NKp46^+^ ILC3 and small intestine CD127^−^ cNK datasets (GSE37448)^35^ were downloaded from online websites (https://ncbi.nlm.nih.gov/geo/query/acc.cgi?acc=GSE37448). Differentially expressed genes (DEGs) between groups were identified using the limma package (version 4.3.1).

### Nano-LC-MS/MS

Liver ILC1s (CD45^+^NK1.1^+^CD3^−^CD19^−^CD49a^+^CD49b^−^) from *Asb2*^fl/fl^ and *Asb2*^fl/fl^*Ncr1*^Cre/+^ mice were collected. Protein extraction, tryptic digestion and Nano-LC-MS/MS for liver ILC1s were performed by iProteome in China.

For the proteome profiling samples, peptides were analyzed on a Q Exactive HF-X Hybrid Quadrupole-Orbitrap Mass Spectrometer (Thermo Fisher Scientific) coupled with a high-performance liquid chromatography system (EASY nLC 1200, Thermo Fisher Scientific). Dried peptide samples re-dissolved in Solvent A (0.1% formic acid in water) were loaded onto a 2-cm self-packed trap column using Solvent A and separated on a 150-μm-inner-diameter column. Eluted peptides were ionized and introduced into the mass spectrometer. Mass spectrometry was performed in data-dependent acquisition mode.

For the proteomics data, FOTs multiplied by 1 × 10⁵ were used for quantification, and missing values were imputed with one-tenth of the minimum value and finally, log2 transformed, if necessary.

Conduct qualitative and quantitative analysis of identified proteins, including overall differential protein analysis and screening of differentially expressed proteins. A volcano plot is used to visualize differentially expressed proteins between two sample groups. It employs two key metrics: fold change (FC = 1.5) and the statistical *p*-value (*p* < 0.05) from hypothesis testing to screen for significantly altered proteins. Gene ontology analysis was performed with up-regulated DEPs as input. Proteome data for liver ILC1s isolated from *Asb2*^fl/fl^ and *Asb2* conditional knockout mice have been uploaded to the ProteomeXchange Consortium via the iProX partner repository PXD069231 (https://www.iprox.cn/page/project.html?id=IPX0013726000).

### Statistical analysis

Statistical analyses were performed using GraphPad Prism version 9 (GraphPad, San Diego, CA, USA). Data were presented as the mean ± SEM. Statistical differences were evaluated using a two-tailed unpaired Student *t*-test, one-way ANOVA and two-way ANOVA. Survival data were analyzed using a log-rank test.

### Reporting summary

Further information on research design is available in the Nature Portfolio Reporting Summary linked to this article.

## Supplementary information

Supplementary information

Description of Supplementary Files

Supplementary data1

Supplementary data2

Supplementary data3

Supplementary data4

Reporting Summary

Transparent Peer Review file

## Source data

Source data1

## Data Availability

The RNA-seq data generated in this study have been deposited in the China National Center for Bioinformation (CNCB) under accession code CRA030972; The Proteome data generated in this study have been deposited in the ProteomeXchange Consortium via the iProX partner repository under accession code PXD069231 (https://www.iprox.cn//page/project.html?id=IPX0013726000). scRNA-seq and microarray data from publicly accessible datasets were retrieved from GSE202475^28^ (https://www.ncbi.nlm.nih.gov/geo/query/acc.cgi?acc=GSE202475) and GSE37448^35^ (https://ncbi.nlm.nih.gov/geo/query/acc.cgi?acc=GSE37448). All data are included in the Supplementary Information or available from the authors, as are unique reagents used in this Article. The raw numbers for charts and graphs are available in the Source Data file whenever possible. Source data are provided with this paper.
