# Supplementary material for: ASB2 inhibits lipid accumulation to promote ILC1 homeostatic fitness and anti-tumor immunity in the mouse liver
Source: Nat Commun. 2026 Jul 17;17:8741. doi: 10.1038/s41467-026-75517-4 (PMC13493952; doi:10.1038/s41467-026-75517-4)
Supplement: Supplementary file 1 [file 41467_2026_75517_MOESM1_ESM.pdf]

Supplementary information

ASB2 inhibits lipid accumulation to promote ILC1 homeostatic fitness and anti-tumor immunity in the mouse liver

Boqun Bao, Xianwei Wang*, Zhigang Tian*, Hui Peng*

*Corresponding authors. Email: wangxianwei@ustc.edu.cn (X.W.); tzg@ustc.edu.cn (Z.T.); huipeng@ustc.edu.cn (H.P.).

The PDF file includes:
Supplementary Figure 1-9.
Supplementary Table 1-3

All other Supplementary Data (Supplementary Data 1-4) are provided in the file titled "Description of Supplementary files".

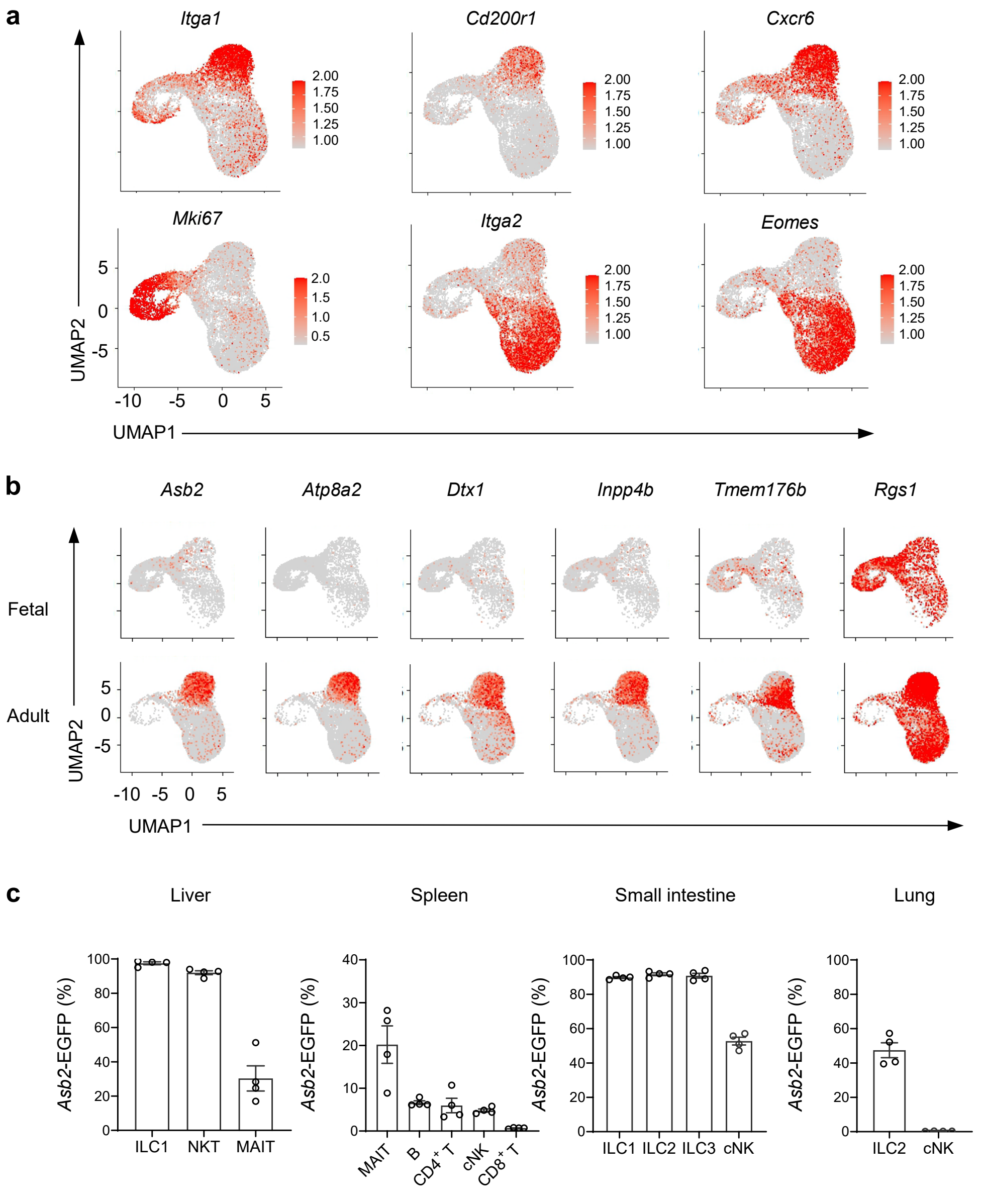

Supplementary Figure 1. Genetic characteristics of fetal and adult liver group 1 ILCs subpopulations. (a) Distribution of signature gene expression by the liver group 1 ILCs clusters in UMAP plots. Raw data were extracted from GSE202475. **(b)** Feature plots of relative expression levels of *Asb2*, *Atp8a2*, *Dtx1*, *Inpp4b*, *Tmem176b*, and *Rgs1* in each cluster. **(c)** *Asb2* expression in various lymphocyte populations across liver (*n* = 4 per group; female), spleen (*n* = 4 per group; female), small intestine (*n* = 4 per group; male) and lung (*n* = 4 per group; male) of adult *Asb2*^{EGFP} mice by flow cytometric analysis. Data are from one representative experiment of two independent experiments. Samples were presented as means ± SEM. ILC1: type 1 innate lymphoid cell; cNK: conventional natural killer cell; MAIT: mucosal-associated invariant T cell; NKT: natural killer T cell; ILC2: type 2 innate lymphoid cell; ILC3: type 3 innate lymphoid cell.

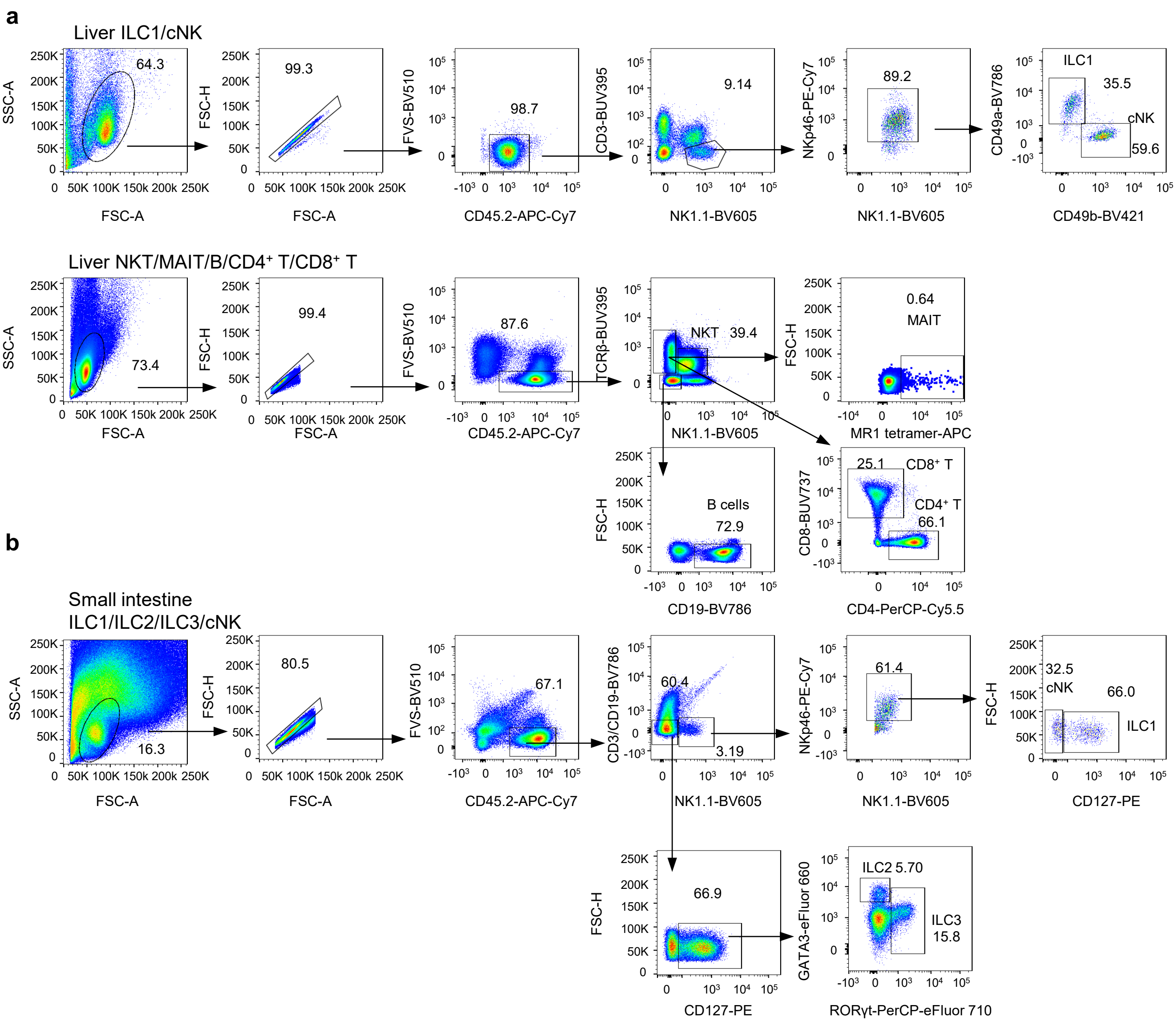

Supplementary Figure 2. The gating strategy of different lymphoid cells in liver and small intestine. (a) Representative plots showing the gating strategy of NKT cells (CD45.2$^+$FVS$^-$NK1.1$^+$TCRβ$^+$), MAIT cells (CD45.2$^+$FVS$^-$NK1.1$^-$TCRβ$^+$MR1 tetramer$^+$), B cells (CD45.2$^+$FVS$^-$NK1.1$^-$TCRβ$^-$CD19$^+$) , CD4$^+$ T cells (CD45.2$^+$FVS$^-$NK1.1$^-$TCRβ$^+$CD4$^+$CD8$^-$), CD8$^+$ T cells (CD45.2$^+$FVS$^-$NK1.1$^-$TCRβ$^+$CD4$^-$CD8$^+$), ILC1s (CD45.2$^+$FVS$^-$NK1.1$^+$CD3$^-$NKp46$^+$CD49b$^-$CD49a$^+$), and cNK cells (CD45.2$^+$FVS$^-$NK1.1$^+$CD3$^-$NKp46$^+$ CD49b$^+$CD49a$^-$) in the liver from *Asb2*$^{fl/fl}$, *Asb2*^{EGFP}, *Ncr1*$^{Cre/+}$*Asb2*$^{fl/fl}$, *Vav*$^{Cre/+}$*Asb2*$^{fl/fl}$, *Ncr1*$^{Cre/+}$, *Ncr1*$^{CreERT2/+}$, *Ncr1*$^{CreERT2/+}$*Asb2*$^{fl/fl}$ and C57BL/6 mice presented on Figure 1d, 1j, 1k, 2a-h, 3d-f, 4a-j, 5d-g, 6a, 6f-i and Supplementary Figure 1c, 4c, 4d, 5a, 5c, 5d, 6d, 6e, 7a, 7b. **(b)** Representative plots showing the gating strategy of ILC1s (CD45.2$^+$FVS$^-$NK1.1$^+$CD19$^-$CD3$^-$NKp46$^+$CD127$^+$) , cNK (CD45.2$^+$FVS$^-$NK1.1$^+$CD19$^-$CD3$^-$NKp46$^+$CD127$^-$), ILC2s (CD45.2$^+$FVS$^-$NK1.1$^-$CD19$^-$CD3$^-$CD127$^+$GATA3$^+$) and ILC3s (CD45.2$^+$FVS$^-$NK1.1$^-$CD19$^-$CD3$^-$CD127$^+$RORγt$^+$) in the small intestine from *Asb2*$^{fl/fl}$, *Asb2*^{EGFP} and *Vav*$^{Cre/+}$*Asb2*$^{fl/fl}$ mice presented on Supplementary Figure 1c, 4d. ILC1: type 1 innate lymphoid cell; cNK: conventional natural killer cell; MAIT: mucosal-associated invariant T cells; NKT: natural killer T cell; ILC2: type 2 innate lymphoid cell; ILC3: type 3 innate lymphoid cell.

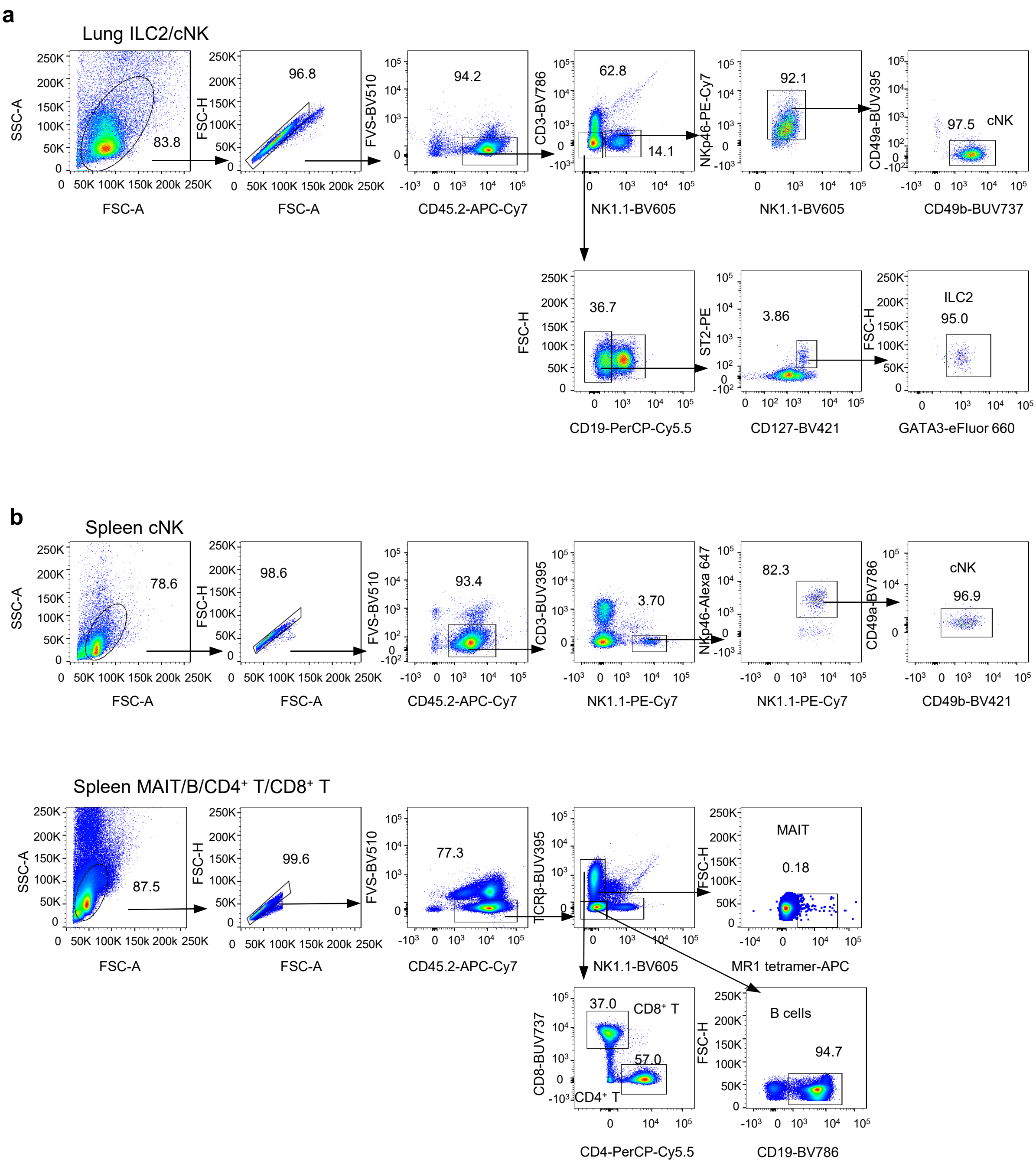

Supplementary Figure 3. The gating strategy of different lymphoid cells in lung and spleen. (a) Representative plots showing the gating strategy of ILC2s ($CD45.2^+FVS^-NK1.1^-CD19^-CD3^-CD127^+ST2^+GATA3^+$) and cNK ($CD45.2^+FVS^-NK1.1^+CD3^-NKp46^+CD49b^+CD49a^-$) in the lung from *Asb2*$^{fl/fl}$, *Asb2*^{EGFP} and *Vav*$^{Cre/+}$*Asb2*$^{fl/fl}$ mice presented on Supplementary Figure 1c, 4d. **(b)** Representative plots showing the gating strategy of MAIT cells ($CD45.2^+FVS^-NK1.1^-TCR\beta^+$MR1 tetramer$^+$), B cells ($CD45.2^+FVS^-NK1.1^-TCR\beta^-CD19^+$) , $CD4^+$ T cells ($CD45.2^+FVS^-NK1.1^-TCR\beta^+CD4^+CD8^-$), $CD8^+$ T cells ($CD45.2^+FVS^-NK1.1^-TCR\beta^+CD4^-CD8^+$), ILC1s ($CD45.2^+FVS^-NK1.1^+CD3^-NKp46^+CD49b^-CD49a^+$), and cNK cells ($CD45.2^+FVS^-NK1.1^+CD3^-NKp46^+ CD49b^+CD49a^-$) in the spleen from *Asb2*$^{fl/fl}$, *Asb2*^{EGFP}, *Ncr1*$^{Cre/+}$*Asb2*$^{fl/fl}$ and *Ncr1*$^{Cre/+}$ mice presented on Supplementary Figure 1c, 4b, 5b. cNK: conventional natural killer cell; MAIT: mucosal-associated invariant T cells; ILC2: type 2 innate lymphoid cell.

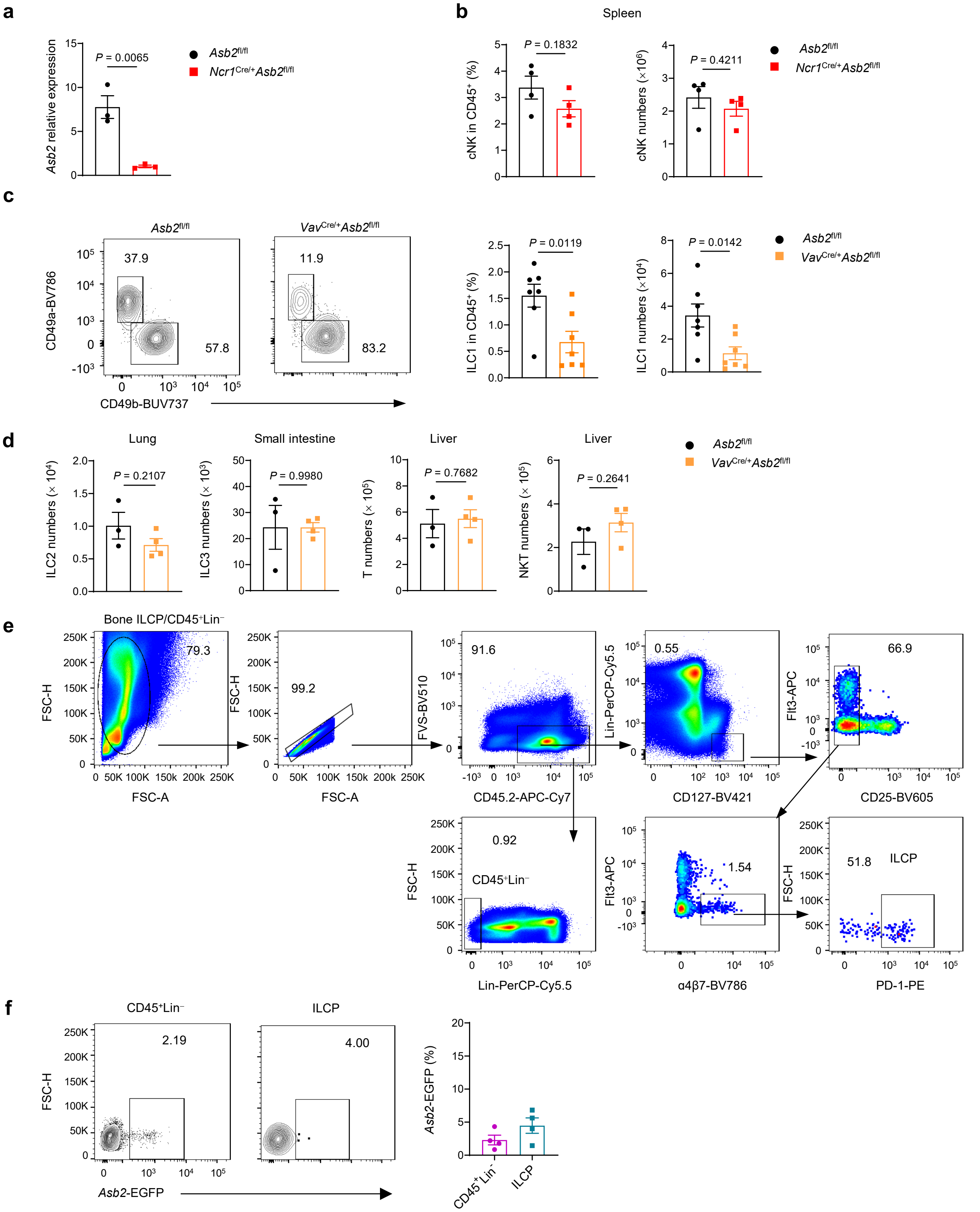

Supplementary Figure 4. ASB2 is dispensable for the development and differentiation of ILCs and the homeostasis of NKT, ILC2 and ILC3 subsets (a) Quantitative PCR analysis of *Asb2* expression in liver NK1.1$^+$NKp46$^+$ cells from male *Ncr1*$^{Cre/+}$*Asb2*$^{fl/fl}$ and *Asb2*$^{fl/fl}$ mice (n = 3 per group; male). Gene expression is normalized to that of housekeeping gene *β-actin* and is presented relative to that of *Asb2*-deficient NK1.1$^+$NKp46$^+$ cells . **(b)** Frequencies and absolute numbers of spleen cNK cells from adult *Ncr1*$^{Cre/+}$*Asb2*$^{fl/fl}$ and *Asb2*$^{fl/fl}$ mice (n = 4 per group; male). **(c)** Representative plots (left) and percentages and absolute numbers (right) of liver ILC1s from *Vav*$^{Cre/+}$*Asb2*$^{fl/fl}$ and *Asb2*$^{fl/fl}$ mice (n = 7 per group; pooled from two independent experiment with one using female mice and one using male mice). **(d)** Numbers of lung ILC2s, small intestine ILC3s, liver NKT and liver T cells from *Vav*$^{Cre/+}$*Asb2*$^{fl/fl}$ (n = 4; male) and *Asb2*$^{fl/fl}$ mice (n = 3; male). **(e)** Representative plots showing the gating strategy and characteristics of CD45.2$^+$Lin$^-$ (Lin$^-$: NK1.1$^-$CD11b$^-$CD11c$^-$CD19$^-$CD3$^-$Gr1$^-$Ter119$^-$) cells and ILCPs (CD45.2$^+$Lin$^-$CD127$^+$CD25$^-$Flt3$^-$α4β7$^+$ PD-1$^+$) in the bone marrow from *Asb2*^{EGFP} mice presented on Supplementary Figure 4f. **(f)** Flow cytometry analysis (left) and quantification (right) of *Asb2* expression in CD45$^+$Lin$^-$ cells and ILCPs in the bone marrow of adult *Asb2*^{EGFP} mice. (n = 4 per group; female). Data are from one representative experiment of two independent experiments **(a, b, d-f)**. Samples were all compared using two-tailed unpaired Student's t tests and were presented as means ± SEM. ILC1: type 1 innate lymphoid cell; cNK: conventional natural killer cell; MAIT: mucosal-associated invariant T cells; NKT: natural killer T cell; ILC2: type 2 innate lymphoid cell; ILC3: type 3 innate lymphoid cell; ILCP: innate lymphoid cell progenitor.

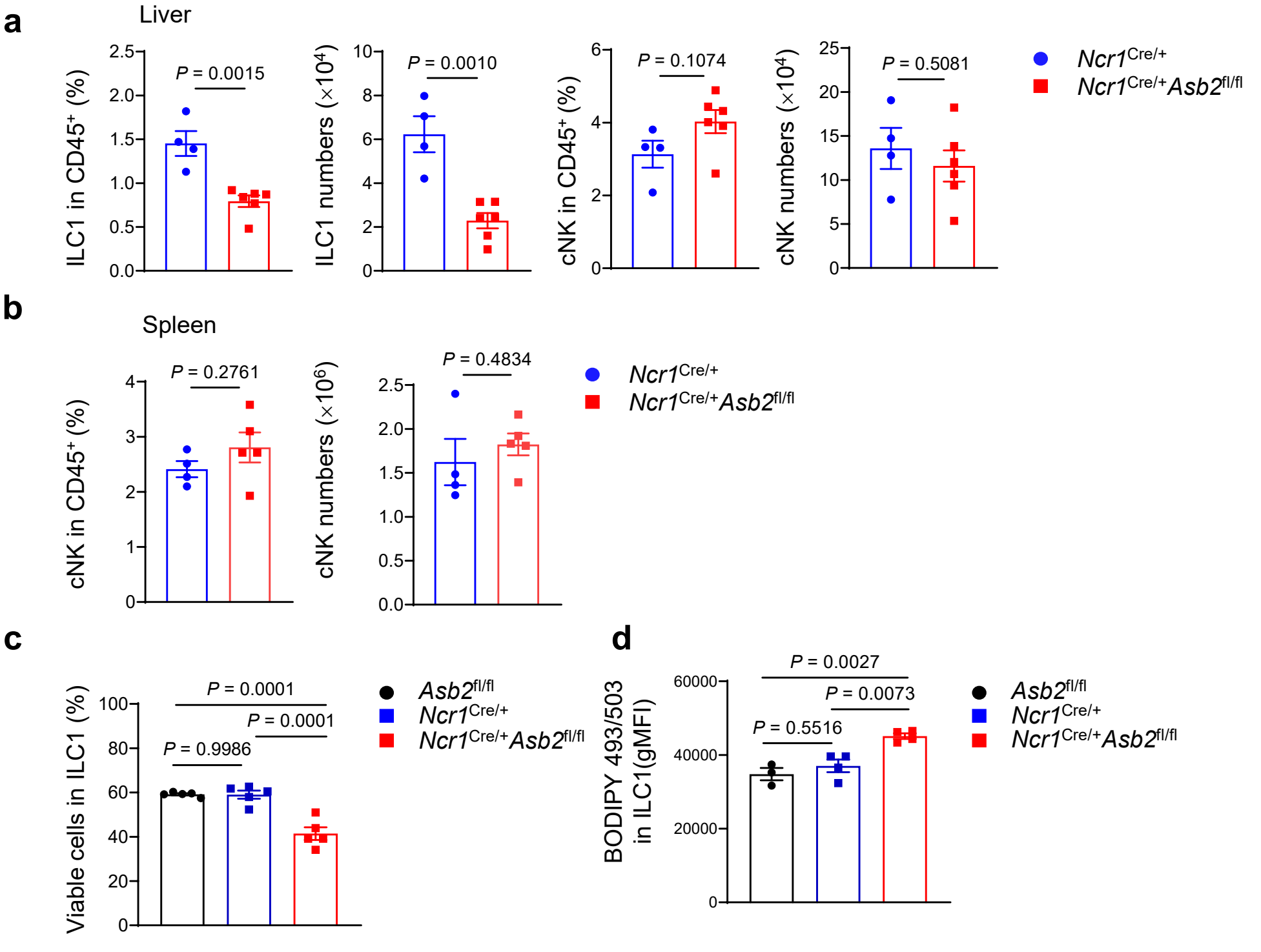

Supplementary Figure 5. ASB2 deficiency alters ILC1 activity and lipid metabolism independently of potential Cre toxicity. (a) Percentages and numbers of liver ILC1s and cNK cells from *Ncr1*$^{\text{Cre/+}}$*Asb2*$^{\text{fl/fl}}$ (n = 6; female) and *Ncr1*$^{\text{Cre/+}}$ mice (n = 4; female). **(b)** Frequencies and absolute numbers of spleen cNK cells from adult *Ncr1*$^{\text{Cre/+}}$*Asb2*$^{\text{fl/fl}}$ (n = 5; male) and *Ncr1*$^{\text{Cre/+}}$ mice (n = 4; male). **(c)** Proportions of viable (AnnexinV$^-$7-AAD$^-$) cells among liver ILC1s from adult *Ncr1*$^{\text{Cre/+}}$*Asb2*$^{\text{fl/fl}}$, *Ncr1*$^{\text{Cre/+}}$ and *Asb2*$^{\text{fl/fl}}$ mice (n = 5 per group; male). **(d)** gMFI of BODIPY-493/503 staining in ILC1s from *Ncr1*$^{\text{Cre/+}}$*Asb2*$^{\text{fl/fl}}$ (n = 4; female), *Ncr1*$^{\text{Cre/+}}$ (n = 4; female) and *Asb2*$^{\text{fl/fl}}$ mice (n = 3; female). Data are from one representative experiment of two independent experiments. Samples were compared using two-tailed unpaired Student's *t* tests **(a, b)** or one-way ANOVA **(c, d)** and were presented as means ± SEM. ILC1: type 1 innate lymphoid cell; cNK: conventional natural killer cell.

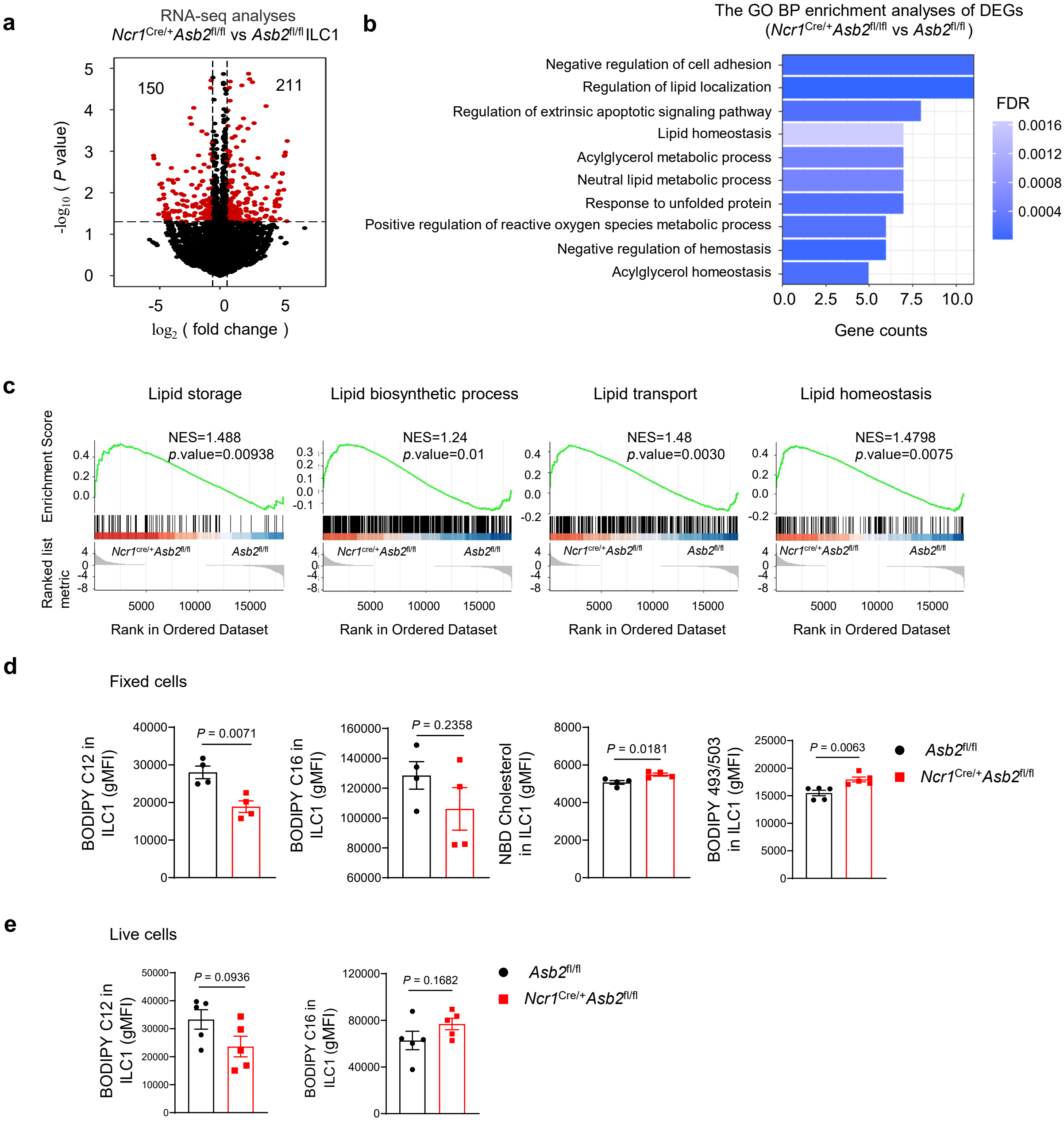

Supplementary Figure 6. ASB2-deficient ILC1s are related to fatty acid metabolism. (a–c) RNA sequencing (RNA-seq) was performed on ILC1s isolated from livers in *Ncr1*$^{Cre/+}$*Asb2*$^{fl/fl}$ or *Asb2*$^{fl/fl}$ mice (*Asb2*$^{fl/fl}$ group pooled from 29 mice of both sexes and *Ncr1*$^{Cre/+}$ *Asb2*$^{fl/fl}$ group pooled from 48 mice of both sexes). **(a)** Volcano plot showing differentially expressed genes (DEG) between ILC1s from *Ncr1*$^{Cre/+}$*Asb2*$^{fl/fl}$ and *Asb2*$^{fl/fl}$ mice. Lines indicate a 1.5-fold difference between ILC1s from *Ncr1*$^{Cre/+}$*Asb2*$^{fl/fl}$ and *Asb2*$^{fl/fl}$ mice ($p < 0.05$). **(b)** Gene ontology (GO) enrichment (biological processes) analysis showing the enriched pathways of up-regulated DEGs. **(c)** Gene-set enrichment analysis (GSEA) of *Ncr1*$^{Cre/+}$*Asb2*$^{fl/fl}$ liver ILC1s compared with the *Asb2*$^{fl/fl}$ control. **(d)** gMFI of C12 ($n = 4$ per group; male), C16 ($n = 4$ per group; male), NBD cholesterol ($n = 4$ per group; male) and BODIPY-493/503 straining ($n = 5$ per group; male) in fixed ILC1s from adult *Ncr1*$^{Cre/+}$*Asb2*$^{fl/fl}$ and *Asb2*$^{fl/fl}$ mice. **(e)** gMFI of C12 ($n = 5$ per group; female) and C16 ($n = 5$ per group; female) in liver live ILC1s from adult *Ncr1*$^{Cre/+}$*Asb2*$^{fl/fl}$ and *Asb2*$^{fl/fl}$ mice. Data are from one representative experiment of two independent experiments **(d, e).** Samples were compared using two-tailed unpaired Student's *t* tests **(d, e)** and were presented as means ± SEM. ILC1: type 1 innate lymphoid cell.

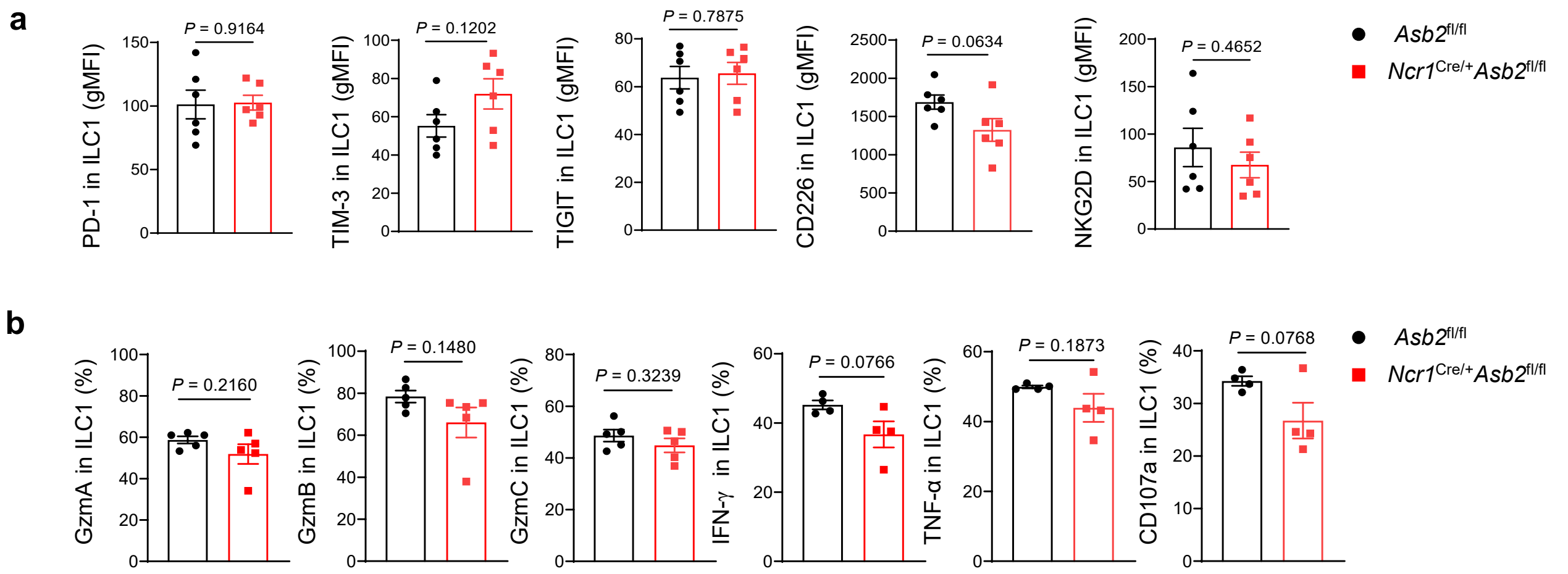

Supplementary Figure 7. ASB2 deficiency does not affect ILC1 function at steady state. (a) Expression of activating and inhibitory receptors on liver ILC1s from *Ncr1*$^{Cre/+}$*Asb2*$^{fl/fl}$ and *Asb2*$^{fl/fl}$ mice (n = 6 per group; female). **(b)** Expression of Granzyme A (GzmA) (n = 5 per group; female), Granzyme B (GzmB) (n = 5 per group; female), Granzyme C (GzmC) (n = 5 per group; male), interferon-γ (IFN-γ) (n = 4 per group; male), tumor necrosis factor alpha (TNF-α) (n = 4 per group; male) and CD107a (n = 4 per group; male) by liver ILC1s from *Ncr1*$^{Cre/+}$*Asb2*$^{fl/fl}$ and *Asb2*$^{fl/fl}$ mice. Data are from one representative experiment of two independent experiments **(b)** or are pooled from two independent experiments **(a)**. Samples were compared using two-tailed unpaired Student's t tests **(a, b)** and are presented as means ± SEM. ILC1: type 1 innate lymphoid cell.

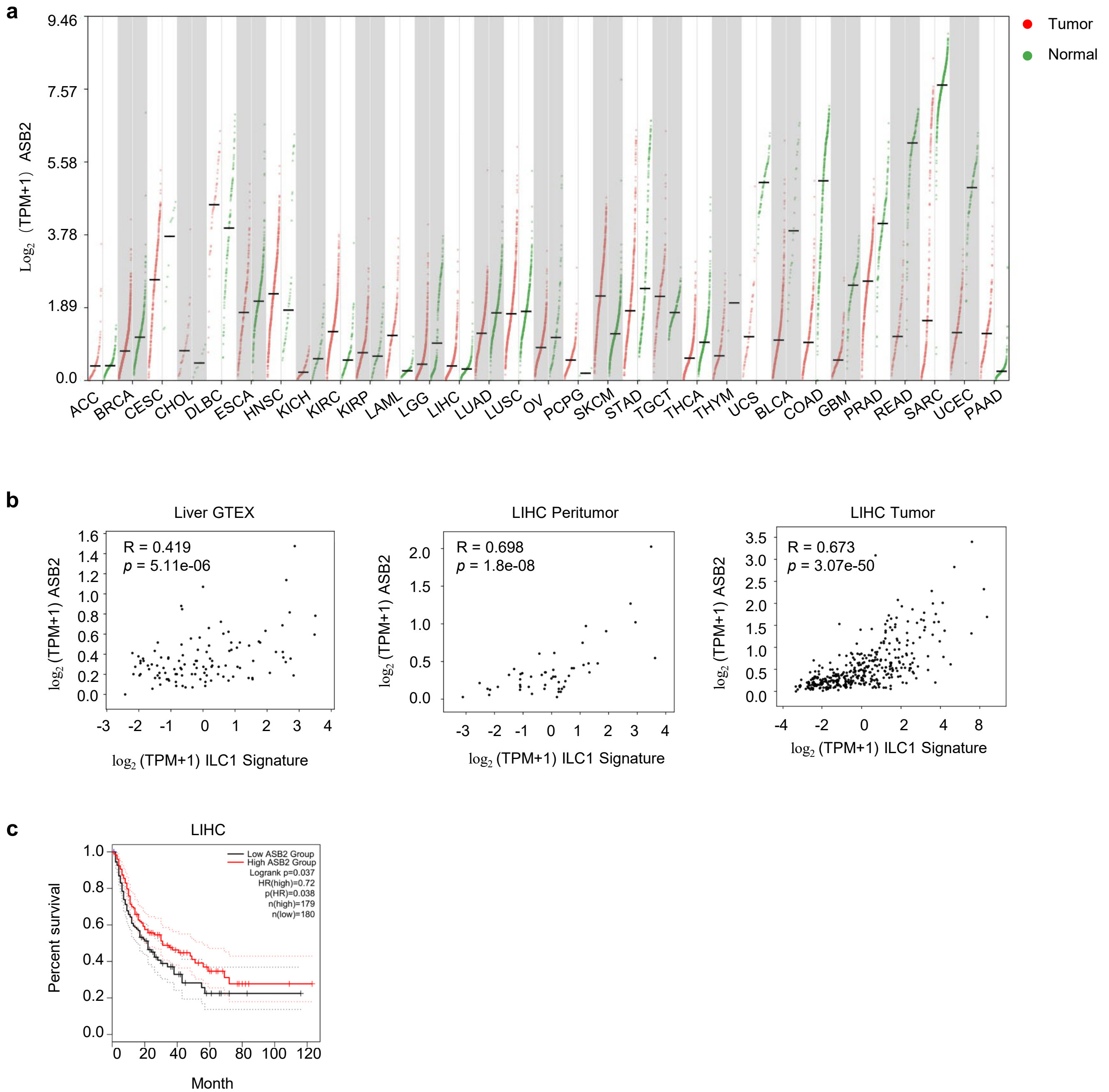

Supplementary Figure 8. Clinical relevance of *ASB2* expression in human cancers. **(a)** Expression levels of *ASB2* in normal tissues and tumor tissues using GEPIA3. **(b)** Correlation between *ASB2* expression and the expression of ILC1 signature genes (*NCAM1*, *NCR1*, *GZMK*, *RGS1*, *ZNF683*, *ITGA1*) in normal liver, LIHC peritumor, and LIHC tumor tissues using GEPIA3. **(c)** Correlation between *ASB2* expression and patient prognosis in LIHC using GEPIA2. LIHC: liver hepatocellular carcinoma.

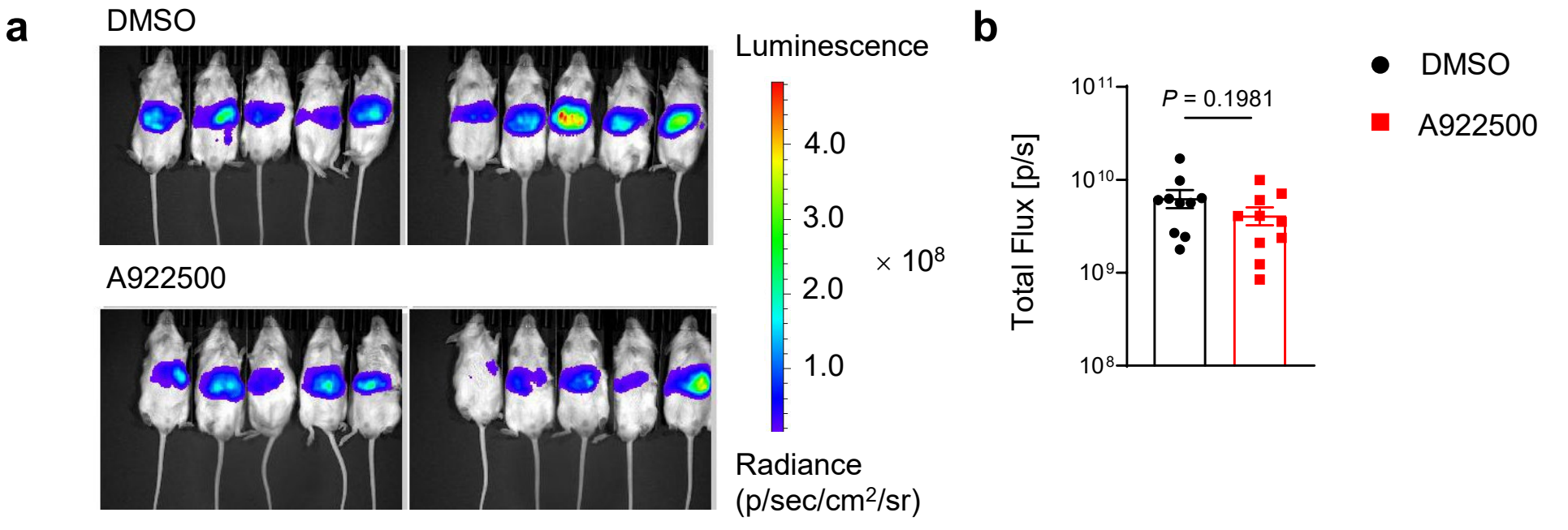

Supplementary Figure 9. 10 mg/kg A922500 has no effect on tumor cell growth directly. (a, b) NCG mice were intraperitoneally injected with DMSO or A922500 (10 mg/kg) every three days after initial intrasplenic injection of 2×10^5 MC38-luc cells. *In vivo* bioluminescence imaging of tumor metastases on day 10 (n = 10 per group; male) **(a)** and quantification of the radiance values of tumor metastases on day 10 (n = 10 per group; male) **(b)** . Data are from one representative experiment .Samples were compared using two-tailed unpaired Student's t tests and were presented as means ± SEM.

Supplementary Table 1. List of antibodies and fluorochromes

Conjugate	Target	Source	Clone	Cat. number	Dilution (1×10^6 cells)
eFluor 660	Ki67	eBioscience	SolA15	50-5698-82	1:200
eFluor 660	GATA3	eBioscience	TWAJ	50-9966-42	1:50
APC	mMR1 tetramer	National Institues of health	6-FP	65845	1:2000
APC	CD4	Biolegend	RM4-4	116014	1:200
APC	PD-1	Biolegend	29F.1A12	135210	1:200
APC	Flt3	Biolegend	A2F10	135310	1:200
APC	CD226	Biolegend	10E5	128810	1:200
APC	Granzyme A	eBioscience	GzA-3G8.5	17-5831-82	1:200
Alexa 647	NKp46	Biolegend	29A1.4	137628	1:200
Alexa-488	CD90.2	Biolegend	30-H12	105316	1:200
FITC	CD107a	BD	1D4B	553793	1:100
FITC	Annexin V	BD		556419	1:50
FITC	CD19	BD	1D3	557398	1:200
FITC	CD3	Biolegend	145-2C11	100306	1:200
FITC	Caspase 3	BD	C92-605	560901	1:50
BV421	RORγt	BD	Q31-378	562894	1:200
BV421	CD49b	BD	DX5	563063	1:200
BV421	TNF-α	Biolegend	MP6-XT22	506328	1:200
BV421	TIGIT	Biolegend	1G9	142111	1:200
BV421	Tim3 (CD336)	Biolegend	B8.2C12	134019	1:200
BV421	CD127(IL-7Rα)	Biolegend	A7R34	135024	1:200
PE	Granzyme B	Biolegend	QA16A02	372208	1:200
PE	ST2	Biolegend	DIH4	146608	1:200
PE	Granzyme C	Biolegend	SFC1D8	150804	1:200
PE	CD8α	BD	53-6.7	553033	1:200
PE	NKG2D (CD314)	BD	CX5	558403	1:200
PE	CD127 (IL-7Ra)	Biolegend	A7R34	135010	1:200
Percp-CY5.5	IFN-γ	Biolegend	XMG1.2	505822	1:200
Percp-CY5.5	CD4	Biolegend	GK1.5	100434	1:200
	7AAD	BD		559925	1:100
Percp-CY5.5	CD19	Biolegend	6D5	115534	1:200
Percp-CY5.5	Gr1	Biolegend	RB6-8C5	108428	1:200
Percp-CY5.5	CD3e	Biolegend	145-2C11	100328	1:200
Percp-CY5.5	CD11b	Biolegend	M1/70	101228	1:200
Percp-CY5.5	NK1.1	Biolegend	PK136	108728	1:200
Percp	Ter119	Biolegend	TER-119	116226	1:200
Percp-eFluor 710	Rorγt	Invotrogen	B2D	46-6981-82	1:200
Percp-CY5.5	CD11c	Biolegend	N418	117326	1:200
Percp-CY5.5	LAG3	BD	C9B7W	564673	1:200
BV786	CD49a	BD	Ha31/8	740919	1:200
BV786	IFN-γ	BD	XMG1.2	563773	1:200
BV785	TCR β chain	Biolegend	H57-597	109249	1:200
BV786	α4β7	BD	DATK32	740892	1:200
BV786	CD19	BD	1D3	563333	1:200
BV786	CD3	BD	145-2C11	564379	1:200
BUV395	TCR β chain	BD	H57-597	742485	1:200
BUV395	CD49a	BD	Ha31/8	740262	1:200
BUV395	CD3	BD	145-2C11	563565	1:200
BV605	NK1.1	Biolegend	PK136	108740	1:200
BV605	CD25	Biolegend	PC61	102036	1:200
APC-CY7	CD45.2	Biolegend	104	109824	1:200
BV510	Fixable Viability Stain	BD		564406	1:1000
BV510	CD4	BD	RM4-5	563106	1:200
BUV737	CD49b	BD	HMα2	741752	1:200
BUV737	CD8α	BD	53-6.7	612759	1:200
PE-CY7	CD49b	Biolegend	DX5	108922	1:200
PE-CY7	NKp46	Biolegend	29A1.4	137618	1:200
PE-CY7	NK1.1	Biolegend	PK136	108714	1:200

Supplementary Table 2. RT-qPCR primer sequences

Mouse *β-actin* forward	5'-TGACGTTGACATCCGTAAAGACC-3'
Mouse *β-actin* reverse	5'-CTCAGGAGGAGCAATGATCTTGA-3'
Mouse *Asb2α* forward	5'-TTCACTCTGGCTCTGCACCTTC-3'
Mouse *Asb2α* reverse	5'-TCTTCAAGGCCTCTTCATCCC-3'
Mouse *Asb2β* forward	5'- CTTTGTTGCCCAGACCTGTG -3'
Mouse *Asb2β* reverse:	5'- CTCGCTCAGGCTGCTGTATAG -3'
Mouse *Asb2* forword	5'-TGTTCCAAGGGGTCATGCAG-3'
Mouse *Asb2* reverse	5'-CTTCAGCACGGGGTCCATAG -3'

Supplementary Table 3. List of Western antibodies

Antibodies	Source	Cat. Number	Dilution
GAPDH Monoclonal Antibody	Proteintech	60004-1-Ig	1:5000
HRP-conjugated Goat Anti-Mouse IgG(H+L)	Proteintech	SA00001-1	1:5000
HRP-linked-Anti-rabbit IgG	Cell signaling technology	7074S	1:1000
MYC mouse tag Monoclonal antibody	Proteintech	60003-2-Ig	1:5000
Rabbit MYC tag Polyclonal antibody	Proteintech	16286-1-AP	1:5000
Mouse monoclonal Anti-FLag M2 antibody	Sigma	F1804	1:1000
Anti-Flag Tag Rabbit Polyclonal antibody	Servicebio	GB11938	1:1000
β-actin	Proteintech	60008-1-Ig	1:5000
