# Supplementary material for: ASB2 inhibits lipid accumulation to promote ILC1 homeostatic fitness and anti-tumor immunity in the mouse liver
Source: Nat Commun. 2026 Jul 17;17:8741. doi: 10.1038/s41467-026-75517-4 (PMC13493952; doi:10.1038/s41467-026-75517-4)
Supplement: Supplementary file 2 [file 41467_2026_75517_MOESM2_ESM.pdf]

Description of Supplementary files

Supplementary data 1: The differentially expressed genes in liver ILC1s from *Asb2*$^{fl/fl}$ and *Ncr1*$^{Cre/+}$*Asb2*$^{fl/fl}$ mice.

Supplementary data 2: Enriched pathways of differentially expressed genes in liver ILC1s from *Ncr1*$^{Cre/+}$ *Asb2*$^{fl/fl}$ and *Asb2*$^{fl/fl}$ mice.

Supplementary data 3: The differentially expressed proteins in liver ILC1s from *Asb2*$^{fl/fl}$ and *Ncr1*$^{Cre/+}$*Asb2*$^{fl/fl}$ mice.

Ncr1$^{Cre/+}$*Asb2*$^{fl/fl}$: CKO ILC1_1、CKO ILC1_2

Asb2$^{fl/fl}$: WT ILC1_1、WT ILC1_2、WT ILC1_3

Supplementary data 4: Enriched pathways of differentially expressed proteins in liver ILC1s from *Ncr1*$^{Cre/+}$ *Asb2*$^{fl/fl}$ and *Asb2*$^{fl/fl}$ mice.
