# Supplementary material for: ASB2 inhibits lipid accumulation to promote ILC1 homeostatic fitness and anti-tumor immunity in the mouse liver
Source: Nat Commun. 2026 Jul 17;17:8741. doi: 10.1038/s41467-026-75517-4 (PMC13493952; doi:10.1038/s41467-026-75517-4)
Supplement: Supplementary file 3 [file 41467_2026_75517_MOESM7_ESM.pdf]

nature portfolio

Corresponding author(s): Zhigang Tian
Hui Peng
Xianwei Wang

Last updated by author(s): 29/6/26

Reporting Summary

Nature Portfolio wishes to improve the reproducibility of the work that we publish. This form provides structure for consistency and transparency in reporting. For further information on Nature Portfolio policies, see our Editorial Policies and the Editorial Policy Checklist.

Statistics

For all statistical analyses, confirm that the following items are present in the figure legend, table legend, main text, or Methods section.

n/a	Confirmed	
☐	☒	The exact sample size (*n*) for each experimental group/condition, given as a discrete number and unit of measurement
☐	☒	A statement on whether measurements were taken from distinct samples or whether the same sample was measured repeatedly
☐	☒	The statistical test(s) used AND whether they are one- or two-sided<br>*Only common tests should be described solely by name; describe more complex techniques in the Methods section.*
☒	☐	A description of all covariates tested
☒	☐	A description of any assumptions or corrections, such as tests of normality and adjustment for multiple comparisons
☐	☒	A full description of the statistical parameters including central tendency (e.g. means) or other basic estimates (e.g. regression coefficient) AND variation (e.g. standard deviation) or associated estimates of uncertainty (e.g. confidence intervals)
☐	☒	For null hypothesis testing, the test statistic (e.g. *F*, *t*, *r*) with confidence intervals, effect sizes, degrees of freedom and *P* value noted<br>*Give P values as exact values whenever suitable.*
☒	☐	For Bayesian analysis, information on the choice of priors and Markov chain Monte Carlo settings
☒	☐	For hierarchical and complex designs, identification of the appropriate level for tests and full reporting of outcomes
☒	☐	Estimates of effect sizes (e.g. Cohen's *d*, Pearson's *r*), indicating how they were calculated

Our web collection on statistics for biologists contains articles on many of the points above.

Software and code

Policy information about availability of computer code

Data collection	Flow cytometry data were collected on X-20 and LSRFortessa; (BD Bioscience);Sorted cells were captured using BD FACSAria Fusion (BD Bioscience).RNA-seq were performed by Wuhan Seqhealth in China. Proteomes were performed by iProteome in China
Data analysis	FlowoJ (V10) was used to analyse flow cytometry data. R (version 4.3.1)(Deseq2, limma,Seurat) was used to analyse RNA-Seq data and Microarray data. Graphpad prism 9 (Graphpad) was used for analysis of phenotype assays and for graph production.For more details on these Methods and specific references please see Methods section

For manuscripts utilizing custom algorithms or software that are central to the research but not yet described in published literature, software must be made available to editors and reviewers. We strongly encourage code deposition in a community repository (e.g. GitHub). See the Nature Portfolio guidelines for submitting code & software for further information.

Data

Policy information about availability of data

All manuscripts must include a data availability statement. This statement should provide the following information, where applicable:

- Accession codes, unique identifiers, or web links for publicly available datasets
- A description of any restrictions on data availability
- For clinical datasets or third party data, please ensure that the statement adheres to our policy

The RNA-seq data generated in this study have been deposited in the China National Center for Bioinformation (CNCB) under accession code CRA030972 (https://

ngdc.cncb.ac.cn/gsa/browse/CRA030972); The Proteome data generated in this study have been deposited in the ProteomeXchange Consortium via the iProX partner repository under accession code PXD069231. (https://www.iprox.cn//page/project.html?id=IPX0013726000). scRNA-seq and microarray data from publicly accessible datasets were retrieved from GSE202475(https://www.ncbi.nlm.nih.gov/geo/query/acc.cgi?acc=GSE202475) and GSE37448(https://ncbi.nlm.nih.gov/geo/query/acc.cgi?acc=GSE37448). Supplementary Data (Supplementary Data 1-4) are provided in the file titled "Description of Supplementary files". All data are included in the Supplementary Information or available from the authors, as are unique reagents used in this Article. The raw numbers for charts and graphs are available in the Source Data file whenever possible.

Research involving human participants, their data, or biological material

Policy information about studies with human participants or human data. See also policy information about sex, gender (identity/presentation), and sexual orientation and race, ethnicity and racism.

Reporting on sex and gender	N/A
Reporting on race, ethnicity, or other socially relevant groupings	N/A
Population characteristics	N/A
Recruitment	N/A
Ethics oversight	N/A

Note that full information on the approval of the study protocol must also be provided in the manuscript.

Field-specific reporting

Please select the one below that is the best fit for your research. If you are not sure, read the appropriate sections before making your selection.

☒ Life sciences ☐ Behavioural & social sciences ☐ Ecological, evolutionary & environmental sciences

For a reference copy of the document with all sections, see nature.com/documents/nr-reporting-summary-flat.pdf

Life sciences study design

All studies must disclose on these points even when the disclosure is negative.

Sample size	The animal numbers were determined based on previous studies demonstrating statistical significance in similar experimental settings. Although no formal power analysis was performed, the selected sample sizes were empirically validated as sufficient to achieve statistical significance in our established tumor models
Data exclusions	None
Replication	Replicates were used in all experiments as noted in figure legends.
Randomization	Mice were matched by age, sex, and genetic background and randomized appropriately. For A922500-treated tumor-bearing mice, size-matched C57BL/6 mice and NCG mice were randomly assigned to experimental groups before each experiment began.
Blinding	Blinding was not performed due to the necessity for cage labeling and genotypic differences among the mice.

Reporting for specific materials, systems and methods

We require information from authors about some types of materials, experimental systems and methods used in many studies. Here, indicate whether each material, system or method listed is relevant to your study. If you are not sure if a list item applies to your research, read the appropriate section before selecting a response.

Materials & experimental systems

n/a	Involved in the study
☐	☒ Antibodies
☐	☒ Eukaryotic cell lines
☒	☐ Palaeontology and archaeology
☐	☒ Animals and other organisms
☒	☐ Clinical data
☒	☐ Dual use research of concern
☒	☐ Plants

Methods

n/a	Involved in the study
☒	☐ ChIP-seq
☐	☒ Flow cytometry
☒	☐ MRI-based neuroimaging

Antibodies

Antibodies used

mouse CD90.2 (30-H12) Alexa-488 Biolegend 105316,1:200
mouse RORγt (Q31-378) BV421 BD 562894,1:200
mouse CD107a (1D4B) FITC BD 553793,1:100
mouse CD49b (DX5) BV421 BD 563063,1:200
mouse CD49a (Ha31/8) BV786 BD 740919,01:200
mouse Granzyme B (QA16A02) PE Biolegend 372208,1:200
mouse IFN-γ (XMG1.2) BV786 BD 563773,1:200
mouse NK1.1 (PK136) BV605 Biolegend 108740,1:200
mouse Ki67 (SolA15) eFluor 660 eBioscience 50-5698-82,1:200
mouse CD45.2 (104) APC-CY7 Biolegend 109824,1:200
mouse CD3 (145-2C11) BUV395 BD 563565,1:200
mouse CD19 (6D5) Percp-CY5.5 Biolegend 115534,1:200
mouse CD4 (RM4-4) APC Biolegend 116014,1:200
mouse PD-1 (29F.1A12) APC Biolegend 135210,1:200
mouse TNF-α (MP6-XT22) BV421 Biolegend 506328,1:200
mouse ST2 (DIH4) PE Biolegend 146608,1:200
mouse CD4 (RM4-5) BV510 BD 563106,1:200
mouse CD49b (HMα2) BUV737 BD 741752,1:200
mouse Granzyme C (SFC1D8) PE Biolegend 150804,1:200
mouse IFN-γ (XMG1.2) Percp-CY5.5 Biolegend 505822,1:200
mouse CD8α (53-6.7) PE BD 553033,1:200
mouse TIGIT (1G9) BV421 Biolegend 142111,1:200
mouse NKG2D (CD314) (CX5) PE BD 558403,1:200
mouse CD226 (10E5) APC Biolegend 128810,1:200
mouse Tim3 (CD336) (B8.2C12) BV421 Biolegend 134019,1:200
mouse Granzyme A (GzA-3G8.5) APC eBioscience 17-5831-82,1:200
mouse LAG3 (C9B7W) Percp-CY5.5 BD 564673,1:200
mouse TCR β chain (H57-597) BV785 Biolegend 109249,1:200
mouse Annexin V - FITC BD 556419,1:50
mouse 7AAD - BD 559925,1:100
mouse CD127 (A7R34) PE Biolegend 135010,1:200
mouse CD127(IA7R34) BV421 Biolegend 135024,1:200
mouse NK1.1 (PK136) PE-CY7 Biolegend 108714,1:200
mouse CD19 (1D3) FITC BD 557398 ,1:200
mouse CD3 (145-2C11) FITC Biolegend 100306,1:200
mouse GATA3 (TWAJ) eFluor 660 eBioscience 50-9966-42,1:50
mouse Caspase 3 (C92-605) FITC BD 560901,1:50
mouse CD3 (145-2C11) BV786 BD 564379,1:200
mouse TCRβ(H57-597), BUV395 BD 742485,1:200
mouse CD8α(53-6.7), PE BD 553033,1:200
mouse CD25(PC61),BV605, Biolegend, 102036,1:200
mouse CD49a(Ha31/8), BUV395,BD,740262,1:200
mouse Gr1(RB6-8C5), Percp-CY5.5,Biolegend,108428,1:200
mouse CD3e(145-2C11),Percp-CY5.5,Biolegend,100328,1:200
mouse CD11b(M1/70),Percp-CY5.5,Biolegend,101228,1:200
mouse NK1.1(PK136),Percp-CY5.5,Biolegend,108728,1:200
mouse Ter119(TER-119),Percp,Biolegend,116226,1:200
mouse CD19(1D3),BV786,BD,563333,1:200
mouse NKp46(29A1.4),Alexa 647,Biolegend,137628,1:200
mouse CD11c(N418),Percp-CY5.5,Biolegend,117326,1:200
mouse Rorγt(B2D),Percp-eFluor 710,Invotrogen,46-6981-82,1:200
mouse Flt3(A2F10),APC,Biolegend,135310,1:200
mouse α4β7(DATK32),BV786,BD,740892,1:200
mouse NKp46 (29A1.4), PE-CY7, Biolegend,137618,1:200
mouse CD8α(53-6.7),BUV737,BD, 612759,1:200
mouse CD4(GK1.5), Percp-CY5.5,Biolegend,100434,1:200
mouse CD49b(DX5),PE-CY7,Biolegend,108922,1:200
Fixable Viability Stain, BV510, BD,564406,1:1000

	mouse MR1 terramer(6-FP), APC, National Institues of health, 65845,1:2000<br>β-actin (Proteintech, 60008-1-Ig; 1:5,000)<br>MYC tag Monoclonal antibody (Proteintech, 60003-2-Ig; 1:5000)<br>Rabbit MYC tag Polyclonal antibody (Proteintech, 16286-1-AP; 1:5000)<br>HRP-conjugated Affinipure Goat Anti-Mouse IgG(H+L) (Proteintech, SA00001-1; 1:5000)<br>GAPDH Monoclonal antibody (Proteintech, 60004-1-Ig; 1:5000)<br>Anti-rabbit IgG, HRP-linked Antibody (Cell signaling technology, 7074S;1:1000)<br>Mouse monoclonal Anti-FLag M2 antibody (Sigma, F1804, 1:1000)<br>Anti-Flag Tag Rabbit Polyclonal antibody (Servicebio, GB11938, 1:1000)
Validation	All antibodies were well-recognized clones in the field and validated by the manufactures. These antibodies are further validated and routinely used in our lab.

Eukaryotic cell lines

Policy information about cell lines and Sex and Gender in Research

Cell line source(s)	The MC38 cell line was kindly provided by Prof Yang-xin Fu (Tsing Hua University, Beijing, China), and the HEK-293T cell line was obtained from the Cell Bank of the Type Culture Collection of the Chinese Academy of Sciences (Shanghai, China).
Authentication	All cell lines were authenticated based on their growth characteristics and morphological features.
Mycoplasma contamination	All cell lines were tested mycoplasma negative.
Commonly misidentified lines (See ICLAC register)	None

Animals and other research organisms

Policy information about studies involving animals; ARRIVE guidelines recommended for reporting animal research, and Sex and Gender in Research

Laboratory animals	C57BL/6 mice (Cat. NO. N000013) and NCG mice (Cat. NO. T001475) were purchased from GemPharmatech (Nanjing, China). Ncr1Cre/+ mice were generously provided by Eric Vivier (Aix Marseille Université, Marseille, France). VavCre/+ mice were generously provided by Zhongjun Dong (Tsing Hua University, Beijing, China). Ncr1CreERT2/+ mice (Cat. NO. NM-KI-200004) were obtained from Shanghai Model Organisms Center (Shanghai, China). Asb2fl/fl mice (Cat. NO. S-CKO-12815) were purchased from Cyagen (Suzhou, China). The Asb2 gene is located on mouse chromosome 12. Ten exons are identified, with the ATG start codon in exon 2 and the TAA stop codon in exon 10. Exon 4 is selected as conditional knockout region. The region contains 167 bp coding sequence. Deletion of this region result in the loss of function of the mouse Asb2 gene. Asb2EGFP mice were generated by the Laboratory Animal Center of University of Science and Technology of China (USTC). Asb2α and Asb2β share no common N-terminus but have a conserved C-terminus and the EGFP knock-in strategy was designed to target the C-terminus of Asb2, immediately upstream of the stop codon TAA. For proteomic and RNA-seq profiling, ILC1s were purified from a pool of multiple adult mice including both females and males. For other experiments of adult mice, age- (6–14 weeks old) and sex-matched mice were used within each individual experiment to minimize biological variation, and independent replicate experiments were performed using either male or female mice independently. Sex was not distinguished for E17.5 embryonic mice and neonatal mice. This study primarily focuses on regulatory mechanism of ASB2 in mouse liver ILC1s. To date, no published studies have reported sex bias of liver ILC1s; therefore, we did not conduct sex-based comparative analyses. All mice were housed in a specific-pathogen-free (SPF) facility, adhering to the experimental animal care standards set by the University of Science and Technology of China. All mice were housed in animal facilities on a 12 hour dark/light cycle with ambient temperature of 20-26? and relative humidity of 40-70%. Asb2fl/fl mice and Ncr1Cre/+Asb2fl/fl mice or VavCre/+Asb2fl/fl were co-housed. Ncr1CreERT2/+ and Asb2fl/flNcr1CreERT2/+ mice were bred separately. Ncr1Cre/+ and Ncr1Cre/+Asb2fl/fl mice were bred separately. All of the mouse experiments were approved by the Ethics Committee of University of Science and Technology of China and the protocol number is USTCACUC26070122077.
Wild animals	No wild animals were used in this study
Reporting on sex	We found no sex-related differences. Therefore, both male and female mice were used in all experiment including tumor model, as described in the Methods section.
Field-collected samples	The study did not involve samples collected from field.
Ethics oversight	All of the mouse experiments were approved by The Ethics Committee of University of Science and Technology of China.

Note that full information on the approval of the study protocol must also be provided in the manuscript.

Plants

Seed stocks: N/A

Novel plant genotypes: N/A

Authentication: N/A

Flow Cytometry

Plots

Confirm that:

☒ The axis labels state the marker and fluorochrome used (e.g. CD4-FITC).

☒ The axis scales are clearly visible. Include numbers along axes only for bottom left plot of group (a 'group' is an analysis of identical markers).

☒ All plots are contour plots with outliers or pseudocolor plots.

☒ A numerical value for number of cells or percentage (with statistics) is provided.

Methodology

Sample preparation: Liver tissues were homogenized by passing through a 200-gauge mesh, and liver MNCs were purified via Percoll (Cytiva) density gradient centrifugation (40% and 70%). Splenocytes were isolated by pressing excised spleens through a 200-gauge mesh, followed by red blood cell lysis with red cell lysis buffer (Solarbio). Lung tissues were minced into small fragments and enzymatically digested in RPMI 1640 medium (VivaCell) containing 0.1% collagenase I (Sigma) at 37°C for 40 min with constant agitation. The digested suspension was then centrifuged over a Percoll gradient for cell purification. The mesentery of the small intestine was carefully excised and gently rinsed to remove luminal contents. The intestines were cut into pieces and were incubated in 20 ml Extraction buffer supplemented with 5% FBS (Gibco), 5 mM EDTA, and 15 mM Hepes and rotated at 200 rpm for 30 min at 37°C for twice. The intestine was washed, minced, and digested with DMEM (VivaCell) containing 10% FBS, 0.1% collagenase IV (Sigma) and 0.25 mg/ml DNase I (Sigma) with shaking at 37°C for 1h. The supernatants were filtered through a 200-gauge steel mesh, and IELs were collected after Percoll gradient centrifugation.

Instrument: Data collection and analysis was performed on LSRFortessa(BD) ,X-20(BD), FACSAria Fusion (BD).

Software: All data were analyzed with FACS FlowJo v10.8.1.

Cell population abundance: To obtain ILC1s or RNA-seq and Proteome , cell populations were sorted to >90% purity, as determined by flow cytometry.

Gating strategy:

small intestine ILC1:CD45.2+FVS-NK1.1+CD19-CD3- NKp46+ CD127+
small intestine cNK :CD45.2+FVS-NK1.1+CD19-CD3-NKp46+CD127-
small intestineILC2 :CD45.2+FVS-NK1.1-CD19-CD3-CD127+ GATA3+
small intestineILC3 :CD45.2+FVS-NK1.1-CD19-CD3-CD127+RORγt+
lung ILC2: CD45.2+FVS-NK1.1-CD19-CD3-CD127+ ST2+GATA3+
lung/liver/spleen cNK:CD45.2+FVS-NK1.1+CD3-NKp46+CD49b+CD49a-
liver NKT cells :CD45.2+FVS-NK1.1+TCRβ+
liver/spleen MAIT cells :CD45.2+FVS-NK1.1-TCRβ+MR1 tetramer+
liver/spleen B cells :CD45.2+FVS-NK1.1-TCRβ-CD19+
liver/spleen CD4+ T cells :CD45.2+FVS-NK1.1-TCRβ+CD4+CD8-
liver/spleen CD8 + T cells :CD45.2+FVS-NK1.1-TCRβ+CD4-CD8+
liver ILC1:CD45.2+FVS-NK1.1+CD3-NKp46+CD49b-CD49a+
bone ILCP: CD45.2+Lin-(Lin- : NK1.1-CD11b-CD11c-CD19-CD3- Gr1- Ter119-)CD127+CD25-Flt3-α4β7+ PD-1+

☒ Tick this box to confirm that a figure exemplifying the gating strategy is provided in the Supplementary Information.
