# Supplementary material for: ASB2 inhibits lipid accumulation to promote ILC1 homeostatic fitness and anti-tumor immunity in the mouse liver
Source: Nat Commun. 2026 Jul 17;17:8741. doi: 10.1038/s41467-026-75517-4 (PMC13493952; doi:10.1038/s41467-026-75517-4)
Supplement: Supplementary file 4 [file 41467_2026_75517_MOESM8_ESM.pdf]

nature portfolio

Peer Review File

ASB2 inhibits lipid accumulation to promote ILC1 homeostatic fitness and anti-tumor immunity in the mouse liver

Corresponding Author: Professor Zhigang Tian

This file contains all reviewer reports in order by version, followed by all author rebuttals in order by version.

Version 0:

Reviewer comments:

Reviewer #1

(Remarks to the Author)
In this manuscript, Bao et al. reported that ASB2 is specifically expressed in liver ILC1s and knockout of ASB2 in ILCs and NK cells reduces ILC1 cell population in the liver. Mechanistically, they showed that loss of Asb1 induces lipid accumulation, resulting in cell death. They demonstrated that DGAT1 inhibitor significantly reduces lipid accumulation and rescues cell death in Asb1 knockout ILCs. Finally, they showed that Asb1 knockout promotes colorectal cancer metastasis to liver and DGAT1 inhibitor reduces cancer growth. While the authors presented solid evidence that ASB2 is important for ILC1 survival, the mechanistic studies are not convincing. There are major gaps in their claimed mechanisms and the data presented do not fully support the conclusions.
1. The authors claimed that there is increased lipid accumulation in the cells in the absence of Asb2. This conclusion is only based on BODIPY staining, which is not sufficient. They showed directly measure lipid levels in the cells. They claimed that lipid accumulation induces cell damage and apoptosis. It is known that free fatty acids and cholesterol can induce toxicity and cell death. What kind of lipids are increased in Asb2 knockout cells?
2. The authors showed Asb2 knockout increases FABP1 and DGAT1 expression, which they claimed to be responsible for lipid accumulation. They did not show direct evidence that Asb2 knockout indeed increase fatty acid uptake and lipid biosynthesis.
3. The authors need to provide direct evidence that DGAT1 inhibitor treatment indeed reduces cellular lipid levels.
4. In Fig. 6, the authors claimed that DGAT1 inhibitor treatment reduces tumor growth through modulating ILC1s. Does DGAT1 inhibitor itself have any effect on tumor growth? They need to test if tumor growth is also inhibited by DGAT1 inhibitor in the tumor cells without endogenous DGAT1.
Minor points:
1. Line 132-133, Fig. 4a and 4b showed PA treated cells, not SA. Fig. 4c and 4d showed SA treated cells, not PA.
2. Typo: Line 41, dirven should be driven.

Reviewer #2

(Remarks to the Author)
This manuscript identifies a role for ABS2 in limiting lipid accumulation that induces apoptosis of adult liver ILC1s, thereby restraining the progression of liver metastasis. The study provides a novel mechanistic link between lipid metabolism and the maintenance of adult liver ILC1s. While the work is original and potentially impactful, additional experimental evidence and greater methodological detail are needed to strengthen the conclusions and ensure reproducibility. In addition, it generates a new tool that will be of value to the field. With revision, the study could make a valuable contribution to our understanding of the metabolic regulation of liver-resident ILCs.
The authors demonstrate that ASB2 reduces lipid accumulation at steady-state and in tumor microenvironment, thereby protecting ILC1s from lipid-induced apoptosis. Loss of ASB2 compromises ILC1 survival and correlates with enhanced metastatic progression. Integration of sequencing data with in vivo mouse experiments supports the proposed mechanism that ASB2 maintains lipid metabolic balance in the adult liver. The work broadens our understanding of how lipid metabolism influences the maintenance and function of adult liver ILC1s, an area that remains relatively underexplored.

The authors have focused only on mature ILC1 in the liver. They have generated a very nice reporter mouse but have provided limited characterisation of this new strain. Could the authors provide a more thorough characterisation of this strain across multiple cell types (particularly other ILCs, NK T cells, MAIT cells and adaptive immune cells) and multiple tissues (e.g., gut, lung, spleen) to assess tissue specificity (as supplementary data). Analysis of the expression of the reporter relative to gene expression is also required.

What is the expression of ASB2 in early bone marrow progenitors? and is expression required for development of ILC?

Amongst mature cells, little functional data is provided. The authors should provide data on the functional effector changes (e.g., granzyme B and IFN-γ production), if any, induced by loss of ASB2 in addition their assessment of cell viability.

What is the expression of ASB2 in human tissues and tumours and potential correlation with ILC1 frequency and prognosis in human cancers or liver metastases, for example using TCGA datasets.

The authors should provide more detailed information on in vitro cell culture conditions, and lipid quantification protocols. Additional methodological description is required for analysis of the liver ILC RNA sequencing. These data do not appear to have been provided in a publicly accessible database. This is important that this is available on publication of the manuscript.

The expansion of the $CD49a^{+}CD49b^{+}$ population within tumours has been reported in multiple studies. Have the authors observed a similar expansion in their tumour model? What are the lipid levels within this population, and are they affected by Asb2?

In addition to the tumour tissue, did the authors examine the adjacent liver tissue? If so, what are the ILC1 numbers and lipid levels in the adjacent liver compared with the tumour site?

Overall, this is a solid manuscript which makes a novel contribution to the field and would be strengthened by substantial revisions and clarification.

Reviewer #3

(Remarks to the Author)
I co-reviewed this manuscript with one of the reviewers who provided the listed reports. This is part of the Nature Communications initiative to facilitate training in peer review and to provide appropriate recognition for Early Career Researchers who co-review manuscripts.

Reviewer #4

(Remarks to the Author)
Through the analyses of publicly available single cell and bulk RNA-seq data, and the use of an Asb2-EGFP mouse, the authors showed that ASB2 is expressed in mouse adult liver ILC1s. Transcriptomic and proteomic analyses reveal a profound alteration of gene and protein expression following ASB2 deletion in liver ILC1s, associated with impaired cell survival. They further propose that deletion of ASB2 increase lipid storage in liver ILC1, leading to reduced ILC1 survival and increased tumor burden in the liver after intrasplenic injection of MC38 cells.

There are many issues that need to be fixed to enable authors to fully support the conclusions made in the manuscript.

Major issues:

1. Although interesting, the finding that ASB2 is expressed in ILC1 is not new (McFarland et al, Immunity 2021; Frey et al, iScience 2025), but this is not mentioned in the manuscript. In addition, the authors reported specific expression of ASB2 in adult liver ILC1s compared to in other tissue-resident ILC1s, including small intestine. These results contrast with those mentioned above (McFarland et al, Immunity 2021) that showed that ILC1s in the small intestine selectively expressed ASB2 relative to the liver, visceral adipose tissue, salivary glands and uterus.
Since the comparative analysis of ASB2 expression in different populations / tissues was performed using 3 different datasets and thus requires data normalization (Fig1h), complementary approaches should be used to analyze the expression of ASB2 in ILC1s of different mouse tissues.
The authors used “Asb2-EGFP mice” but there is no description of these mice throughout the manuscript. A clear description of the construct is absolutely required to evaluate the data obtained with these mice.

2. The ASB2 gene encodes two transcripts that are translated into two protein isoforms that differ in their N-terminus, Isoform 1/ASB2β (Bello et al, Cell Death & Diff 2009; Davey et al, JCI Insight 2016; Jang et al, J Cachexia Sarcopenia Muscle 2025) and isoform 2/ASB2α (Guibal et al. J Biol Chem 2002; Metais et al, Circ Res 2018; Maire et al, Nat Commun 2024). Since these proteins exhibited different functional domains and have different substrates, the authors should investigate which isoform is expressed in ILC1s. This is of importance since the exon of ASB2 that is flanked with loxP sequences in the conditional knockout mouse models is common to both isoforms.

3. The authors have to validate the loss of ASB2 expression in ASB2 knockout cells.

4. Ncr1-Cre heterozygous mice must be used as controls in all experiments because NKp46-expressing cells from heterozygous Ncr1-Cre mice were shown to display a significant reduction of NKp46 expression compared to those of mice that do not express the Cre, likely with consequences on ILC1 sorting. In addition, this may impact ILC1 activity as previously demonstrated in NK cells (Narni-Mancinelli, Science, 2012). Asb2fl/fl mice cannot be used as controls.

5. GO enrichment analysis showed that genes involved in the negative regulation of cell adhesion are at the top of the list of genes up-regulated in liver ILC1s of Ncr1Cre/+Asb2fl/fl mice. This is not mentioned, explored, nor discussed in the manuscript although ASB2 isoform 2 (ASB2α) was previously shown to regulate cell motility in immune cells (Lamsoul et al, Blood 2013; Maire et al, Nature Communications 2024). The authors should therefore investigate whether ASB2 loss in ILC1s has an impact on their homing and residency in the liver or in their distribution in other tissue.

6. In the Volcano plots showed in Fig 3a, it seems that some of the proteins that were the most highly differentially expressed were omitted. What are the values provided in the proteomic table for protein abundances in the ILC1 samples? How were the missing values treated? What is the statistical test used to determine the differentially expressed proteins in the 2 KO vs the 3 control ILC1 samples?
Names of the differentially expressed genes / proteins found in the transcriptomic and proteomic analyses should be provided in a supplementary table. Genes of enriched pathways (GO transcriptomic analysis) should be provided in a supplementary table.

7. The functional link between ASB2 and ILC1 biology is missing and should be investigated. This requires identification of the ASB2 isoform that is expressed in liver ILC1s and elucidation of downstream mechanisms controlling ILC1 properties, including identification of the substrates whose ubiquitination mediates downstream events.

8. In its current form, this manuscript reveals a profound alteration of gene and protein expression following ASB2 deletion in liver ILC1s, associated with impaired cell survival.
The authors concluded from the results shown in figure 4d and 5g that there is a functional link between ASB2 and the viability or the lipid content of ILC1s. However, these results are questionable.
Indeed, the authors represented fold-changes (viable cells in liver ILC1s between Asb2fl/fl and Ncr1Cre/+Asb2fl/fl mice and lipid contents in liver ILC1s between Ncr1Cre/+Asb2fl/fl and Asb2fl/fl mice in tumor-bearing and non-tumor-bearing conditions, respectively), with statistical analyzes. How were these fold-changes calculated, particularly with different numbers of samples? If these fold-changes were calculated to relative means, statistical analyses could not be performed.
Regarding the panel g of figure 5, the lipid contents in liver ILC1s of Ncr1Cre/+Asb2fl/fl and control mice in tumor-bearing and non-tumor-bearing conditions should also be provided.

9. The gender of the mice used in all the experiments is not specified. This is of major importance in many experiments, at least to study the impact of ASB2 deficiency in the anti-tumor function of ILC1s.
Although it is indicated in the Methods section that “Mice were age-matched and sex-matched for each experiment”, only 6 mice of each genotype were injected with MC38 cells in two independent experiments. This should be clarified.

10. Throughout the manuscript, the authors indicated that statistical significance was performed using a two-tailed unpaired Student t test. However, this test can be performed only if each of the populations being compared follows a normal distribution. For sure, this cannot be stated from only 3 or 4 experiments. This is of importance since it questioned the statistical significance of almost all data.

Minor comments:

1. Some typographical errors have to be corrected.

2. The subtitle “ASB2 is specifically expressed in adult liver ILC1s” is an overinterpretation / simplification of the results.

3. Samples from Asb2fl/fl mice should not be labeled as “WT”.

4. Related to data shown in figure 4f,g, treatment of ILC1s from control mice with palmitic acid and steric acid with or without A922500 should be performed.

5. The survival curves of Ncr1Cre/+Asb2fl/fl and Asb2fl/fl mice that received MC38 cells indicate that almost all mice are dying during the experiments. Is it ethically acceptable?

Version 1:

Reviewer comments:

Reviewer #1

(Remarks to the Author)
The authors have addressed my concerns.

Reviewer #2

(Remarks to the Author)
The revised manuscript has been improved, and the authors have addressed most of the previous comments. However, some issues remain and should be resolved before publication.

1. In the revised manuscript and response letter, the authors added data describing Asb2 expression across multiple immune cell populations and tissues in the reporter mouse, which is helpful for the characterization of this newly generated strain. However, the new reporter mouse model has not been validated to great extent. It is important to confirm that the fluorescent reporter faithfully reflects endogenous Asb2 expression which has not been provided.
2. Page 4, lines 91-95, the authors report that tamoxifen-induced deletion in Ncr1CreERT2/+Asb2fl/fl mice reduces liver ILC1s (Figure 2d,e), but then refer VavCre/+Asb2fl/fl mice to assess other lymphocyte populations (supplementary Figure 3c). The purpose of the VavCre model appears to be that Asb2 does not affect other lymphocyte populations however the authors should also provide the ILC1 data in the same model.
3. There appear to be multiple errors in supplementary figure citations throughout the revised manuscript.

For example:
Results section 2, “ASB2 supports the maintenance of adult liver ILC1s,” Supplementary Fig. 2d, 2e appears to be incorrectly cited and should likely be Supplementary Fig. 3c, 3d-e, while
Supplementary Fig. 3c should likely be Supplementary Fig. 4c.

Results section 3, “ASB2 inhibits lipid storage in ILC1s,” references to Supplementary Fig. 4 appear to correspond to Supplementary Fig. 5.

Results section 4, “ASB2 enhances the anti-tumor activity of liver ILC1,” references to Supplementary Fig. 5 appear to correspond to Supplementary Fig. 6, and Supplementary Fig. 6 should likely be Supplementary Fig. 7.

The authors should recheck all figure citations to ensure they are correct.

Reviewer #3

(Remarks to the Author)
I co-reviewed this manuscript with one of the reviewers who provided the listed reports. This is part of the Nature Communications initiative to facilitate training in peer review and to provide appropriate recognition for Early Career Researchers who co-review manuscripts.

Reviewer #4

(Remarks to the Author)
The manuscript has improved significantly and the authors critically addressed many points I raised. However, few issues still need to be fixed.

Major issues:

1. From the data provided in the new Fig. 1k, the authors concluded that “liver ILC1 exclusively express the ASB2alpha isoform”. However, it is not clear how the relative expression between the two ASB2 isoforms in ILC1s and cNKs was calculated. This is absolutely required to support their conclusion.

2. There is still a major gap in mechanistic part of the revised manuscript. The authors showed co-immunoprecipitation of ASB2alpha with DGAT1 in transfected HEK cells, indicating an association between the two proteins (and not a direct interaction as concluded page 5, line 141). Because DGAT1 is more abundant in ASB2-deficient cells and because ASB2alpha is known to exert its effects through polyubiquitination and degradation of its substrates, the authors should determine whether DGAT1 is a substrate of ASB2alpha ubiquitin ligase activity in ILC1s. If not, the functional link between ASB2alpha, DGAT1 and lipid metabolism in ILC1s still remains elusive.

Minor comments:

1. In the description of the data obtained with the ASB2-EGFP mouse model, the authors have to clearly indicate in the result section that this model does not discriminate between the expression of the two ASB2 isoforms.

2. The authors should tone down their statement that ASB2 may be dispensable for early development of ILC1s because ASB2 was barely expressed in CD45+Lin− mixed progenitors and PD-1+ ILC precursors. Low expression does not mean no function!

3. In the main text, some figures are misnumbered.

Version 2:

Reviewer comments:

Reviewer #2

(Remarks to the Author)
Overall, this manuscript is significantly improved by the modifications.

Reviewer #3

(Remarks to the Author)
I co-reviewed this manuscript with one of the reviewers who provided the listed reports. This is part of the Nature Communications initiative to facilitate training in peer review and to provide appropriate recognition for Early Career Researchers who co-review manuscripts.

Reviewer #4

(Remarks to the Author)
The authors adequately addressed the different issues.

Open Access This Peer Review File is licensed under a Creative Commons Attribution 4.0 International License, which permits use, sharing, adaptation, distribution and reproduction in any medium or format, as long as you give appropriate credit to the original author(s) and the source, provide a link to the Creative Commons license, and indicate if changes were made.
In cases where reviewers are anonymous, credit should be given to 'Anonymous Referee' and the source.
The images or other third party material in this Peer Review File are included in the article's Creative Commons license, unless indicated otherwise in a credit line to the material. If material is not included in the article's Creative Commons license and your intended use is not permitted by statutory regulation or exceeds the permitted use, you will need to obtain permission directly from the copyright holder.
To view a copy of this license, visit https://creativecommons.org/licenses/by/4.0/

Point-by-point reply to the reviewers' comments (in blue)

Review # 1

In this manuscript, Bao et al. reported that ASB2 is specifically expressed in liver ILC1s and knockout of ASB2 in ILCs and NK cells reduces ILC1 cell population in the liver. Mechanistically, they showed that loss of Asb1 induces lipid accumulation, resulting in cell death. They demonstrated that DGAT1 inhibitor significantly reduces lipid accumulation and rescues cell death in Asb1 knockout ILCs. Finally, they showed that Asb1 knockout promotes colorectal cancer metastasis to liver and DGAT1 inhibitor reduces cancer growth. While the authors presented solid evidence that ASB2 is important for ILC1 survival, the mechanistic studies are not convincing. There are major gaps in their claimed mechanisms and the data presented do not fully support the conclusions.

REPLY:

We thank the reviewer for the insightful suggestions, which have enabled us to deepen our investigation of the mechanisms underlying our original observations.

1.The authors claimed that there is increased lipid accumulation in the cells in the absence of Asb2. This conclusion is only based on BODIPY staining, which is not sufficient. They showed directly measure lipid levels in the cells. They claimed that lipid accumulation induces cell damage and apoptosis. It is known that free fatty acids and cholesterol can induce toxicity and cell death. What kind of lipids are increased in Asb2 knockout cells?

REPLY:

Thanks for the valuable comment. To identify the lipid species responsible for lipid accumulation in ASB2-deficient ($Ncr1^{Cre/+}Asb2^{fl/fl}$) ILC1s, we performed flow cytometry using specific probes for different kinds of lipids. First, we measured intracellular levels of C12, C16, cholesterol and BODIPY-493/503 straining in fixed cells. We observed a significant decrease in intracellular C12 fatty acid levels, a moderate increase in cholesterol levels, and no alteration in C16 fatty acids levels, alongside an increase in BODIPY 493/503 staining, in ASB2-deficient ILC1s **(Fig. R1a, revised Supplementary Fig. 5d in the manuscript)**. Furthermore, live cell uptake assays with BODIPY C12 and C16 showed no difference between control and ASB2-deficient ILC1s (**Fig. R1b, revised Supplementary Fig. 5e in the manuscript**). These results combined with the upregulated expression of DGAT1 (**Fig 3c**), a key enzyme catalyzing triglyceride synthesis from fatty acids, we propose that C12 fatty acids are likely consumed and utilized for DGAT1-mediated neutral lipid production. Meanwhile, the mild elevation in cholesterol may also potentially contribute to the increased neutral lipid pool.

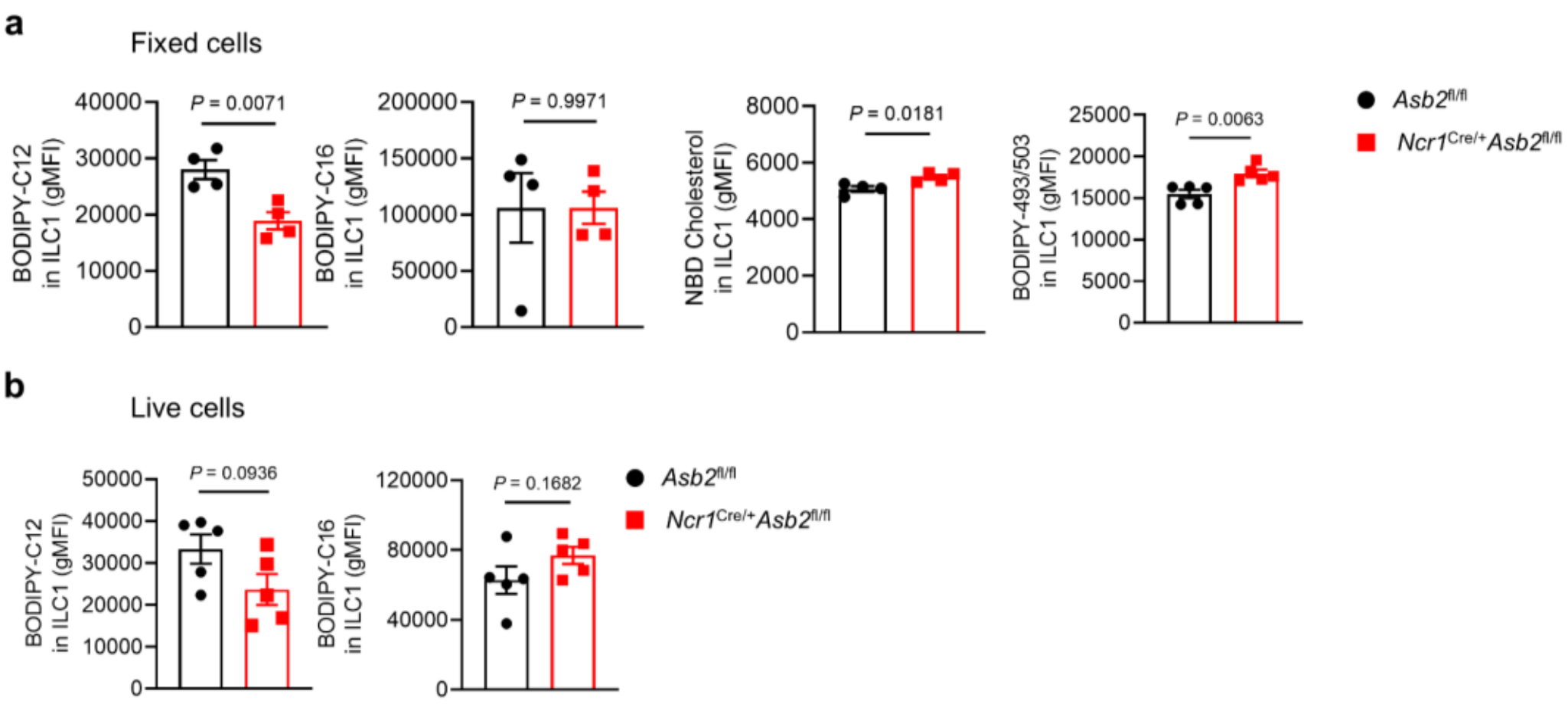

Fig R1. ASB2 modulates lipid contents but has no effect on lipid uptake in ILC1s.

(a) gMFI of C12 (n = 4 per group), C16 (n = 4 per group), NBD cholesterol (n = 4 per group) and BODIPY-493/503 straining (n = 5 per group) in fixed ILC1s from adult *Ncr1*$^{Cre/+}$*Asb2*$^{fl/fl}$ and *Asb2*$^{fl/fl}$ mice. **(b)** gMFI of C12 (n = 5 per group), C16 (n = 5 per group) in liver live ILC1s from adult *Ncr1*$^{Cre/+}$*Asb2*$^{fl/fl}$ and *Asb2*$^{fl/fl}$ mice. Data are from one representative experiment of two independent experiments**.** Samples were compared using two-tailed unpaired Student's *t* tests and were presented as means ± SEM.

2. The authors showed *Asb2* knockout increases FABP1 and DGAT1 expression, which they claimed to be responsible for lipid accumulation. They did not show direct evidence that Asb2 knockout indeed increase fatty acid uptake and lipid biosynthesis.

REPLY:

Thanks for pointing out this important issue. As mentioned above (reply to comment #1), we performed lipid uptake assays in live cells and found no change in C12 and C16 fatty acid uptake in ILC1s from *Ncr1*$^{Cre/+}$*Asb2*$^{fl/fl}$ mice **(Fig. R1b, revised Supplementary Fig. 5e in the manuscript)**, indicating that ASB2 does not affect cellular fatty acid uptake despite a slight increase in FABP1 expression. However, intracellular C12 content was significantly decreased **(Fig. R1, revised Supplementary Fig. 5d in the manuscript)**, accompanied by markedly increased DGAT1 expression, strongly suggesting that fatty acids are consumed for enhanced DGAT1-dependent triglyceride synthesis. Importantly, our rescue experiments using a DGAT1-inhibitor further support that DGAT1-dependent triglyceride accumulation is a key mechanism underlying the disturbed lipid metabolism in ASB2-deficient ILC1s **(revised Fig. 4f)**. Taken together, these results suggest that ASB2 deficiency promotes DGAT1-mediated neutral lipid biosynthesis rather than increasing bulk fatty acid uptake.

3. The authors need to provide direct evidence that DGAT1 inhibitor treatment indeed reduces cellular lipid levels.

REPLY:

We sincerely appreciate the reviewer's important suggestion. According to the suggestion, we treated control (*Asb2*$^{fl/fl}$) and ASB2-deficient (*Ncr1*$^{Cre/+}$*Asb2*$^{fl/fl}$) ILC1s with the DGAT1 inhibitor A922500, and measured intracellular lipid levels using BODIPY-493/503 staining. We found that DGAT1 inhibition significantly reduced lipid content in both control and ASB2-deficient ILC1s (**Fig. R2, revised Fig. 4e in the manuscript)**. Notably, following A922500 treatment, the significant difference in lipid levels between control and ASB2-deficient ILC1s was no longer observed **(revised Fig. 4f)**.

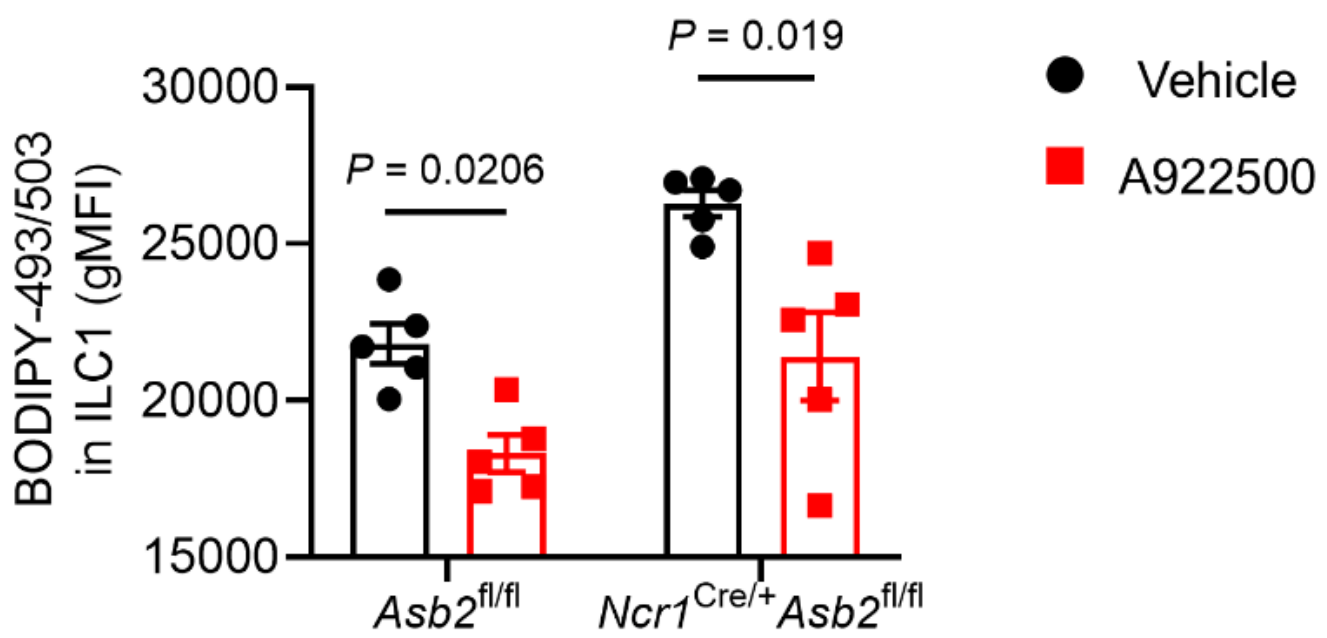

Figure R2. Treatment with the DGAT1 inhibitor A922500 reduces cellular lipid levels of ILC1s. gMFI of BODIPY-493/503 staining in ILC1s from adult *Ncr1*$^{Cre/+}$*Asb2*$^{fl/fl}$ and *Asb2*$^{fl/fl}$ mice, cultured with or without 3 μM A922500 for 24 h (n = 5 per group). Data are from one representative experiment of two independent experiments. Samples were compared using two-way ANOVA and are presented as means ± SEM.

4. In Fig. 6, the authors claimed that DGAT1 inhibitor treatment reduces tumor growth through modulating ILC1s. Does DGAT1 inhibitor itself have any effect on tumor growth? They need to test if tumor growth is also inhibited by DGAT1 inhibitor in the tumor cells without endogenous DGAT1.

REPLY:

Thanks for pointing out this important issue. To investigate whether the DGAT1 inhibitor A922500 affects MC38-luc tumor growth, we first performed in vitro experiments with different concentrations of A922500. In vitro, the DGAT1 inhibitor A922500 was used at 3 μM to treat ILC1s, a concentration that significantly enhanced their viability **(revised Fig. 4f)**, while we treated tumor cells with 5, 10, and 20 μM to evaluate its potential toxic effect. We found that 5 μM A922500 had no effect on MC38-luc cell growth, whereas 10 μM and 20 μM A922500 significantly inhibited cell growth (**Fig. R3a, shown as below**). More importantly, we intrasplenic injected MC38-luc cells into immunodeficient NCG mice, which lack ILC1s and other lymphocytes, and treated them with the same dose of A922500 used in the therapeutic experiments for WT mice (10 mg/kg A922500

every 3 days). We found that this treatment did not significantly affect MC38-luc tumor growth in the NCG mice (**Fig, R3b, shown as below**). Although a previous study used 120 mg/kg of A922500 to treat mice and observed suppressed tumor growth (Cell Metab, 2020; 32:229-242), the dose they used was much higher than that in our study. Thus, our current results clearly demonstrate that the inhibitor under our experimental conditions does not directly suppress tumor cells.

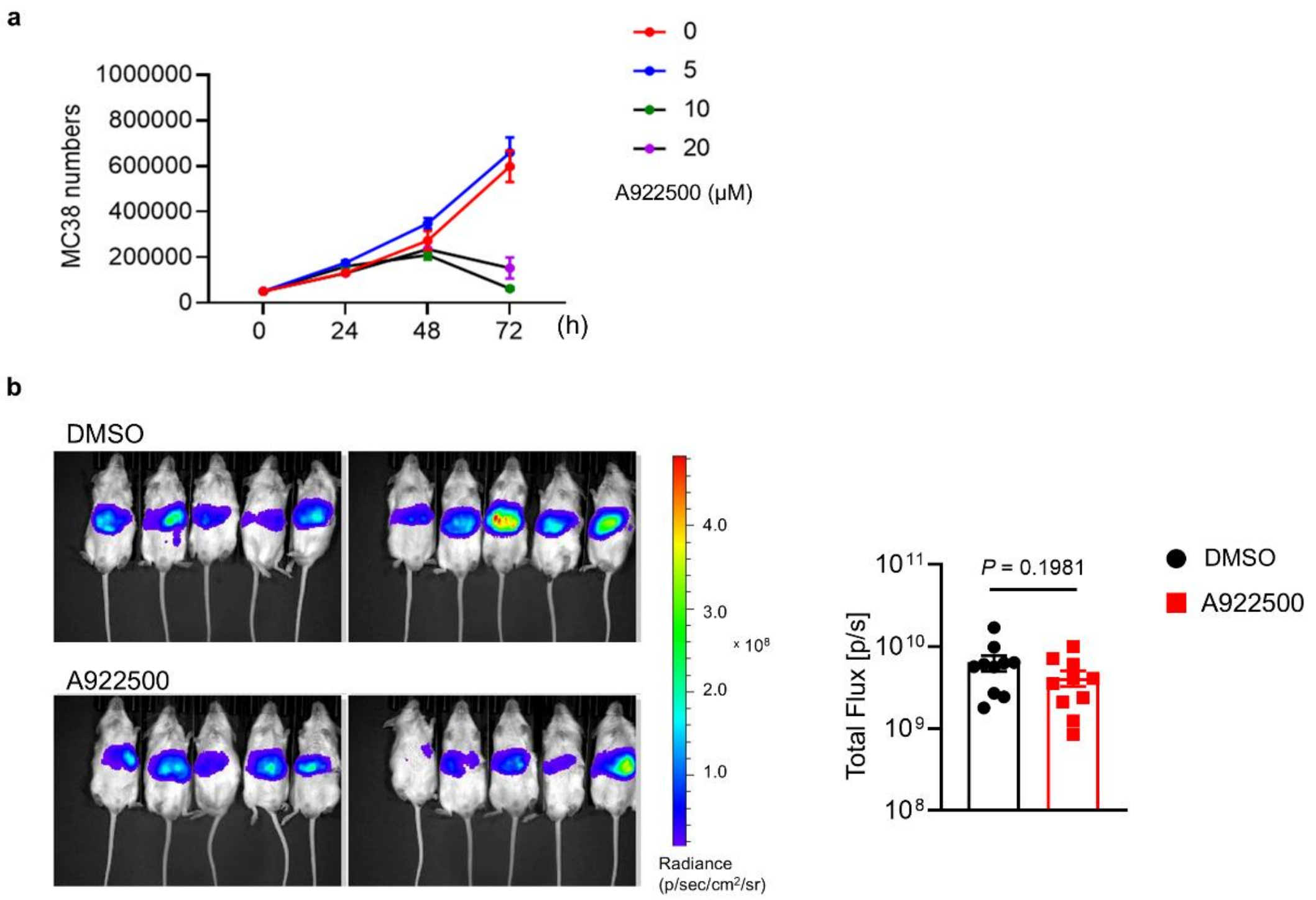

Fig R3. Evaluation of the DGAT1 inhibitor A922500 on tumor cell growth.
(a) The number of MC38-luc cells treated with different concentrations of A922500 for 24, 48 and 72 h. **(b)** NCG mice were intraperitoneally injected with DMSO or A922500 (10 mg/kg) every three days after initial intrasplenic injection of 2×10^5 MC38-luc cells. In vivo bioluminescence imaging of tumor metastases on day 10 (left) and quantification of the radiance values of tumor metastases on day 10 (right) (n = 10 per group). Samples were compared using two-tailed unpaired Student's t tests and were presented as means ± SEM.

Minor points:

1. Line 132-133, Fig. 4a and 4b showed PA treated cells, not SA. Fig. 4c and 4d showed SA treated cells, not PA.

REPLY:

We greatly appreciate you for carefully identifying this mistake. We have corrected the error accordingly (**revised Page 6, Line 149-151, and Fig. 4a-4d**).

2. Typo: Line 41, dirven should be driven.

REPLY：

We greatly appreciate you for pointing out this typo. We have corrected “dirven” to “driven” (**revised Page 2, Line 41**).

Review # 2

This manuscript identifies a role for ABS2 in limiting lipid accumulation that induces apoptosis of adult liver ILC1s, thereby restraining the progression of liver metastasis. The study provides a novel mechanistic link between lipid metabolism and the maintenance of adult liver ILC1s. While the work is original and potentially impactful, additional experimental evidence and greater methodological detail are needed to strengthen the conclusions and ensure reproducibility. In addition, it generates a new tool that will be of value to the field. With revision, the study could make a valuable contribution to our understanding of the metabolic regulation of liver-resident ILCs. The authors demonstrate that ASB2 reduces lipid accumulation at steady-state and in tumor microenvironment, thereby protecting ILC1s from lipid-induced apoptosis. Loss of ASB2 compromises ILC1 survival and correlates with enhanced metastatic progression. Integration of sequencing data with in vivo mouse experiments supports the proposed mechanism that ASB2 maintains lipid metabolic balance in the adult liver. The work broadens our understanding of how lipid metabolism influences the maintenance and function of adult liver ILC1s, an area that remains relatively underexplored. Overall, this is a solid manuscript which makes a novel contribution to the field and would be strengthened by substantial revisions and clarification.

REPLY:

We thank the reviewer for the interest in our study and helpful comments. Our manuscript has been revised accordingly and greatly improved.

1. The authors have focused only on mature ILC1 in the liver. They have generated a very nice reporter mouse but have provided limited characterization of this new strain. Could the authors provide a more thorough characterization of this strain across multiple cell types (particularly other ILCs, NKT cells, MAIT cells and adaptive immune cells) and multiple tissues (e.g., gut, lung, spleen) to assess tissue specificity (as supplementary data). Analysis of the expression of the reporter relative to gene expression is also required.

REPLY:

Thanks for the construction suggestions. Using *Asb2*^{EGFP} mice, we performed extensive flow cytometric analysis of diverse immune cell populations, including ILCs, NKT, CD4^{+} T, CD8^{+} T, MAIT, and B cells, from multiple tissues (liver, spleen, small intestine, and lung). We found that ASB2 was highly expressed by the majority of tissue-resident ILC1s and NKT cells in the liver, with low to no expression by circulating lymphocytes, such as CD4^{+} T, CD8^{+} T, B, and conventional

NK (cNK) cells in both liver and spleen. Besides, MAIT cells also showed low expression. In the lung, nearly half of ILC2s expressed ASB2, while cNK cells, consistent with their counterparts in the spleen and liver, remained negative. Notably, ASB2 was broadly expressed in intestinal lymphocytes. ILC1s, ILC2s, and ILC3s in the small intestine were almost all positive, and about half of cNKs were also positive. This relatively broad expression in the intestine may be induced by the unique tissue microenvironment. This comprehensive analysis of ASB2 expression has been integrated into the revised manuscript (**Fig. R4, revised Supplementary Fig.1c and Supplementary Fig.2a-d in the manuscript)**.

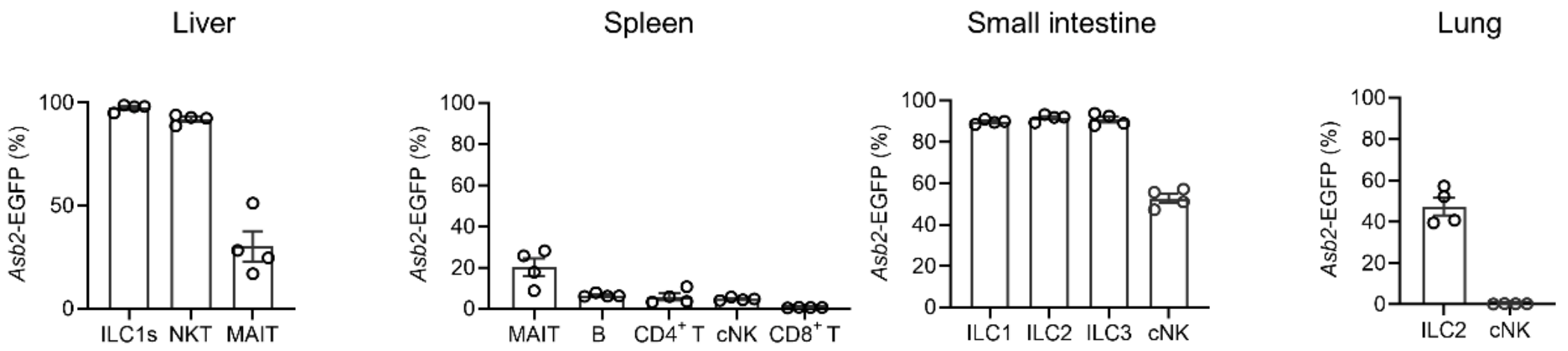

Fig. R4. Expression profile of ASB2 in multiple lymphocyte populations across different tissues.

Asb2 expression in various lymphocyte populations across tissues of adult *Asb2*^{EGFP} mice by flow cytometric analysis (n = 4 per group). Data are from one representative experiment of two independent experiments. Samples were presented as means ± SEM.

2. What is the expression of ASB2 in early bone marrow progenitors? and is expression required for development of ILC?

REPLY:

Thanks for pointing out this important issue. We examined *Asb2* expression in CD45$^+$Lin$^-$ cells, a mixed population of early progenitors, and ILCPs in the bone marrow, and found that *Asb2* was barely expressed in these progenitor cells, suggesting that *Asb2* is unlikely to be required for early development of ILCs. We have added these flow cytometric data of *Asb2* expression in early bone marrow progenitors in current version (**Fig. R5, revised Supplementary Fig. 3d, e in the manuscript**).

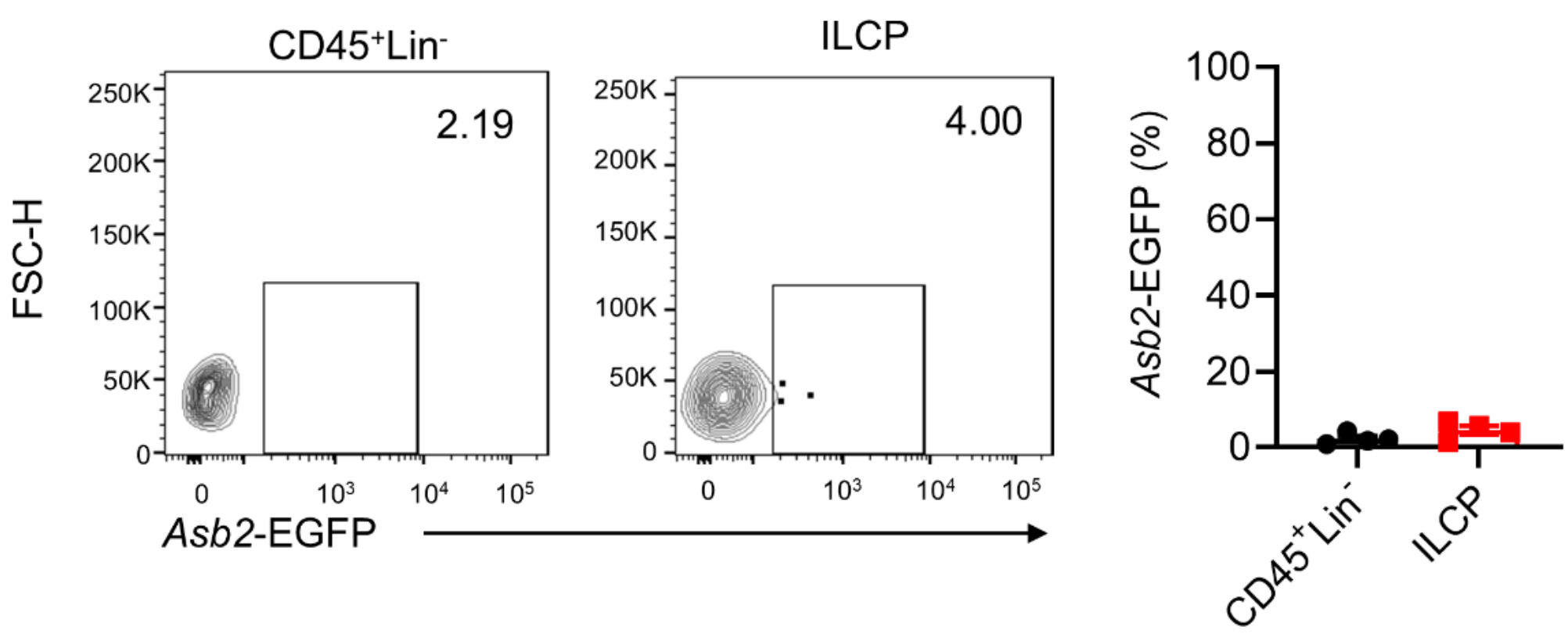

Fig. R5. CD45$^+$Lin$^-$ cell and ILCPs in the bone marrow.

Flow cytometry analysis (left) and quantification (right) of *Asb2* expression in CD45$^+$Lin$^-$ cells and ILCPs (CD45$^+$Lin$^-$CD127$^+$Flt3$^-$α4β7$^+$CD25$^-$PD-1$^+$) in the bone marrow of adult *Asb2*^{EGFP} mice. (*n* = 4 per group). Data are from one representative experiment of two independent experiments. Samples were presented as means ± SEM.

3. Among mature cells, little functional data is provided. The authors should provide data on the functional effector changes (e.g., granzyme B and IFN-γ production), if any, induced by loss of ASB2 in addition their assessment of cell viability.

REPLY:

Thanks for the insightful suggestion. We had showed the effects of ASB2 on the expression levels of activating molecules (CD226, NKG2D), inhibitory molecules (TIM-3, TIGIT, PD-1), and functional molecules (granzyme A, granzyme B, granzyme C, IFN-γ, TNF-α, CD107a) in liver ILC1s in current version **(Fig. R6, revised Supplementary Fig. 6a, b in the manuscript)**.

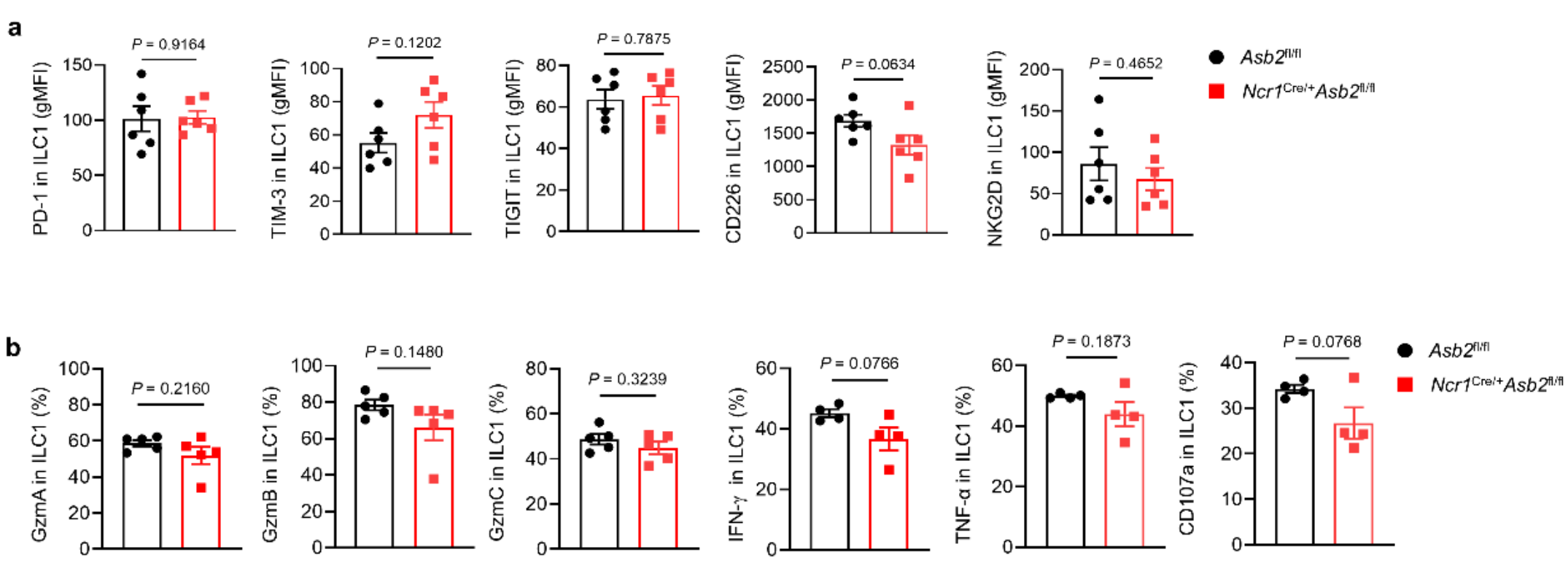

R6. ASB2 deficiency does not affect ILC1 function at steady state. (a) Expression of activating and inhibitory receptors on liver ILC1s from *Ncr1*$^{Cre/+}$*Asb2*$^{fl/fl}$ and *Asb2*$^{fl/fl}$ mice (*n* = 6 per group). **(b)** Expression of Granzyme A (*n* = 5), Granzyme B (*n* = 5), Granzyme C (*n* = 5), interferon-γ (IFN-

γ) (n = 4), Tumor Necrosis Factor alpha (TNF-α) (n = 4) and CD107a (n = 4) by liver ILC1s from *Ncr1*$^{Cre/+}$*Asb2*$^{fl/fl}$ and *Asb2*$^{fl/fl}$ mice. Data are from one representative experiment of two independent experiments. Samples were compared using two-tailed unpaired Student's *t* tests and are presented as means ± SEM.

4. What is the expression of ASB2 in human tissues and tumors and potential correlation with ILC1 frequency and prognosis in human cancers or liver metastases, for example using TCGA datasets.

REPLY:

Thanks for the helpful advice. We analyzed *ASB2* expression across multiple human tissues and tumors using GEPIA3, which covers pan-cancer databases including TCGA, GTEx, PCAWG, and ICGC. In many tumor types, including Liver hepatocellular carcinoma (LIHC), there was no significant difference in *ASB2* expression between tumor tissues and normal tissues. However, ASB2 expression was significantly downregulated in Uterine carcinosarcoma (UCS), Bladder Urothelial Carcinoma (BLCA), Colon adenocarcinoma (COAD), Glioblastoma multiforme (GBM), Prostate adenocarcinoma (PRAD), Rectum adenocarcinoma (READ), Sarcoma (SARC), and Uterine Corpus Endometrial Carcinoma (UCEC) tissues. In contrast, *ASB2* expression was markedly upregulated in Pancreatic adenocarcinoma (PAAD) tissues (**Fig. R7a, revised Supplementary Fig. 7a in the manuscript**). We also analyzed the correlation between *ASB2* expression and the expression of ILC1 signature genes (*NCAM1*, *NCR1*, *GZMK*, *RGS1*, *ZNF683*, *ITGA1*) in normal liver, LIHC peritumor, and LIHC tumor tissues, and found that *ASB2* expression was positively correlated with the expression of ILC1 signature genes (**Fig. R7b, revised Supplementary Fig. 7b in the manuscript**). Additionally, we observed that LIHC patients with high *ASB2* expression exhibited a higher survival rate compared with those with low *ASB2* expression (**Fig. R7c, revised Supplementary Fig. 7c in the manuscript**). These results support the potential clinical relevance of *ASB2* in human liver cancer.

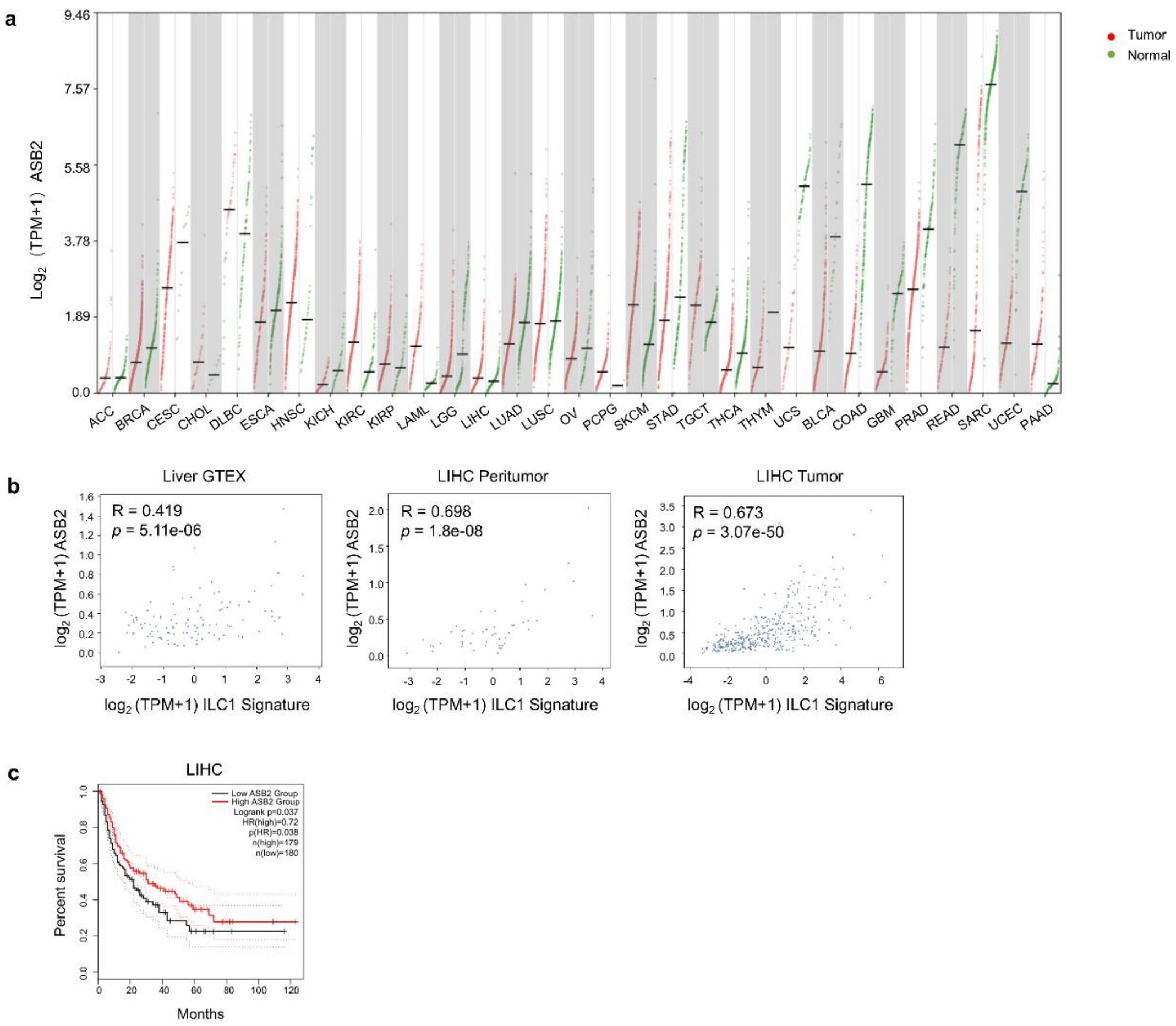

Fig. R7. Clinical relevance of ASB2 expression in human cancers.

(a) Expression levels of *ASB2* in normal tissues and tumor tissues using GEPIA3. **(b)** Correlation between *ASB2* expression and the expression of ILC1 signature genes (*NCAM1*, *NCR1*, *GZMK*, *RGS1*, *ZNF683*, *ITGA1*) in normal liver, LIHC peritumor, and LIHC tumor tissues using GEPIA3. **(c)** Correlation between *ASB2* expression and patient prognosis in LIHC. LIHC: liver hepatocellular carcinoma using GEPIA3.

5. The authors should provide more detailed information on in vitro cell culture conditions, and lipid quantification protocols. Additional methodological description is required for analysis of the liver ILC RNA sequencing. These data do not appear to have been provided in a publicly accessible database. This is important that this is available on publication of the manuscript.

REPLY:

We sincerely appreciate the reviewer's constructive comments on improving the methodology details. According to the suggestions, we have substantially supplemented the Methods section with detailed experimental procedures, including in vitro cell culture conditions **(revised Page 15, Line 474-478)**, lipid quantification protocols **(revised Page 15, Line 480-487)**, and bioinformatics analysis for liver ILC RNA sequencing (**revised Page 16 and 17, Line 521-542**). In addition, RNA-

seq for liver ILC1s isolated from *Asb2*$^{fl/fl}$ and *Asb2* conditional knockout have been uploaded to the Genome Sequence Archive (GSA: CRA030972), and will be released to the public at the time of publication.

6. The expansion of the CD49a$^+$CD49b$^+$ population within tumors has been reported in multiple studies. Have the authors observed a similar expansion in their tumor model? What are the lipid levels within this population, and are they affected by *Asb2*?

REPLY:

Thanks for the comment. Increasing studies have demonstrated a substantial expansion of the CD49a$^+$CD49b$^+$ cell subset in the MC38 liver metastasis model. We analyzed *Asb2*$^{fl/fl}$ mice without tumor burden and those at 14 days post-tumor inoculation, and consistently observed a marked expansion of the CD49a$^+$CD49b$^+$ cell subset in the metastatic liver (**Fig, R8a, shown as below**). Meanwhile, the lipid content in the CD49a$^+$CD49b$^+$ cell subset was even slightly higher than that in ILC1s (**Fig, R8b, shown as below**). Furthermore, the lipid content in the CD49a$^+$CD49b$^+$ cell subset from metastatic liver of *Ncr1*$^{Cre/+}$*Asb2*$^{fl/fl}$ mice was further increased compared to that of *Asb2*$^{fl/fl}$ mice (**Fig, R8c, shown as below**). This finding suggests that ASB2 may also regulates the lipid metabolism of the CD49a$^+$CD49b$^+$ subset, analogous to its function in ILC1s.

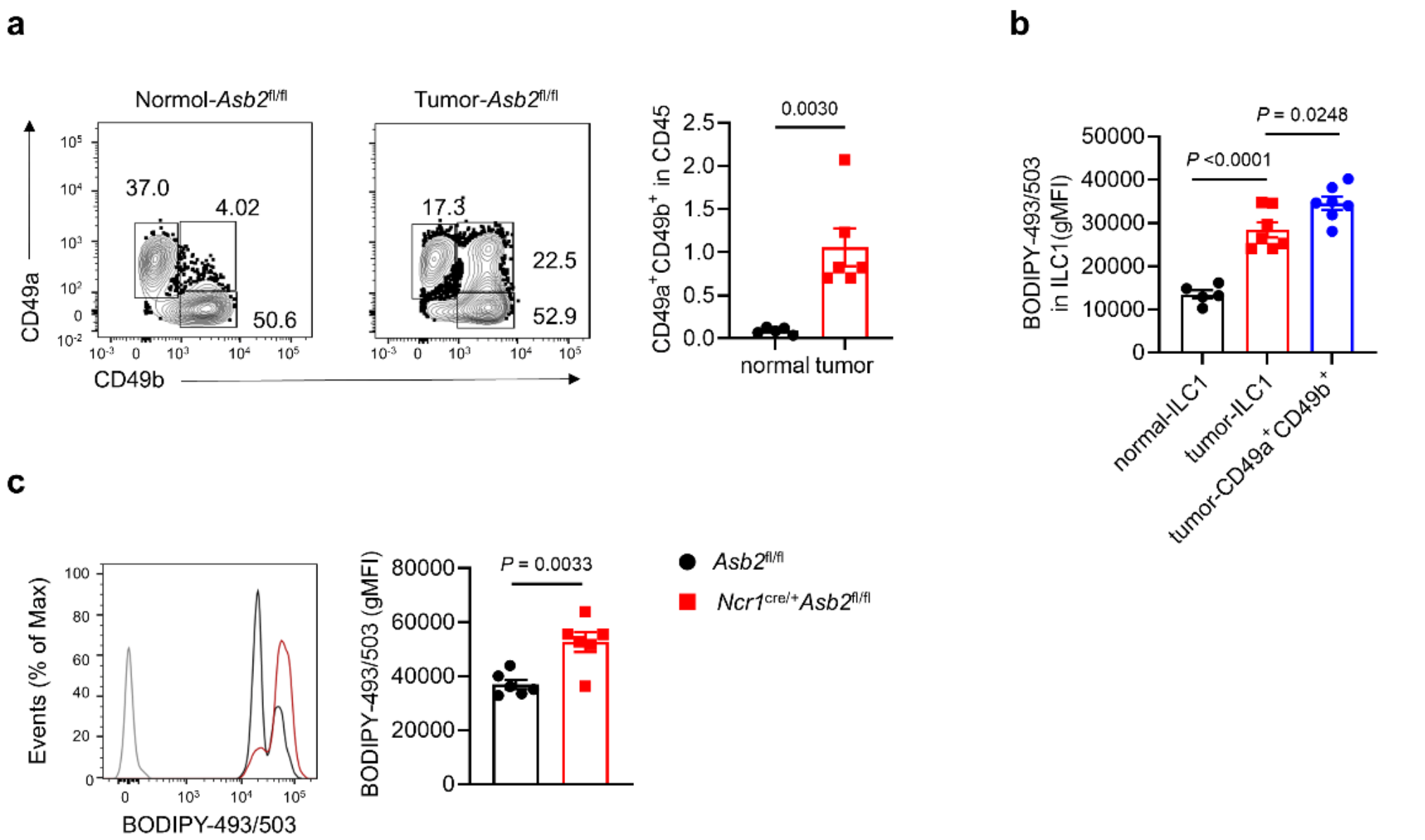

Fig.R8. Expansion and lipid metabolism of the CD49a$^+$CD49b$^+$ subset in metastatic liver.

(a) Representative flow cytometry plots (left) and quantification (right) of NK1.1$^+$NKp46$^+$TCRβ$^-$CD49a$^+$CD49b$^+$ NK cells in CD45$^+$ cells from *Asb2*$^{fl/fl}$ mice on day 14 after intrasplenic injection of 2×10^5 MC38-luc cells, (*n* = 5 normal group, *n* = 6 tumor group). **(b)** gMFI of BODIPY-493/503 staining in ILC1s and CD49a$^+$CD49b$^+$ NK cells from C57BL/6J on day 21 after intrasplenic injection of 5×10^5 MC38-luc cells (*n* = 5 normal-ILC1 group, *n* = 7 tumor-ILC1 and tumor CD49a$^+$CD49b$^+$ group). **(c)** gMFI of BODIPY-493/503 staining in CD49a$^+$CD49b$^+$ NK

cells from *Ncr1*$^{Cre/+}$*Asb2*$^{fl/fl}$ and *Asb2*$^{fl/fl}$ mice on day 14 after intrasplenic injection of 2×10^5 MC38-luc cells (n = 6 per group). Samples were compared using one-way ANOVA **(b)** or two-tailed unpaired Student's t tests **(a, c)**. Data were presented as means ± SEM.

7. In addition to the tumor tissue, did the authors examine the adjacent liver tissue? If so, what are the ILC1 numbers and lipid levels in the adjacent liver compared with the tumor site?

REPLY:
Thanks for this suggestion. For experimental rigor, it would be ideal to compare the number and lipid content of ILC1s between tumor and adjacent liver tissues. However, in the MC38 liver metastasis model, tumor lesions are diffusely distributed throughout the liver and thus difficult to be macroscopically distinguished from tumor-free tissues, rendering this experiment technically unfeasible.

Reviewer #3 (Remarks to the Author):
I co-reviewed this manuscript with one of the reviewers who provided the listed reports. This is part of the Nature Communications initiative to facilitate training in peer review and to provide appropriate recognition for Early Career Researchers who co-review manuscripts.
Reviewer #4
Reviewer #4 [asb2] (Remarks to the Author):
Through the analyses of publicly available single cell and bulk RNA-seq data, and the use of an Asb2-EGFP mouse, the authors showed that ASB2 is expressed in mouse adult liver ILC1s. Transcriptomic and proteomic analyses reveal a profound alteration of gene and protein expression following ASB2 deletion in liver ILC1s, associated with impaired cell survival. They further propose that deletion of ASB2 increase lipid storage in liver ILC1, leading to reduced ILC1 survival and increased tumor burden in the liver after intrasplenic injection of MC38 cells.
There are many issues that need to be fixed to enable authors to fully support the conclusions made in the manuscript.

REPLY:
We thank both you and Reviewer #3 for the collaborative effort in evaluating our study. We appreciate the time and expertise you have contributed. Your insightful suggestions are helpful for us to improve the manuscript.

1. Although interesting, 1) the finding that ASB2 is expressed in ILC1 is not new (McFarland et al, Immunity 2021; Frey et al, iScience 2025), but this is not mentioned in the manuscript. 2) In addition, the authors reported specific expression of ASB2 in adult liver ILC1s compared to in other tissue-resident ILC1s, including small intestine. These results contrast with those mentioned above

(McFarland et al, Immunity 2021) that showed that ILC1s in the small intestine selectively expressed ASB2 relative to the liver, visceral adipose tissue, salivary glands and uterus. Since the comparative analysis of ASB2 expression in different populations / tissues was performed using 3 different datasets and thus requires data normalization (Fig1 h), complementary approaches should be used to analyze the expression of ASB2 in ILC1s of different mouse tissues. 3) The authors used "Asb2-EGFP mice" but there is no description of these mice throughout the manuscript. A clear description of the construct is absolutely required to evaluate the data obtained with these mice.

REPLY：

Thanks for pointing out these important issues.

1) Regarding the expression of ASB2 in ILC1s, we fully agree that this has been reported in previous studies (McFarland et al., Immunity, 2021; Frey et al., iScience, 2025). We have now cited these two important studies in the revised manuscript to acknowledge their contribution in the field **(revised Page 8, Line 210-212).**

2) Concerning ASB2 expression in ILC1s across different organs, we agree that previous transcriptomic analyses showed that intestinal ILC1s display higher ASB2 expression levels than liver ILC1s when comparing signal intensities directly. Our data are not contradictory to these findings. In our current study, we focused on the relative expression ratio of ASB2 in ILC1s versus cNK cells within the same tissue, rather than the absolute expression level across tissues, showing the highest ratio in the liver compared with other tissues. In the revised manuscript, we additionally provide the data showing ASB2 expression in different lymphoid subsets across several tissues by using $Asb2^{EGFP}$ mice. We found that both liver and intestinal ILC1s are almost uniformly positive for ASB2. While cNK cells in the liver, spleen, and lung rarely expressed ASB2, half of cNK cells in the small intestine were ASB2 positive (**revised Supplementary Figure S1c, shown in reply to Reviewer #2 Concern #1**). Thus, although ILC1s in the small intestine exhibit high ASB2 expression, the fold change of ASB2 expression in ILC1s versus cNK cells is greater in the liver than in the intestine.

Furthermore, we agree that using 3 different datasets may introduce potential batch effects. To address this concern, we have re-analyzed the microarray data using a single dataset in the revised manuscript (GSE37448). As shown in Fig. R9a and Fig. R9b (**corresponding to the original and revised Fig. 1h, respectively**), the results obtained from the two analyses are consistent, and further confirmed that the fold change of *Asb2* expression between ILC1s and cNK cells is greater in the liver than in the intestine.

3) We have added a detailed description of the $Asb2^{EGFP}$ mouse strain in the Methods section of the revised manuscript **(revised Page 13, Line 427-430).** We apologize for this omission in the original version.

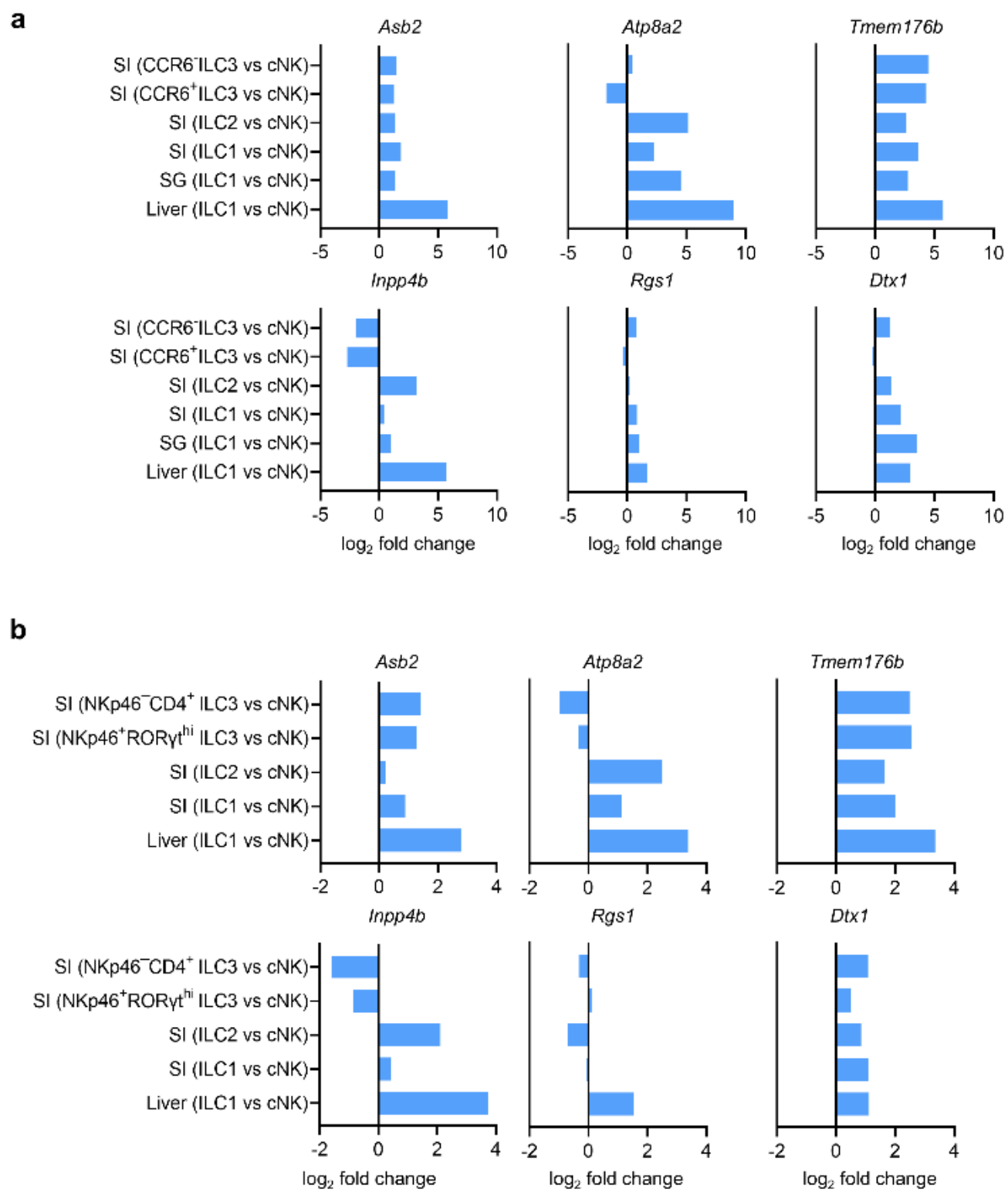

Fig. R9. The original and revised Fig. 1h in the manuscript.

(a) The $\log_2$ fold changes of indicated genes in ILC1s, ILC2s, and ILC3s from indicated tissues after normalization to cNK cells in the original manuscript by using 3 different datasets. **(b)** The $\log_2$ fold changes of indicated genes in ILC1s, ILC2s, and ILC3s from indicated tissues after normalization to cNK cells in the revised manuscript by using a single dataset.

2. The ASB2 gene encodes two transcripts that are translated into two protein isoforms that differ in their N-terminus, Isoform 1/ASB2β (Bello et al, Cell Death & Diff 2009; Davey et al, JCI Insight 2016; Jang et al, J Cachexia Sarcopenia Muscle 2025) and isoform 2/ASB2α (Guibal et al. J Biol Chem 2002; Metais et al, Circ Res 2018; Maire et al, Nat Commun 2024). Since these proteins exhibited different functional domains and have different substrates, the authors should investigate which isoform is expressed in ILC1s. This is of importance since the exon of ASB2 that is flanked with loxP sequences in the conditional knockout mouse models is common to both isoforms.

REPLY：

We appreciate the reviewer for this important comment. We sorted liver ILC1s and cNK cells from WT mice, and performed quantitative PCR analysis of ASB2α and ASB2β expression in these cells. We found that ILC1s predominantly express the ASB2α isoform, whereas neither isoform was expressed in cNK cells. We have added the relevant data in current version (**Fig. R10, revised Fig. 1k in the manuscript**). Moreover, the conditional ASB2-flox mouse line used in this study targets exon 4, which is common to both ASB2α and ASB2β transcripts. Therefore, deletion of this shared

exon ensures complete ablation of all functional ASB2 isoforms.

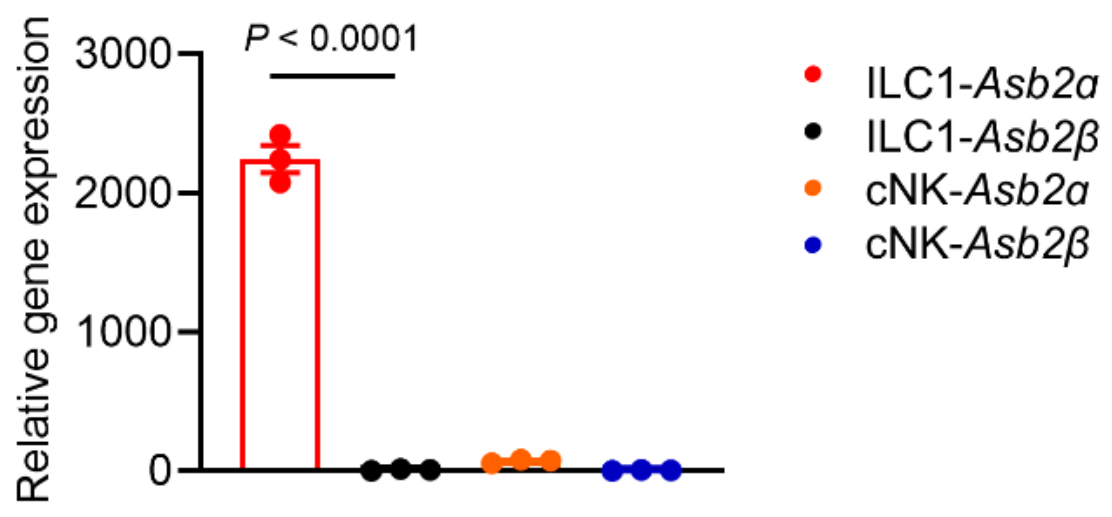

Fig. R10. Liver ILC1s exclusively express the ASB2α isoform.

Quantitative PCR analysis of *Asb2α* and *Asb2β* mRNA expression in liver ILC1s and cNK cells in WT mice ($n = 3$ per group). Data are from one experiment of two independent experiments. Samples were compared using one-way ANOVA and were presented as means ± SEM.

3. The authors have to validate the loss of ASB2 expression in ASB2 knockout cells.

REPLY：

Thanks for this suggestion. We validated the loss of *Asb2* in liver NK1.1^{+}NKp46^{+} cells, which consist of both cNK and ILC1s, from *Ncr1*$^{Cre/+}$*Asb2*$^{fl/fl}$ mice at the mRNA level (**Fig. R11, revised Supplementary Fig. 3a in the manuscript**). Taken together with our aforementioned data showing that cNK cells and ILC1s do not express *Asb2*, these results confirm efficient and specific ablation of *Asb2* in ILC1s.

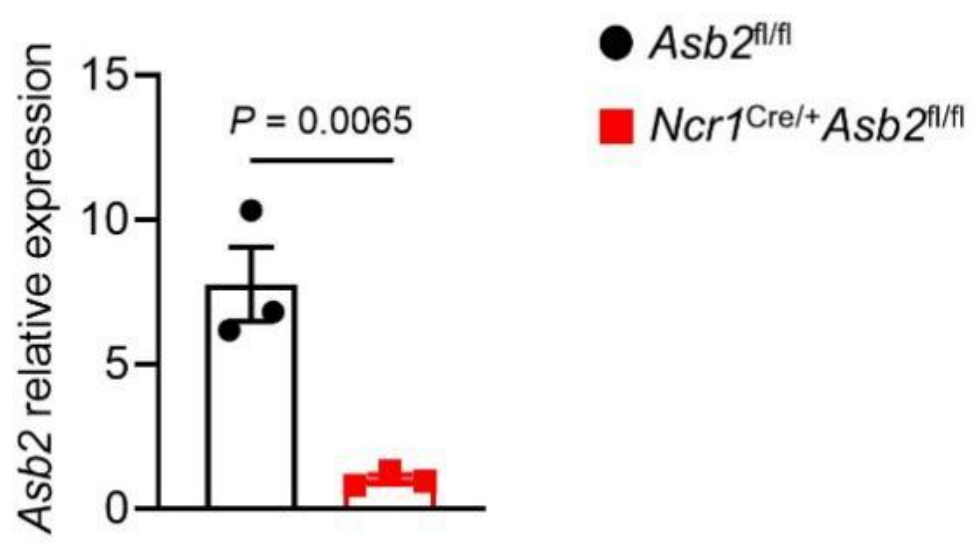

Fig. R11. Successful knockout of *Asb2* in NK1.1^{+}NKp46^{+} cells

Quantitative PCR analysis of *Asb2* expression in liver NK1.1^{+}NKp46^{+} cells from *Ncr1*$^{Cre/+}$*Asb2*$^{fl/fl}$ and *Asb2*$^{fl/fl}$ mice ($n = 3$ per group). Data are from one representative experiment of two independent experiments. Samples were compared using two-tailed unpaired Student's *t* tests and were presented as means ± SEM.

4. Ncr1-Cre heterozygous mice must be used as controls in all experiments because NKp46-expressing cells from heterozygous Ncr1-Cre mice were shown to display a significant reduction of NKp46 expression compared to those of mice that do not express the Cre, likely with consequences on ILC1 sorting. In addition, this may impact ILC1 activity as previously demonstrated in NK cells (Narni-Mancinelli, Science, 2012). Asb2$^{fl/fl}$ mice cannot be used as controls.

REPLY:

Thanks for pointing out this important issue. Considering the potential effect of Ncr1-Cre on ILC1s, we fully agree that *Ncr1*$^{Cre/+}$ mice are the ideal controls. Due to the limited time available for revision, we were unable to repeat all experiments using *Ncr1*$^{Cre/+}$ mice as controls. However, we performed a series of critical experiments using *Ncr1*$^{Cre/+}$ mice as controls, and obtained results similar to those using *Asb2*$^{fl/fl}$ littermate controls: 1) Liver ILC1 proportion and number were decreased in *Ncr1*$^{Cre/+}$*Asb2*$^{fl/fl}$ mice compared to *Ncr1*$^{Cre/+}$ controls, whereas cNK cells were unaffected (**Fig. R12a, b, revised Supplementary Fig. 4a, b in the manuscript**). 2) Reduced viability and increased lipid content were observed in *Ncr1*$^{Cre/+}$*Asb2*$^{fl/fl}$ mice, while no significant differences were found between *Ncr1*$^{Cre/+}$ and *Asb2*$^{fl/fl}$ control mice (**Fig. R12c, d, revised Supplementary Fig. 4c, d in the manuscript**). PA treatment further reduced liver ILC1 viability, with a more pronounced decrease in *Ncr1*$^{Cre/+}$*Asb2*$^{fl/fl}$ mice than in both controls (**Fig. R12e, shown as below**). 3) In the MC38 liver metastasis model, *Ncr1*$^{Cre/+}$*Asb2*$^{fl/fl}$ mice exhibited accelerated tumor growth, decreased tumor-infiltrating ILC1 number, and increased ILC1 lipid content compared with *Ncr1*$^{Cre/+}$ controls (**Fig. R12f-h, shown as below**). Taken together, key conclusions of this study were also confirmed using *Ncr1*$^{Cre/+}$ control mice, indicating that the observed changes in ILC1s form *Ncr1*$^{Cre/+}$*Asb2*$^{fl/fl}$ mice are mainly attributable to the loss of *Asb2*.

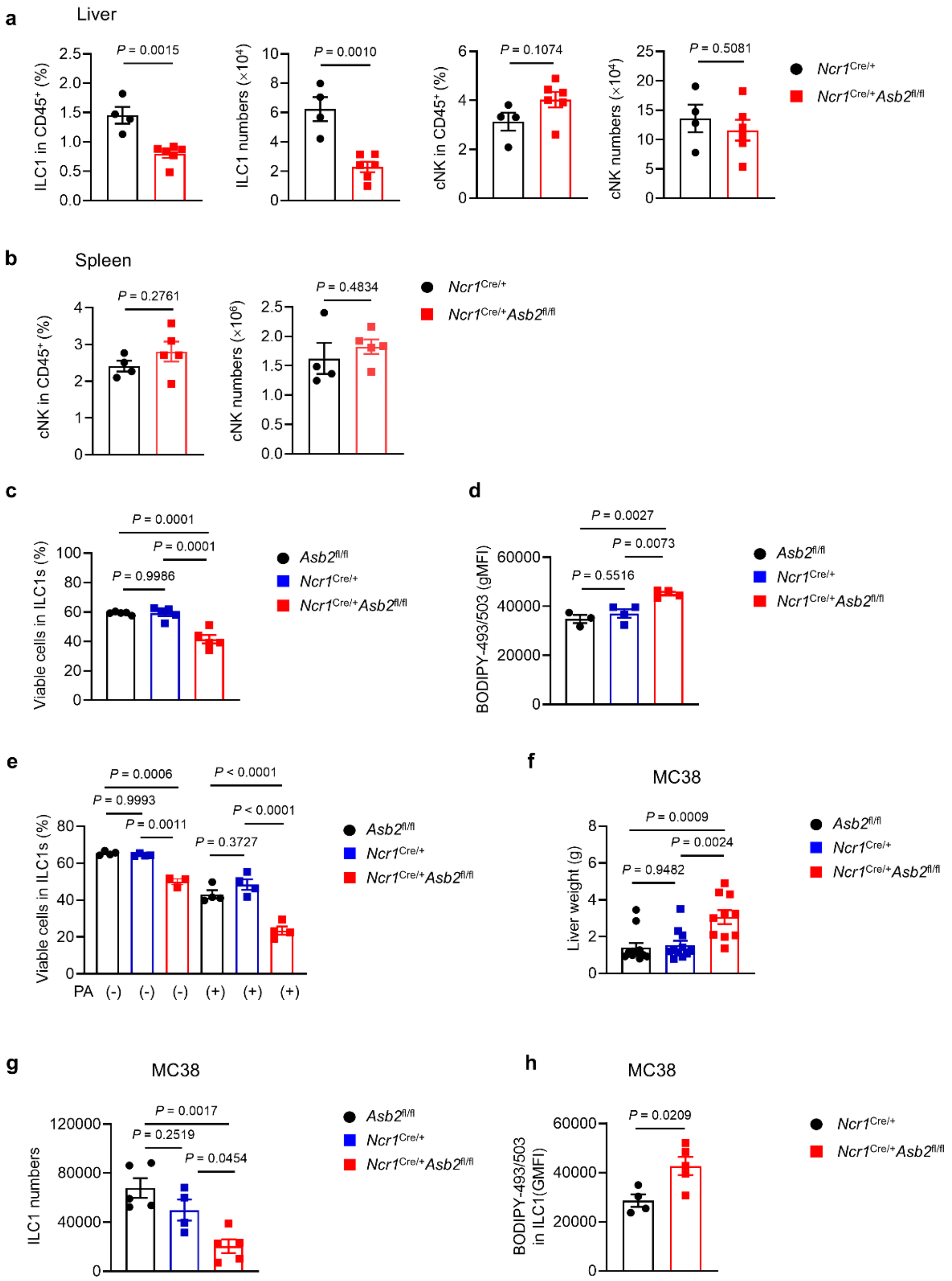

Fig R12. ASB2 deficiency in NKp46^{+} cells affect ILC1 activity and lipid metabolism independently of potential Cre toxicity.

(a) Percentages and numbers of liver ILC1s and cNK cells from *Ncr1*$^{\text{Cre/+}}$*Asb2*$^{\text{fl/fl}}$ (n = 6) and *Ncr1*$^{\text{Cre/+}}$ mice (n = 4). **(b)** Frequencies and absolute numbers of spleen cNK cells from adult *Ncr1*$^{\text{Cre/+}}$*Asb2*$^{\text{fl/fl}}$ (n = 5) and *Ncr1*$^{\text{Cre/+}}$ mice (n = 4). **(c)** Proportions of viable (AnnexinV^{-} 7-AAD^{-}) cells among liver ILC1s from adult *Ncr1*$^{\text{Cre/+}}$*Asb2*$^{\text{fl/fl}}$, *Ncr1*$^{\text{Cre/+}}$ and *Asb2*$^{\text{fl/fl}}$ mice (n = 5 per group). **(d)** gMFI of BODIPY-493/503 staining in ILC1s from *Ncr1*$^{\text{Cre/+}}$*Asb2*$^{\text{fl/fl}}$ (n = 4), *Ncr1*$^{\text{Cre/+}}$ (n = 4) and *Asb2*$^{\text{fl/fl}}$ mice (n = 3). **(e)** Percentages of viable cells (AnnexinV^{-} 7-AAD^{-}) of ILC1s that were

cultured with 100 μM palmitic acid (PA) for 24 h from adult *Ncr1*$^{Cre/+}$*Asb2*$^{fl/fl}$, *Ncr1*$^{Cre/+}$ and *Asb2*$^{fl/fl}$ mice (n = 3 or 4 per group). **(f-h)** liver weight (n = 11 *Ncr1*$^{Cre/+}$ group, n = 10 *Ncr1*$^{Cre/+}$*Asb2*$^{fl/fl}$, n = 12 *Asb2*$^{fl/fl}$ group) **(f)**, counts of ILC1s (n = 4 *Ncr1*$^{Cre/+}$ group, n = 5 *Ncr1*$^{Cre/+}$*Asb2*$^{fl/fl}$ and *Asb2*$^{fl/fl}$ group) **(g)**, BODIPY493/503 staining of liver ILC1s (n = 4 *Ncr1*$^{Cre/+}$ group, n = 5 *Ncr1*$^{Cre/+}$*Asb2*$^{fl/fl}$) **(h)** from *Ncr1*$^{Cre/+}$*Asb2*$^{fl/fl}$, *Asb2*$^{fl/fl}$ and *Ncr1*$^{Cre/+}$ mice, all of which were i.s. injected with 2×10^5 MC38-luc cells. Samples were compared using two-tailed unpaired Student's *t* tests **(a, b and h)** or one-way ANOVA **(c-g)** and were presented as means ± SEM.

5. GO enrichment analysis showed that genes involved in the negative regulation of cell adhesion are at the top of the list of genes up-regulated in liver ILC1s of *Ncr1*$^{Cre/+}$*Asb2*$^{fl/fl}$ mice. This is not mentioned, explored, nor discussed in the manuscript although ASB2 isoform 2 (ASB2α) was previously shown to regulate cell motility in immune cells (Lamsoul et al, Blood 2013; Maire et al, Nature Communications 2024). The authors should therefore investigate whether ASB2 loss in ILC1s has an impact on their homing and residency in the liver or in their distribution in other tissue.

REPLY：

Thanks for this insightful suggestion. We fully agree that ASB2 may regulate the residency of liver ILC1s. To address this, we performed inducible *Asb2* ablation using *Ncr1*$^{CreERT2/+}$*Asb2*$^{fl/fl}$ mice after expanding the liver ILC1 pool by hydrodynamical injection of pLive-IL-15 plasmids. Tamoxifen was injected for 5 consecutive days to induce *Asb2* ablation in NKp46$^+$ cells, and the proportion and number of ILC1s in the liver, spleen, and peripheral blood were analyzed 5 days after the final injection (**Fig. R13a, shown as below**). In the liver, we found a significant reduction in the proportion and number of ILC1s (**Fig. R13b, shown as below**); however, we did not detect a corresponding increase in ILC1s in the peripheral blood or spleen (**Fig. R13c, shown as below**), suggesting no obvious ILC1 egress from the liver under this condition. Although the present experimental setup did not reveal a major role for ASB2 in acute ILC1 migration or tissue redistribution, we cannot fully exclude potential functions in cell adhesion or long-term tissue residency. Further studies using more sophisticated approaches will be required to fully characterize the role of ASB2 in these processes, which we aim to pursue in future investigations. In the revised manuscript, we added discussion of this important point (**revised Page 8-9, Line 234-241**).

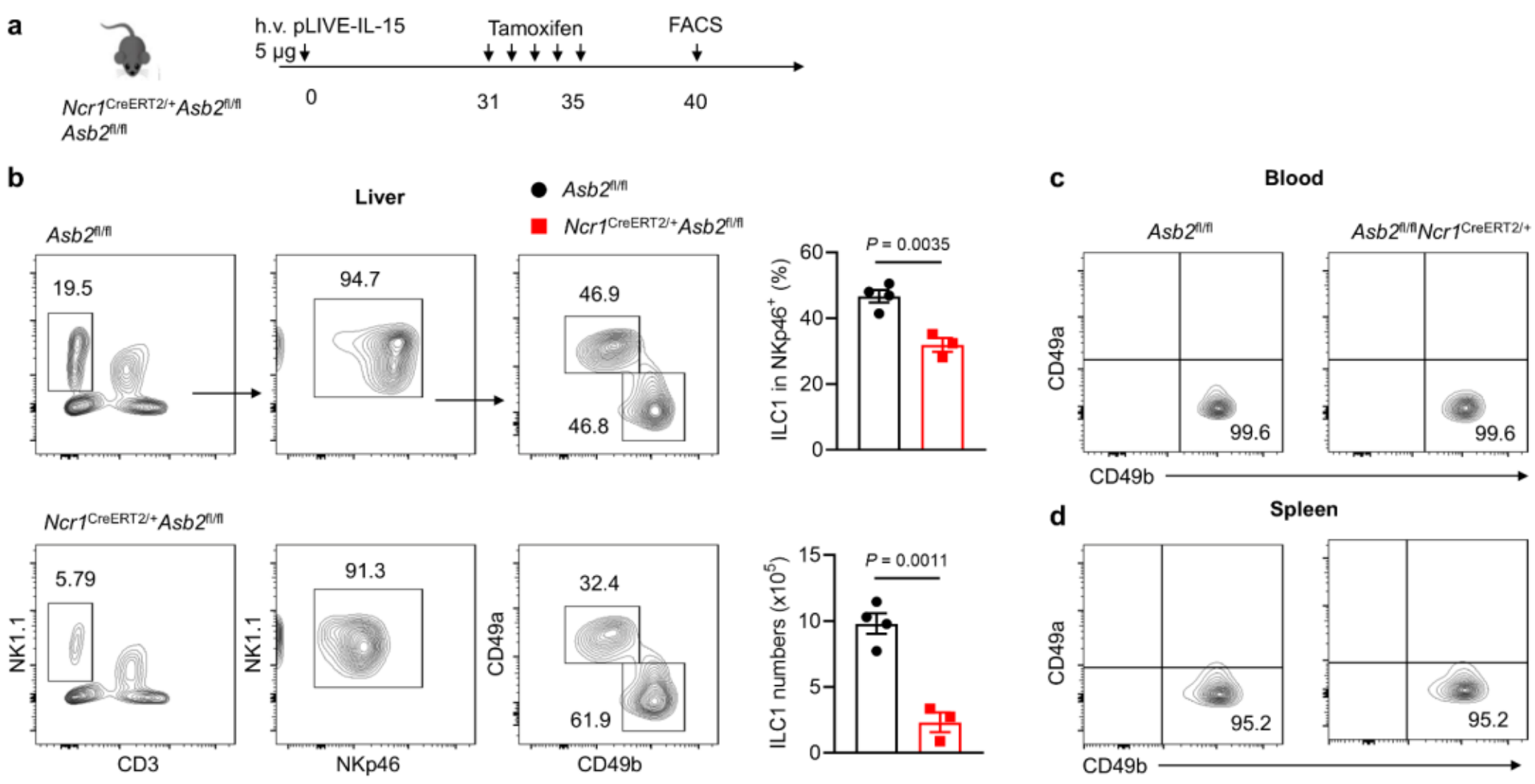

Fig R13. Inducible deletion of *Asb2* in NKp46^{+} cells fails to promote ILC1 egress from the liver.

(a) *Ncr1*$^{\mathrm{CreERT2/+}}$*Asb2*$^{\mathrm{fl/fl}}$ and *Asb2*$^{\mathrm{fl/fl}}$ mice were subjected to hydrodynamic tail vein injection with 5 μg pLive-IL-15 plasmid. Thirty days later, tamoxifen was administered intraperitoneally for 5 consecutive days. After an additional 5 days, flow cytometry was performed to analyze the proportion and number of ILC1s in the liver, spleen, and peripheral blood. **(b)** Representative flow cytometry plots, frequencies and absolute numbers of liver ILC1s from *Ncr1*$^{\mathrm{CreERT2/+}}$*Asb2*$^{\mathrm{fl/fl}}$ ($n = 3$) and *Asb2*$^{\mathrm{fl/fl}}$ mice ($n = 4$). **(c, d)** Representative flow cytometry plots of ILC1s in blood **(c)** and spleen **(d)** from *Ncr1*$^{\mathrm{CreERT2/+}}$*Asb2*$^{\mathrm{fl/fl}}$ ($n = 3$) and *Asb2*$^{\mathrm{fl/fl}}$ mice ($n = 4$). Samples were compared using two-tailed unpaired Student's *t* tests **(b)** and were presented as means ± SEM.

6. In the Volcano plots showed in Fig 3a, 1) it seems that some of the proteins that were the most highly differentially expressed were omitted. 2) What are the values provided in the proteomic table for protein abundances in the ILC1 samples? 3) How were the missing values treated? 4) What is the statistical test used to determine the differentially expressed proteins in the 2 KO vs the 3 control ILC1 samples? 5) Names of the differentially expressed genes / proteins found in the transcriptomic and proteomic analyses should be provided in a supplementary table. Genes of enriched pathways (GO transcriptomic analysis) should be provided in a supplementary table.

REPLY

Thanks for pointing out these important issues.

1) Regarding the volcano plots (**Fig. R14a, revised Fig. 3a**), we have now revised highlighted the most highly differentially expressed proteins, including ZFP523, RAB43, PRAG1.

2) The values provided in the proteomic table were derived from raw protein quantification values after FOT analysis. The fraction of the total (FOT), defined as a protein's Intensity-Based Absolute Quantification (iBAQ) divided by the total iBAQ of all identified proteins within one sample, thus

representing the normalized abundance of a particular protein across samples. Finally, the FOT values were further multiplied by 1×10^5 for ease of presentation. This eliminates systematic errors including sample loading amount and instrumental response, enabling cross-sample comparison of relative protein abundance.

3) Missing values were imputed using one-tenth of the minimum value in the raw dataset.

4) Due to the scarcity of ILC1s in *Asb2* cKO mice and technical challenges in isolating these cells, only two cKO samples were available for analysis. In the original version, we used *t-test* for statistical analysis. Considering the small sample size, in the revised manuscript, we instead used heatmaps to display expression of major enzymes involved in fatty acid uptake, fatty acid oxidation and lipogenesis in control and ASB2-deficient ILC1s **(Fig. R14b, revised Fig. 3c)**.

5) Finally, according to the suggestion, we have added supplementary tables listing the differentially expressed genes/proteins identified from transcriptomic and proteomic analyses, and genes involved in the enriched pathways from transcriptomic GO analysis (**revised Supplementary dataset 1-4**).

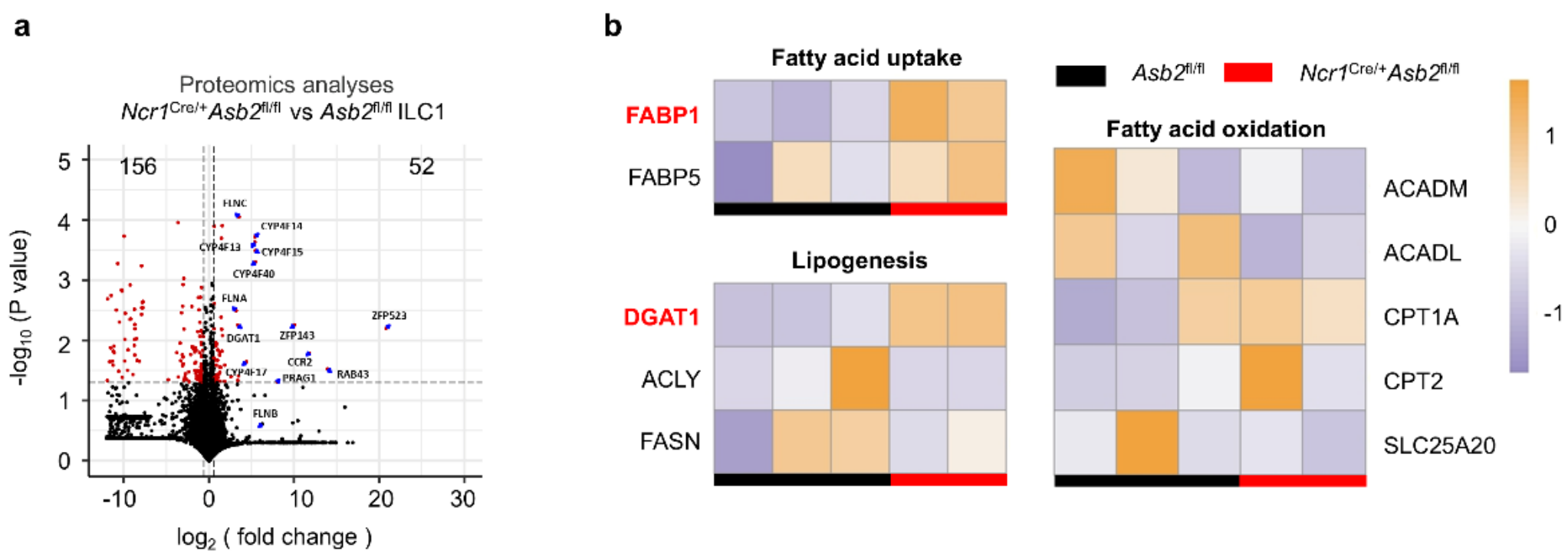

R14. Volcano plot and heatmap showing differentially expressed proteins

(a) Proteomic was performed on ILC1s ($CD45^+CD3^-CD19^-NK1.1^+NKp46^+CD49b^-CD49a^+$) isolated from livers in *Ncr1*$^{Cre/+}$*Asb2*$^{fl/fl}$ or *Asb2*$^{fl/fl}$ mice. Volcano plot showing differentially expressed proteins between ILC1s from *Ncr1*$^{Cre/+}$*Asb2*$^{fl/fl}$ and *Asb2*$^{fl/fl}$ mice. Lines indicate a 1.5-fold difference between ILC1 cells from *Ncr1*$^{Cre/+}$*Asb2*$^{fl/fl}$ and *Asb2*$^{fl/fl}$ mice ($p < 0.05$). **(b)** Heatmaps showing expression of enzymes involved in fatty acid uptake, fatty acid oxidation and lipogenesis in ILC1s from *Ncr1*$^{Cre/+}$*Asb2*$^{fl/fl}$ and *Asb2*$^{fl/fl}$ mice.

7. The functional link between ASB2 and ILC1 biology is missing and should be investigated. This requires identification of the ASB2 isoform that is expressed in liver ILC1s and elucidation of downstream mechanisms controlling ILC1 properties, including identification of the substrates whose ubiquitination mediates downstream events.

REPLY：

We sincerely appreciate the reviewer for this critical comment regarding the functional link between ASB2 and ILC1 biology. To address this, we performed the following experiments:

First, we identified the ASB2 isoform expressed in liver ILC1s and found that they specifically express the *Asb2α* isoform (**shown in reply to Concern #2**). Considering that ASB2 deficiency significantly increased DGAT1 protein expression, we speculated that ASB2α may directly bind to DGAT1. To test this, we then performed co-immunoprecipitation assays, which confirmed that ASB2α can directly interact with DGAT1 (**Fig. R15, revised Fig. 3g in the manuscript**). Therefore, these results, together with our DGAT1 inhibitor experiments, indicate that ASB2α maintains lipid homeostasis in liver ILC1s by targeting DGAT1, thus establishing a direct molecular mechanism linking ASB2 to ILC1 properties. However, whether the regulation of DGAT1 expression depends on ASB2 ubiquitin ligase activity remains to be further determined.

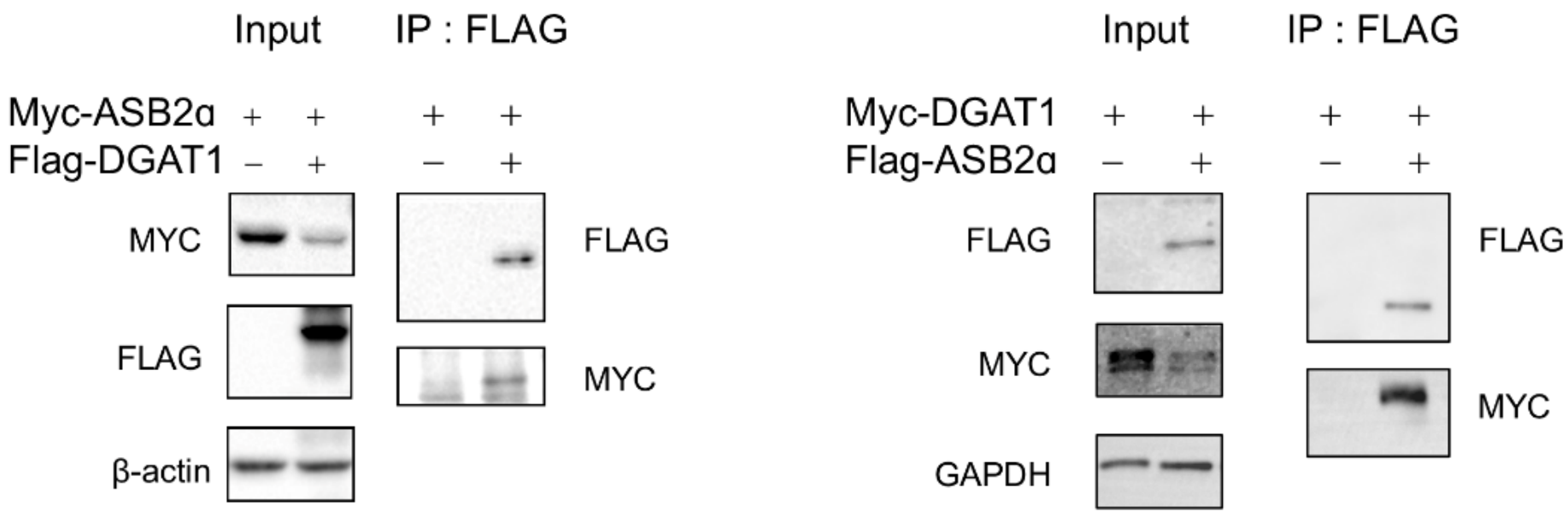

Fig R15. ASB2 can directly bind to DGAT1.

Co-Immunoprecipitation between ASB2α and DGAT1. IP and immunoblot analysis of the interaction between Myc-tagged ASB2α and Flag-tagged DGAT1 or Myc-tagged DGAT1 and Flag-tagged ASB2α.. The input is whole-cell lysates; n = 2 biologically independent experiments.

8. In its current form, this manuscript reveals a profound alteration of gene and protein expression following ASB2 deletion in liver ILC1s, associated with impaired cell survival. The authors concluded from the results shown in figure 4d and 5g that there is a functional link between ASB2 and the viability or the lipid content of ILC1s. However, these results are questionable. Indeed, the authors represented fold-changes (viable cells in liver ILC1s between *Asb2*$^{fl/fl}$ and *Ncr1*$^{Cre/+}$*Asb2*$^{fl/fl}$ mice and lipid contents in liver ILC1s between *Ncr1*$^{Cre/+}$*Asb2*$^{fl/fl}$ and *Asb2*$^{fl/fl}$ mice in tumor-bearing and non-tumor-bearing conditions, respectively), with statistical analyzes. How were these fold-changes calculated, particularly with different numbers of samples? If these fold-changes were calculated to relative means, statistical analyses could not be performed. Regarding the panel g of figure 5, the lipid contents in liver ILC1s of *Ncr1*$^{Cre/+}$*Asb2*$^{fl/fl}$ and control mice in tumor-bearing and non-tumor-bearing conditions should also be provided.

REPLY:

We sincerely appreciate the reviewer's careful evaluation of the statistical analyses. We fully agree that the presentation of fold changes might be problematic. In the revised manuscript, we have removed the fold change figures of lipid contents following stimulation in vitro (**Fig.4b, d in original version**) and retained the figures showing raw data of each group **(revised Fig.4a-d in the**

manuscript). We also removed original Fig. 5g. Of note, the corresponding raw data for lipid content have already been shown **(revised Fig.5f in the manuscript)** for tumor-bearing mice and for normal mice). These raw data are sufficient to support our conclusions.

9. The gender of the mice used in all the experiments is not specified. This is of major importance in many experiments, at least to study the impact of ASB2 deficiency in the anti-tumor function of ILC1s. Although it is indicated in the Methods section that "Mice were age-matched and sex-matched for each experiment", only 6 mice of each genotype were injected with MC38 cells in two independent experiments. This should be clarified.

REPLY:

Thanks for this suggestion. For steady-state experiments, age-matched female or male mice were used, with only one sex per experiment. For tumor experiments, age-matched male mice were primarily used, and replicate experiments performed in female mice yielded consistent results. In addition, we also sincerely appreciate your valuable comment concerning the number of mice and experimental repetitions used in the tumor model. Specifically, six mice of each genotype were used in each experiment, and all experiments were repeated twice. We agree that our original description was unclear, and we have now added information on sample size and replicates to all figure legends.

10. Throughout the manuscript, the authors indicated that statistical significance was performed using a two-tailed unpaired Student t test. However, this test can be performed only if each of the populations being compared follows a normal distribution. For sure, this cannot be stated from only 3 or 4 experiments. This is of importance since it questioned the statistical significance of almost all data.

REPLY:

We would like to express our sincere gratitude for your valuable comment on statistical analysis. We agree that appropriate statistical methods should be selected based on experimental design. Therefore, we re-analyzed the relevant data using one-way ANOVA (**revised fig. 1e, i, k**) or two-way ANOVA (**revised fig.4b, d, e, h, j; fig.5d, f**), and added the statistical description accordingly. All original conclusions remain fully supported and unchanged following this re-analysis.

Minor comments:

1. Some typographical errors have to be corrected.

REPLY:

Thanks for pointing out this. We have carefully checked the manuscripts and corrected typographical errors accordingly. All relevant modifications have been highlighted in the revised

manuscript.

2. The subtitle "ASB2 is specifically expressed in adult liver ILC1s" is an overinterpretation / simplification of the results.

REPLY:

Thanks for pointing out this important issue. The subtitle has been revised to "ASB2 is highly expressed in adult liver ILC1s" (**revised Page 2, Line 55**).

3. Samples from *Asb2*$^{fl/fl}$ mice should not be labeled as "WT".

REPLY:

Thanks for identifying this inaccuracy. We apologize for the oversight and have now carefully corrected the labeling throughout the manuscript. All samples from *Asb2*$^{fl/fl}$ mice are now properly labeled.

4. Related to data shown in figure 4f, g, treatment of ILC1s from control mice with palmitic acid and steric acid with or without A922500 should be performed.

REPLY:

Thanks for this valuable comment. In the revised manuscript, we have added the data of ILC1s from control mice treated with palmitic acid or stearic acid in the presence or absence of A922500 (**revised Fig. 4g-j in the manuscript**).

5. The survival curves of *Ncr1*$^{Cre/+}$*Asb2*$^{fl/fl}$ and *Asb2*$^{fl/fl}$ mice that received MC38 cells indicate that almost all mice are dying during the experiments. Is it ethically acceptable?

REPLY:

We sincerely appreciate the reviewer for this important ethical concern. All mouse experiments in this study were performed in accordance with approved animal care and use protocols and institutional ethical guidelines. For the metastatic MC38 model, the criteria for early endpoints included weight loss >20%, lethargy, hunched posture, abdominal distension. Euthanasia was performed by carbon dioxide inhalation immediately upon reaching any of these criteria. In the revised manuscript, we added methods of this important point **(revised Page 14, Line 456-458).**

Reviewer #1 (Remarks to the Author):

The authors have addressed my concerns.

REPLY:
We sincerely thank the positive feedback from the reviewer regarding our work.

Reviewer #2 (Remarks to the Author):

The revised manuscript has been improved, and the authors have addressed most of the previous comments. However, some issues remain and should be resolved before publication.

REPLY:
We thank the reviewer for the interest in our study and helpful comments. Our manuscript has been revised accordingly and greatly improved.

1. In the revised manuscript and response letter, the authors added data describing *Asb2* expression across multiple immune cell populations and tissues in the reporter mouse, which is helpful for the characterization of this newly generated strain. However, the new reporter mouse model has not been validated to great extent. It is important to confirm that the fluorescent reporter faithfully reflects endogenous Asb2 expression which has not been provided.

REPLY:
Thanks for the constructive suggestions. Flow cytometry analysis of *Asb2*^{EGFP} liver lymphocytes revealed that *Asb2* was highly expressed in ILC1s, moderately expressed in a small subset of CD4^{+} T cells, and barely detectable in cNK, CD8^{+} T and B cells (Fig. R1a, revised Fig 1j in the manuscript). To further validate that the fluorescent reporter faithfully reflects endogenous *Asb2* expression, we performed RT-qPCR analysis to examine two isoforms of *Asb2*, namely *Asb2α* and *Asb2β*, in different liver lymphocyte populations (ILC1s, cNK, T, and B cells) of WT mice. While *Asb2β* was negligibly expressed across all tested cell types, *Asb2α* was mostly highly expressed in ILC1s, with T cells exhibiting an intermediate level between ILC1s and other lymphocyte populations, which is in consistence with the flow cytometry results of *Asb2*^{EGFP} mice (Fig. R1b, revised Fig 1i in the manuscript). Therefore, these findings validate that the fluorescent reporter faithfully reflects the endogenous *Asb2* expression.

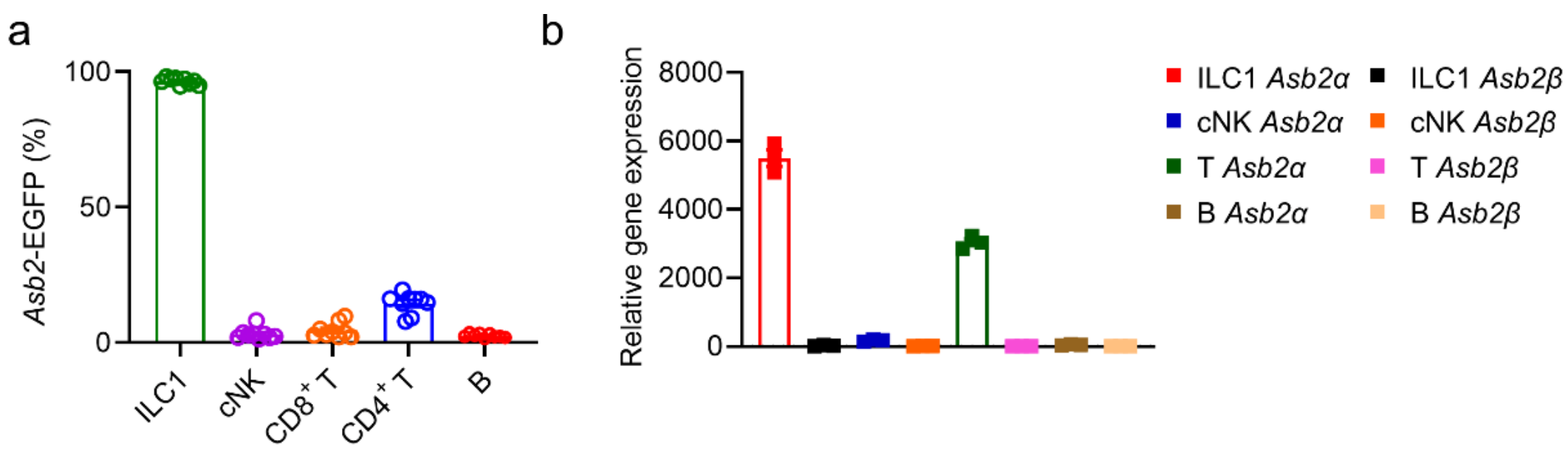

Figure R1 Fluorescent reporter accurately reports endogenous *Asb2* expression

(**a**) Frequencies of *Asb2*-EGFP expression on liver lymphocytes from adult $Asb2^{EGFP}$ mice (n = 9 per group). **(b)** Quantitative PCR analysis of *Asb2α* and *Asb2β* mRNA expression in liver ILC1s, cNK, T and B cells in WT mice (n = 3 per group). Gene expression is normalized to that of housekeeping gene *β-actin* and is presented relative to *Asb2β* in B cells. Data are from one representative experiment of two independent experiments**.** Samples were presented as means ± SEM.

2. Page 4, lines 91-95, the authors report that tamoxifen-induced deletion in $Ncr1^{CreERT2/+}Asb2^{fl/fl}$ mice reduces liver ILC1s (Figure 2d, e), but then refer $Vav^{Cre/+}Asb2^{fl/fl}$ mice to assess other lymphocyte populations (supplementary Figure 3c). The purpose of the $Vav^{Cre/+}$ model appears to be that *Asb2* does not affect other lymphocyte populations however the authors should also provide the ILC1 data in the same model.

REPLY:

Thanks for pointing out this important issue. In the $Vav^{Cre/+}Asb2^{fl/fl}$ mouse model, we consistently observed a significant reduction in both the proportion and number of liver ILC1s, and we have supplemented this results in the revised manuscript (Fig. R2, revised Supplementary Fig 3c in the manuscript).

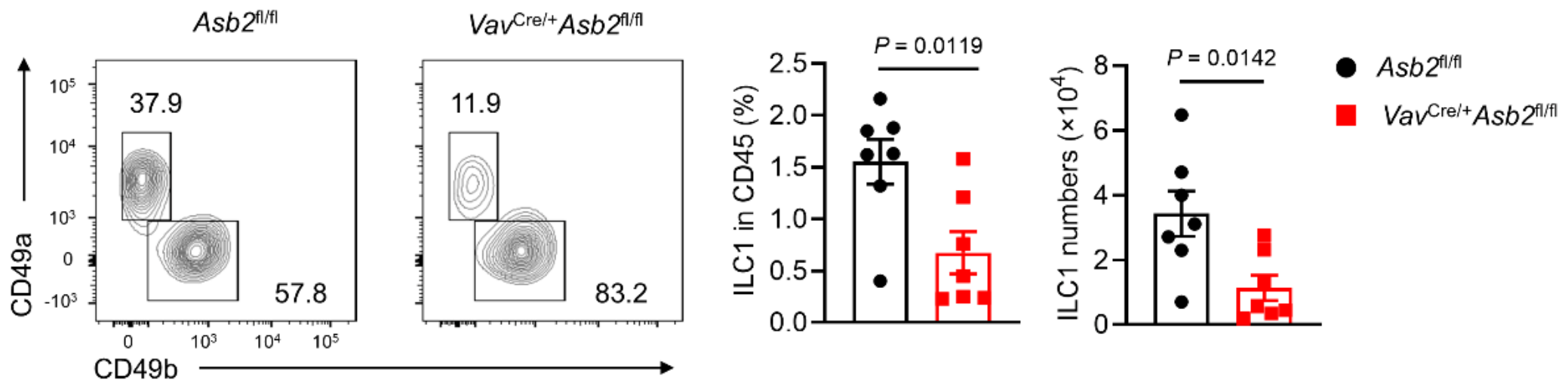

Figure R2 Liver ILC1s are reduced in $Vav^{Cre/+}Asb2^{fl/fl}$ mice

Representative plots (left) and percentages and absolute numbers (right) of liver ILC1s from $Vav^{Cre/+}Asb2^{fl/fl}$ and $Asb2^{fl/fl}$ mice (n = 7 per group). Data are pooled from two independent experiments. Samples were all compared using two-tailed unpaired Student's t tests and were presented as means ± SEM.

3. There appear to be multiple errors in supplementary figure citations throughout the revised manuscript.

For example:

Results section 2, "ASB2 supports the maintenance of adult liver ILC1s," Supplementary Fig. 2d, 2e appears to be incorrectly cited and should likely be Supplementary Fig. 3c, 3d-e, while Supplementary Fig. 3c should likely be Supplementary Fig. 4c.

Results section 3, "ASB2 inhibits lipid storage in ILC1s," references to Supplementary Fig. 4 appear to correspond to Supplementary Fig. 5.

Results section 4, "ASB2 enhances the anti-tumor activity of liver ILC1," references to Supplementary Fig. 5 appear to correspond to Supplementary Fig. 6, and Supplementary Fig. 6 should likely be Supplementary Fig. 7.

The authors should recheck all figure citations to ensure they are correct.

REPLY:
Thanks for pointing out these important issues. We are truly sorry and deeply apologize for the confusion caused by multiple errors in supplementary figure citations. This was an inadvertent error on our part. We have thoroughly rechecked all figure citations to ensure complete accuracy and consistency throughout the manuscript.

Reviewer #3 (Remarks to the Author):
I co-reviewed this manuscript with one of the reviewers who provided the listed reports. This is part of the Nature Communications initiative to facilitate training in peer review and to provide appropriate recognition for Early Career Researchers who co-review manuscripts.

Reviewer #4 (Remarks to the Author):
The manuscript has improved significantly and the authors critically addressed many points I raised. However, few issues still need to be fixed.

REPLY:
We thank both you and Reviewer #3 for the collaborative effort in evaluating our study. We appreciate the time and expertise you have contributed. Your insightful suggestions are helpful for us to improve the manuscript.

Major issues:

1. From the data provided in the new Fig. 1k, the authors concluded that "liver ILC1 exclusively express the ASB2alpha isoform". However, it is not clear how the relative expression between the two ASB2 isoforms in ILC1s and cNKs was calculated. This is absolutely required to support their conclusion.

REPLY:
We sincerely appreciate the reviewer's constructive comments on improving the methodology details. In accordance with the comments of **Reviewer #2 Concern #1**, the detection of *Asb2α* and *Asb2β* in liver T and B cells has been added to this section of the results. *Asb2α* and *Asb2β* expression was analyzed using the $2^{-\Delta\Delta CT}$ method. All transcriptional levels were first normalized to the housekeeping gene *β-actin*, and data are presented as fold changes relative to *Asb2β* expression in liver B cells. We have added this content to the Methods–RT-qPCR (revised Page 16, Line 513-515) and Figure 1i legend (revised Page 20, Line 633-635). Besides, given that our results reflect relative expression levels, we have softened our previous conclusion. The original claim that "liver ILC1s exclusively express the *Asb2α* isoform" has been changed to "liver ILC1s predominantly express the *Asb2α* isoform." (revised Page 3, Line 73-74)

2. There is still a major gap in mechanistic part of the revised manuscript. The authors showed co-immunoprecipitation of ASB2alpha with DGAT1 in transfected HEK cells, indicating an association between the two proteins (and not a direct interaction as concluded page 5, line 141). Because

DGAT1 is more abundant in ASB2-deficient cells and because ASB2alpha is known to exert its effects through polyubiquitination and degradation of its substrates, the authors should determine whether DGAT1 is a substrate of ASB2alpha ubiquitin ligase activity in ILC1s. If not, the functional link between ASB2alpha, DGAT1 and lipid metabolism in ILC1s still remains elusive.

REPLY:

We fully agree with the reviewer's insightful comments regarding the mechanistic limitation and statement accuracy, and we have carefully revised the corresponding descriptions and performed additional experiments to address these concerns.

First, as pointed out, our co-immunoprecipitation results in transfected HEK293T cells only demonstrate a physical association between ASB2α and DGAT1, rather than a direct protein interaction. We have strictly revised the relevant statement on Page 5, Line 145-148 (previous Page 5, Line 141) and throughout the manuscript, and all inappropriate claims of "direct interaction" have been corrected to "association" to ensure rigorous and objective conclusion.

Second, to explore whether DGAT1 serves as a ubiquitination substrate of ASB2, we have further performed ubiquitination assays in HEK293T cells co-expressing DGAT1 and ASB2α. However, overexpression of ASB2α did not enhance the ubiquitination level of DGAT1 in this system (Fig. R3), suggesting that ASB2-mediated regulation of DGAT1 may not rely on ubiquitination-dependent mechanisms.

Third, we acknowledge that the ideal experiment would be to examine endogenous DGAT1 ubiquitination in primary liver ILC1s. However, it is extremely challenging to obtain enough primary ILC1s (only ~10^4 cells per mouse) and genetic manipulation is also difficult for this cell type, making biochemical ubiquitination assays unfeasible in this cell type.

Notably, the major novelty and scope of the present study focus on uncovering the critical role of ASB2 in regulating liver ILC1s at the cellular and metabolic levels. The in-depth post-translational regulatory mechanism between ASB2 and DGAT1 represents a sophisticated and independent topic, which is beyond the main objective of this study.

Collectively, our current data sufficiently support the conclusion that ASB2 associates with DGAT1 and participates in modulating lipid metabolism in ILC1s. We have frankly discussed the existing mechanistic limitations and proposed relevant research perspectives in the revised Discussion section (revised Page 8, Line 219-228).

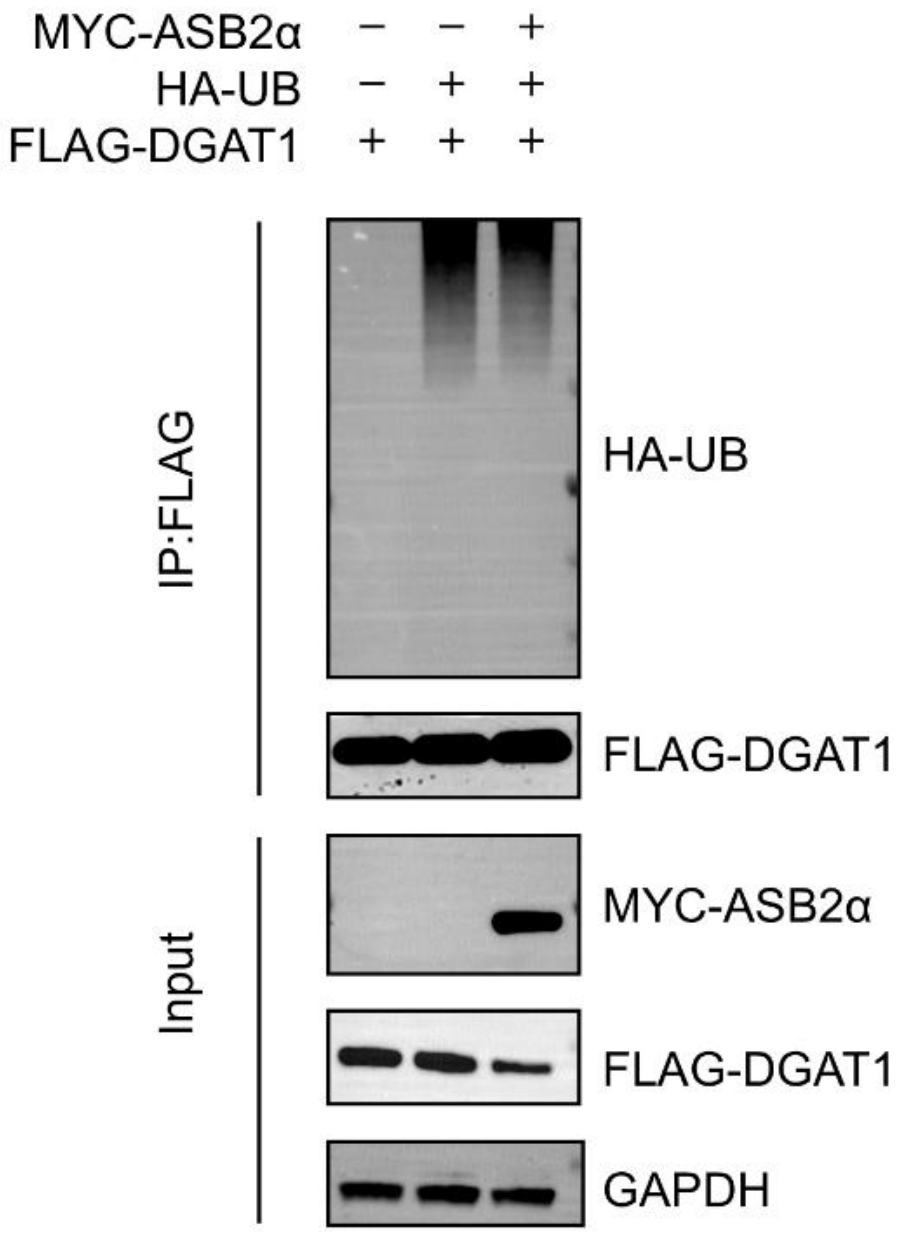

Figure R3. ASB2α does not promote DGAT1 ubiquitination.

HEK293T cells were co-transfected with MYC-ASB2α, HA-UB and FLAG-DGAT1. Cell lysates were immunoprecipitated (IP) with Flag M2 beads, followed by immunoblotting (IB) with anti-HA to detect ubiquitination of DGAT1. The input represents whole-cell lysates; $n = 2$ biologically independent experiments.

Minor comments:

1. In the description of the data obtained with the ASB2-EGFP mouse model, the authors have to clearly indicate in the result section that this model does not discriminate between the expression of the two ASB2 isoforms.

REPLY:

Thanks for pointing out this important issue. Original description "Then we generated an *Asb2*^{EGFP} reporter mouse line" has been revised to "Then we generated an *Asb2*^{EGFP} reporter mouse line, which cannot distinguish the expression of these two ASB2 isoforms" (revised Page 3, Line 75-76).

2. The authors should tone down their statement that ASB2 may be dispensable for early development of ILC1s because ASB2 was barely expressed in $CD45^+Lin^-$ mixed progenitors and $PD\text{-}1^+$ ILC precursors. Low expression does not mean no function!

REPLY:

Thanks for this suggestion. Considering that low expression does not rule out potential biological function, we have softened our conclusion. Original description "*Asb2* was barely expressed in $CD45^+Lin^-$ mixed progenitors and $PD\text{-}1^+$ ILC precursors (ILCP), suggesting that ASB2 may be dispensable for early development of ILC1s" has been revised to "*Asb2* was barely expressed in $CD45^+Lin^-$ mixed progenitors and $PD\text{-}1^+$ ILC precursors (ILCP), suggesting that ASB2 may exert limited influence on early development of ILC1s" (revised Page 4, Line 100-102).

3. In the main text, some figures are misnumbered.

REPLY:

We sincerely apologize for the incorrect ordering of the figures. We greatly regret this oversight. We have carefully reviewed all figure citations to guarantee their correctness.

REVIEWERS' COMMENTS

Reviewer #2 (Remarks to the Author):

Overall, this manuscript is significantly improved by the modifications.

REPLY:
We sincerely thank the positive feedback from the reviewer regarding our work.

Reviewer #3 (Remarks to the Author):

I co-reviewed this manuscript with one of the reviewers who provided the listed reports. This is part of the Nature Communications initiative to facilitate training in peer review and to provide appropriate recognition for Early Career Researchers who co-review manuscripts.

Reviewer #4 (Remarks to the Author):

The authors adequately addressed the different issues.

REPLY:
We would like to express our sincere gratitude to the reviewer #3 and reviewer #4 for the encouraging positive feedback on our work.
